# Exploration and verification of the therapeutic mechanism of shenfu injection in sepsis-induced myocardial injury

**DOI:** 10.1371/journal.pone.0317738

**Published:** 2025-01-17

**Authors:** Hua-jing Yuan, Guo-han Xiang, Yang Liu, Yan Li, Wen-li Liu, Jiu-xiang Wei, Yi-tao Xue, Hao Hao

**Affiliations:** 1 First School of Clinical Medicine, Shandong University of Traditional Chinese Medicine, Jinan, Shandong, PR China; 2 Intensive Care Unit, Affiliated Hospital of Shandong University of Traditional Chinese Medicine, Jinan, Shandong, PR China; 3 Department of Cardiology, Affiliated Hospital of Shandong University of Traditional Chinese Medicine, Jinan, Shandong, PR China; 4 College of Traditional Chinese Medicine, Shandong University of Traditional Chinese Medicine, Jinan, Shandong, PR China; 5 Postdoctoral Mobile Station, Shandong University of Traditional Chinese Medicine, Jinan, Shandong, PR China; Duke University Medical Center: Duke University Hospital, UNITED STATES OF AMERICA

## Abstract

**Background:**

Shenfu injection (SFI), derived from a traditional Chinese medicine (TCM) prescription, is an effective drug for the treatment of sepsis-induced myocardial injury (SIMI) with good efficacy, but its exact therapeutic mechanism remains unclear.

**Methods:**

SwissTargetPrediction and GeneCards database were used to obtain relevant targets for SFI and SIMI. STRING 11.5 and MCODE were used to analyse potential therapeutic targets for SFI. DAVID 6.8 database was used to perform enrichment analysis. In addition, the SIMI model was constructed by cecal ligation and puncture (CLP) on Sprague Dawley rats and the related protein expression levels were verified by AutoDock Vina 1.1.2 and experiment.

**Results:**

SFI has a total of 10 main active compounds and treats SIMI through 52 potential targets, among which LGALS3, STAT3, FGF1, and AKT1 were the core targets for treatment. Based on enrichment analysis, STAT3, FGF1, and AKT1 in the core targets were experimentally validated. The experimental results showed that SFI effectively ameliorated the inflammatory response and myocardial injury and inhibited apoptosis in SIMI. And SFI improved SIMI by decreasing caspase-9, STAT3 and phospho-AKT1 (p-AKT1) expression, and enhancing FGF1 expressions.

**Conclusions:**

This study showed that SFI effectively reduced the expression of caspase-9, STAT3 and p-AKT1, enhanced the expression of FGF1, reduced the inflammatory response, inhibited apoptosis and attenuated cardiac injury to SIMI.

## Introduction

Sepsis is a life-threatening disease caused by a dysregulated immune response to infection, which can lead to a series of organ dysfunctions [1, 2]. Although sepsis has received increasing attention in recent years, it remains a major public health problem worldwide with high morbidity and mortality [3]. Sepsis-induced myocardial injury (SIMI) is a common complication of sepsis and an important cause of death in sepsis patients [4, 5]. However, research on SIMI remains limited, and its clinical treatment also lacks the consensus of experts. Therefore, it is necessary to explore effective treatments for SIMI.

Shenfu injection (SFI) is an effective drug for the treatment of sepsis with a good curative effect. It is extracted from Chinese herbal medicine fuzi (*Aconiti Lateralis Radix Praeparata*) and hongshen (*Ginsen Radix Et Rhizoma Rubra*), and comes from traditional Chinese medicine (TCM) prescription Shenfu Decoction. A number of clinical and experimental studies have shown that SFI can effectively improve the hemodynamics of sepsis [6], regulate immune function [7–9], and improve clinical prognosis [6, 7, 9]. Furthermore, SFI can inhibit apoptosis of cardiomyocytes, reduce the inflammatory response, and prevent SIMI [10–12]. A meta-analysis based on 50 studies (3394 participants) showed that SFI was superior in reducing inflammation and improving mortality in patients with sepsis compared with other TCM injections [13]. Compared with routine medical treatment, SFI combined with routine medical treatment can enhance immune function and improve the prognosis of septic shock [14, 15]. In addition, an animal experiment study using 198 mice demonstrated that the efficacy of SFI in SIMI was similar to that of dexamethasone [16].

Although many pieces of evidence support that SFI has a good therapeutic effect on SIMI, the specific therapeutic mechanism of SFI is still unclear due to the multi-component, multi-target, and multi-pathway characteristics of TCM, which limits its clinical application and development. Therefore, we used network pharmacology to explore the active compounds and therapeutic mechanisms of SFI in the treatment of SIMI. Network pharmacology combines traditional pharmacology, computer technology and other disciplines, which can display the “drug-compound-target-disease” network, further demonstrating the interaction between drugs and diseases [17]. This study used network pharmacology to explore the therapeutic mechanism of SFI in SIMI, and verified it through molecular docking and experiments.

## Materials and methods

### Screening of main active compounds in SFI

Through a search of PubMed database (www.ncbi.nlm.nih.gov/pubmed), we obtained the main components of SFI in a recent ultra-high performance liquid chromatography-triple quadrupole tandem mass spectrometry (UHPLC-QQQ MS) study, which also validated the main components of SFI in plasma [18]. The web tool SwissADME (www.swissadme.ch) was used to calculate the physicochemical properties of SFI compounds and to evaluate their drug-likeness [19]. Among them, Lipinski’s Rule of Five (RO5) is considered to be an important prerequisite for ideal drug molecules [20]. Compounds that comply with RO5 are more likely to be drugs, as they have better pharmacokinetics and bioavailability. Therefore, among the compounds of SFI, those that comply with RO5 were considered as the main active compounds and used for further research and analysis.

### Screening SFI targets associated with SIMI

SwissTargetPrediction (http://www.swisstargetprediction.ch), a web tool that can effectively predict the relevant targets of molecular compounds, was used to predict the relevant targets of active compounds in SFI [21]. Specifically, we used the PubChem database (https://pubchem.ncbi.nlm.nih.gov) to obtain Canonical SMILES of active compounds in SFI and input them into SwissTargetPrediction, selecting Homo sapiens as the species, to obtain relevant targets of the active compounds in SFI.

GeneCards (https://www.genecards.org) is a comprehensive human gene database that provides data including genomics, proteomics, and clinical data [22]. Therefore, we used the keywords “sepsis-induced myocardial injury” and “septic cardiomyopathy” to obtain targets related to SIMI in the GeneCards database. Venny 2.1.0 (http://bioinfogp.cnb.csic.es/tools/venny) was used to obtain common SFI targets and SIMI targets, which are potentially relevant targets in the therapeutic mechanism of SFI for SIMI.

### Protein-protein interactions network

To identify the core targets of SFI for the treatment of SIMI, we used Protein-Protein Interactions (PPI) to analyze the therapeutic targets of SFI. We entered the therapeutic targets of SFI into the STRING 11.5 database (https://string-db.org), selected Homo sapiens as the species, and set the minimum interaction score to 0.4 to obtain the result of PPI analysis. Then, we used Cytoscape 3.7.2 to construct the PPI network, and used its NetworkAnalyzer tool to calculate the degree values of the targets.

MCODE can reclassify complex networks according to the relationship between edges and nodes to find key sub-networks and core targets. Therefore, we performed MCODE analysis of PPI network by Metascape (http://metascape.org) to find core targets.

### Enrichment analysis

To further explore the therapeutic mechanism and biological significance of SFI active compounds for SIMI, we used the DAVID 6.8 database (https://david.ncifcrf.gov/summary.jsp) to perform enrichment analysis including GO biological process and KEGG pathway on potential therapeutic targets of SFI. Among them, Homo sapiens was selected as the species, and gene was selected as the identifier and type. Enrichment analysis results with P adjust value <0.05 indicated statistical significance, which was used for further analysis.

### Molecular docking

Molecular docking can predict proteins and small molecules interactions and their binding affinities. AutoDock Vina 1.1.2 was used to validate the interaction of core targets with key compounds. Specifically, the RCSB PDB database (https://www.rcsb.org) [23] was used to obtain the crystal structures of the core targets, and the TCMSP database (https://tcmsp-e.com) [24] was used to obtain the molecular structures of the active compounds of SFI.

AutoDockTools 1.5.6 was used to process proteins and small molecules, selecting them as ligands and receptors. The docking method of proteins and small molecules adopted blind docking [25]. AutoDock Vina 1.1.2 was used to simulate the docking of proteins with small molecules and calculate their binding affinities. PyMol 2.5.2 was used to visualize the results of molecular docking.

### Shenfu injection

SFI (batch number: Z5120664) is an injection extracted from fuzi and hongshen, purchased from Ya’an Sanjiu Pharmaceutical Group Co., Ltd. (Sichuan Province, China). Its production quality meets the standards of China Food and Drug Administration (batch number: YBZ02062004).

### Animals

This study was conducted in accordance with the guide for the Guide for the Care and Use of Laboratory Animals and approved by the Experimental Animal Management Committee and the Ethics Committee of the Affiliated Hospital of Shandong University of Traditional Chinese Medicine (2020–44). A total of 24 Sprague Dawley rats (male, SPF grade, 200-220g) were purchased from Beijing Vital River Laboratory Animal Technology Co., Ltd. (license number: SCXK 2021–0011). All rats were housed in the Experimental Center of the Affiliated Hospital of Shandong University of Traditional Chinese Medicine (60% humidity, 24°C, 12 h light/dark cycle, free access to water and diet). All rats were randomly divided into sham-operation group (n = 8), SIMI group (n = 8) and SFI group (n = 8).

The SIMI model was achieved by cecal ligation and puncture (CLP) surgery. Specifically, amobarbital sodium (30 mg/kg, ip) was used to anesthetize rats. After preparation of the rat abdominal skin and disinfection with iodine, an incision was made in the mid-abdomen and the cecum was exposed. Sterile 4-gauge wire was used to ligate one third of the cecum, and an 18-gauge needle was used to perform 2 punctures in the middle of the ligated cecum, followed by gentle squeezing of the cecal ligation site until a small amount of feces appeared. The cecum was then put back into the abdominal cavity, and the abdominal incision was sutured layer by layer and disinfected. For rats in the sham-operation group, the cecum was isolated in the same experimental procedure, but without ligation and puncture. After the post-procedure, the three groups of rats were immediately injected with 10 mL of 0.9% normal saline (NS) to resist shock. Rats in the sham-operation and SIMI groups were intraperitoneally injected with 0.9% NS 10 mL/kg, and the rats in the SFI group were intraperitoneally injected with SFI 10 mL/kg.

After 24h postoperatively, the rats died from cervical dislocation after anesthesia with amobarbital sodium, which was chosen because of its rapid action and minimal pain caused to the animals. To minimize potential pain and suffering, rats were closely monitored during and after surgery, and additional care was taken to reduce their stress and promote recovery. The Animal Ethics Committee reviewed the entire experimental procedure and ensured that the experimental rats were handled following relevant welfare policies.

### Measurement of brain natriuretic peptide (BNP), interleukin-6 (IL-6) in myocardial tissues

Myocardial tissues were homogenized in PBS by tissue grinder processing, and further lysed by repeated freeze-thawing 2 times, and subsequently centrifuged at 12,000 rpm for 15 minutes to obtain the supernatant. A small amount of supernatant was used to determine protein concentration, which was measured using the BCA protein assay kit (P0010, Beyotime, China). The concentration of BNP (CSB-E07972r, CUSABIO, China) and IL-6 (70-EK306/3-48, MULTI SCIENCES, China) in myocardial tissues were measured by ELISA kits. Specifically, diluted samples were incubated, washed and color developed following the assay procedure described by the kit manufacturers. Absorbance at 450 nm was measured using a microplate reader and sample concentrations were calculated using standard curves to obtain BNP and IL-6 levels in myocardial tissues.

### Hematoxylin-eosin (H&E) staining

Fresh rat myocardial tissues were fixed in 4% paraformaldehyde, embedded in paraffin, and cut into 5-μm sections. The prepared rat heart sections were stained with hematoxylin-eosin (H&E) according to standard protocols. Histopathological changes in the myocardium of SIMI rats were observed by the digital section scanner (WS-10, WISLEAP, China).

### Terminal deoxynucleotidyl transferase dUTP nick-end labeling (TUNEL) staining

TUNEL staining was performed on the above prepared tissue sections according to the standard protocol by adding TUNEL detection solution and incubating at 37°C for 60 minutes under light, then rinsing with PBS. Cell nuclei were counterstained with DAPI and relevant images were subsequently acquired.

### Western blot analysis

Myocardial tissues were lysed and homogenized in RIPA buffer by tissue grinder processing, and the supernatant was obtained by centrifugation at 12,000 rpm for 20 minutes. A small amount of supernatant was used to determine protein concentration, which was measured by the BCA protein assay kit (P0010, Beyotime, China). Equal amounts of protein samples were separated by SDS-PAGE (P0688, Beyotime, China) and transferred to PVDF membranes (FFP24, Beyotime, China). Membranes were blocked in 5% nonfat dry milk in TBST at room temperature and then incubated for 12 hours at 4°C with primary antibodies: caspase-9 antibody (66169-1-Ig, Proteintech, China), FGF1 antibody (EPR19989, abcam, USA), AKT1 antibody (2938, Cell Signaling Technology, USA), Phospho-AKT1 (p-AKT1) antibody (9018, Cell Signaling Technology, USA), STAT3 antibody (60199-1-Ig, Proteintech, China) and β-actin antibody (60004-1-Ig, Proteintech, China). Membranes were then incubated with secondary antibodies for 1 hour at room temperature and analyzed by the ChemiDoc Imaging System (BIO-RAD, USA). ImageJ was used to quantify the results of the western blot.

### Statistical analysis

GraphPad Prism software (Inc., La Jolla, CA, USA) was used to perform statistical analysis of the data. All data were described as mean ± standard deviation (SD), and statistical differences between groups were compared by Student’s t-test or one-way analysis of variance (ANOVA). P value <0.05 indicated statistical significance.

## Results

### Main active compounds of SFI

**Fig 1:**
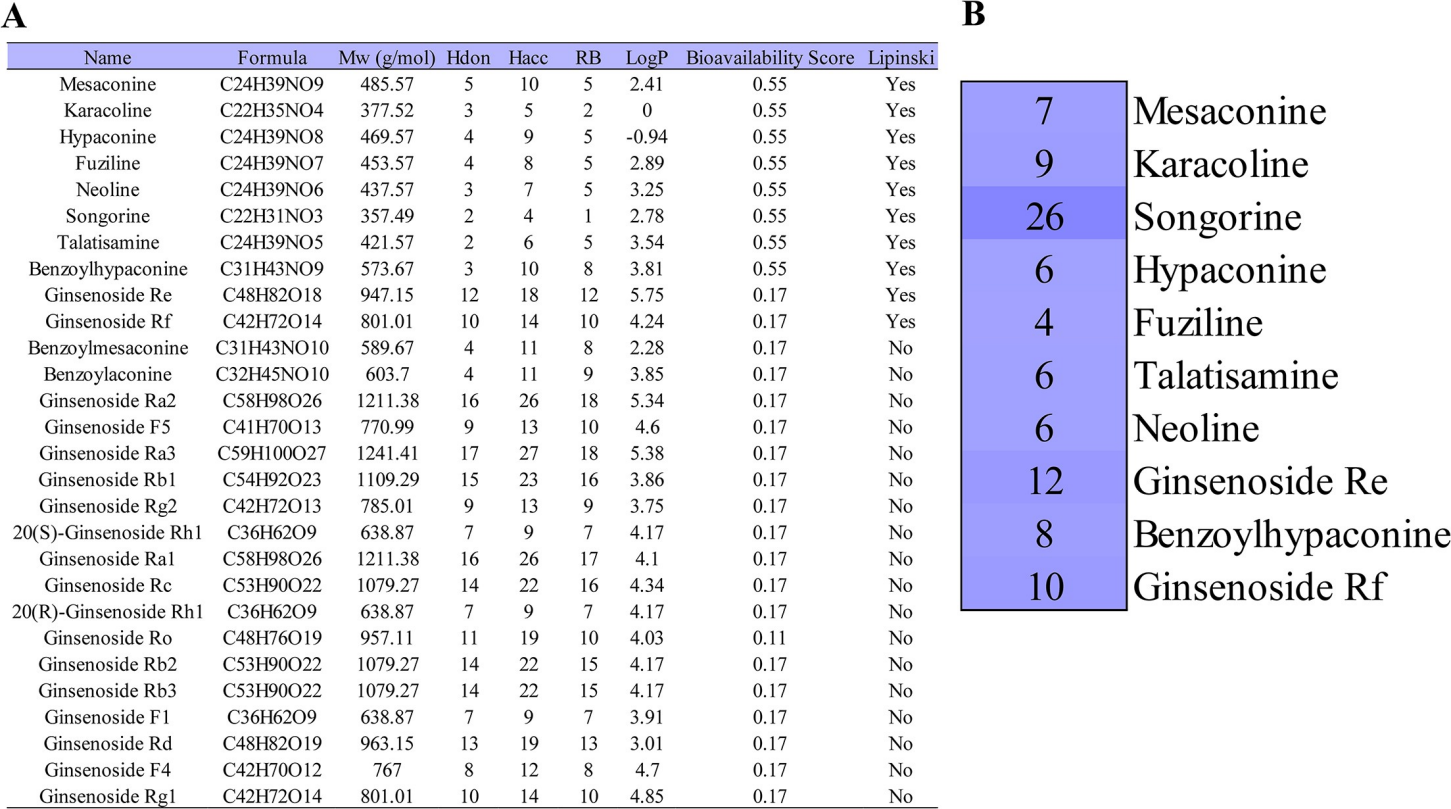
Main active compounds of Shenfu injection (SFI). (A) Physicochemical properties of active compounds in SFI. (B) Distribution of SIMI-related targets in SFI main active compounds.

We obtained 28 main compounds of SFI in a recent UHPLC-QQQ MS study (15). SwissADME was used to calculate the physicochemical properties of SFI compounds and to evaluate their drug-likeness (Fig 1A). Among them, RO5 is an important condition for evaluating ideal drug-like compounds. According to SwissADME predictions, 10 compounds complied with RO5, including 8 aconite alkaloids and 2 ginsenosides. Specifically, mesaconine, karacoline, hypaconine, fuziline, neoline, songorine, talatisamine, benzoylhypaconine, ginsenoside Re and ginsenoside Rf were considered as the main active compounds of SFI.

### Screening SFI targets associated with SIMI

The Canonical SMILES of the active compounds of SFI, which were retrieved from the PubChem database, were input into SwissTargetPrediction. After selecting Homo sapiens as the species, we obtained 129 SFI-related targets. In addition, we obtained 1584 and 777 targets in the GeneCards database using “sepsis-induced myocardial injury” and “septic cardiomyopathy” as the keywords, respectively. After summarizing all SIMI targets, we obtained a total of 1729 SIMI-related targets.

We used Venny 2.1.0 to intersect SFI targets with SIMI-related targets and obtained 52 common targets of SFI and SIMI, which were potential therapeutic targets of SFI for SIMI. S1 Table in S1 File presented specific information on 52 common targets. And Fig 1B showed the number of targets for each active compound of SFI. Among these compounds, songorine has the most potential therapeutic targets (26 targets) related to SIMI, followed by ginsenoside Re (12 targets) and ginsenoside Rf (10 targets), which indicates that these compounds may be the key components of SFI treatment of SIMI.

### Protein-protein interactions network

**Fig 2:**
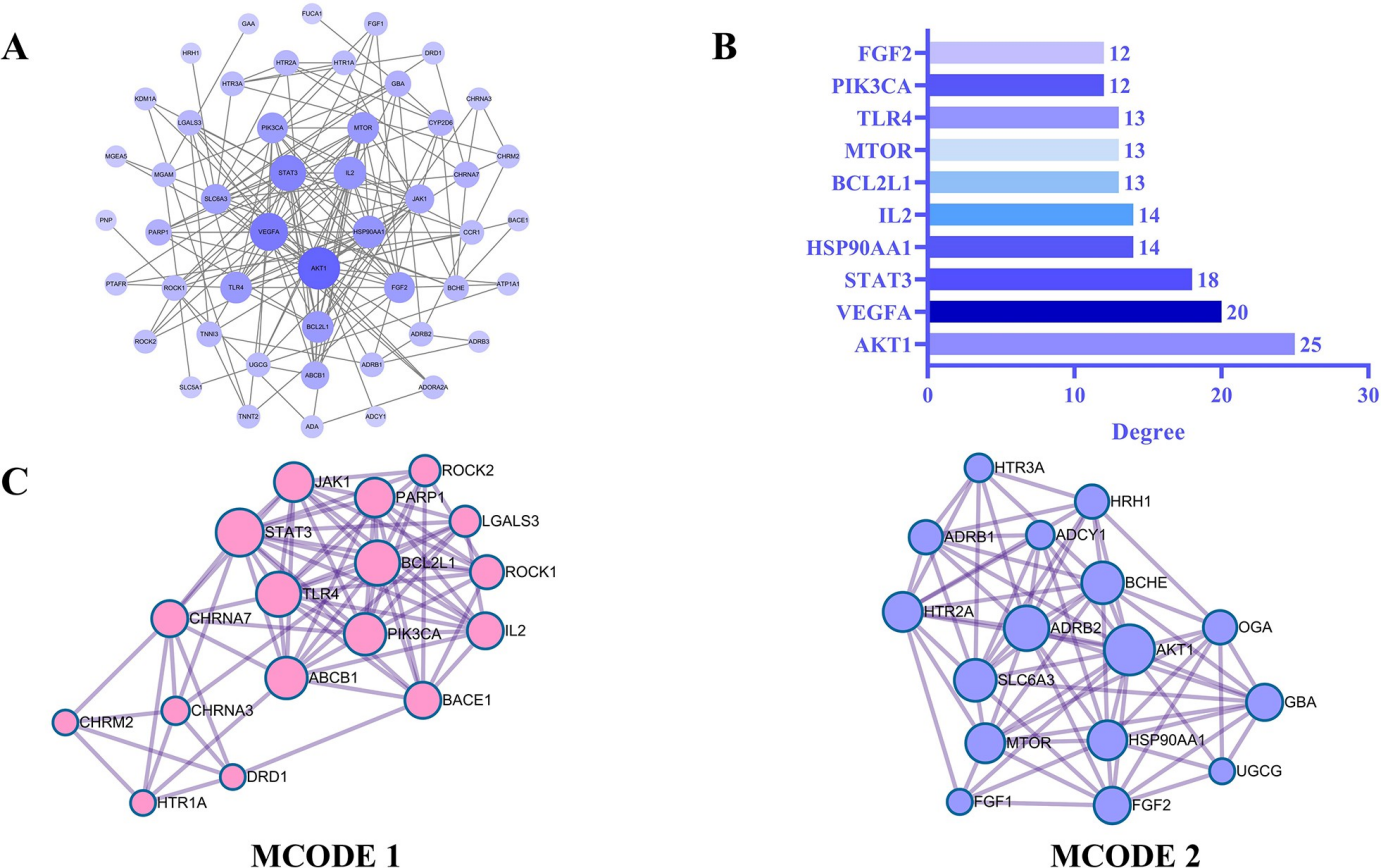
PPI network. (A) PPI networks constructed from potential therapeutic targets of SFI for SIMI. The size and color of nodes were positively related to degree. (B) The top ten targets in the PPI network ranked by degree. (C) 2 clusters of MCODE analysis.

To explore the interactions of potential therapeutic targets and search for core targets, we performed PPI analysis of potential therapeutic targets by the STRING 11.5. By visualization with Cytoscape 3.7.2, we got a PPI network with 49 nodes and 164 edges (Fig 2A). According to the calculation of the NetworkAnalyzer tool, the top ten targets ranked by degree values were AKT1, VEGFA, STAT3, HSP90AA1, IL2, BCL2L1, MTOR, TLR4, PIK3CA and FGF2 (Fig 2B).

Based on the MCODE analysis of the PPI network implemented by Metascape, we obtained 2 clusters (Fig 2C). Table 1 presented the pathway and process enrichment analysis results for each MCODE cluster. MCODE analysis showed that in MCODE 1, LGALS3 was the seed target and STAT3 had the highest degree value. In MCODE 2, FGF1 was the seed target and AKT1 had the highest degree value. Therefore, LGALS3, STAT3, FGF1 and AKT1 were considered to be the core targets of SFI in the treatment of SIMI. In addition, the cAMP signaling pathway may be the potential mechanism of MCODE 1 in the treatment of SIMI, while the calcium signaling pathway may be the potential mechanism of MCODE 2 in the treatment of SIMI.

**Table 1:** Pathway and process enrichment analysis of the MCODE clusters.

MCODE	Term	Description	Log10(P)
MCODE 1	hsa05162	Measles	-10
MCODE 1	hsa04024	cAMP signaling pathway	-8.8
MCODE 1	hsa05200	Pathways in cancer	-8.1
MCODE 2	hsa05207	Chemical carcinogenesis—receptor activation	-11.1
MCODE 2	hsa04020	Calcium signaling pathway	-10.7
MCODE 2	hsa04923	Regulation of lipolysis in adipocytes	-7.7

### Enrichment analysis

We performed enrichment analysis including GO biological process and KEGG pathway for 52 potential therapeutic targets of SFI using the DAVID 6.8 database to further analyze their therapeutic mechanisms and biological significance. The results of enrichment analysis, with a P adjust value < 0.05, indicated statistical significance.

**Fig 3:**
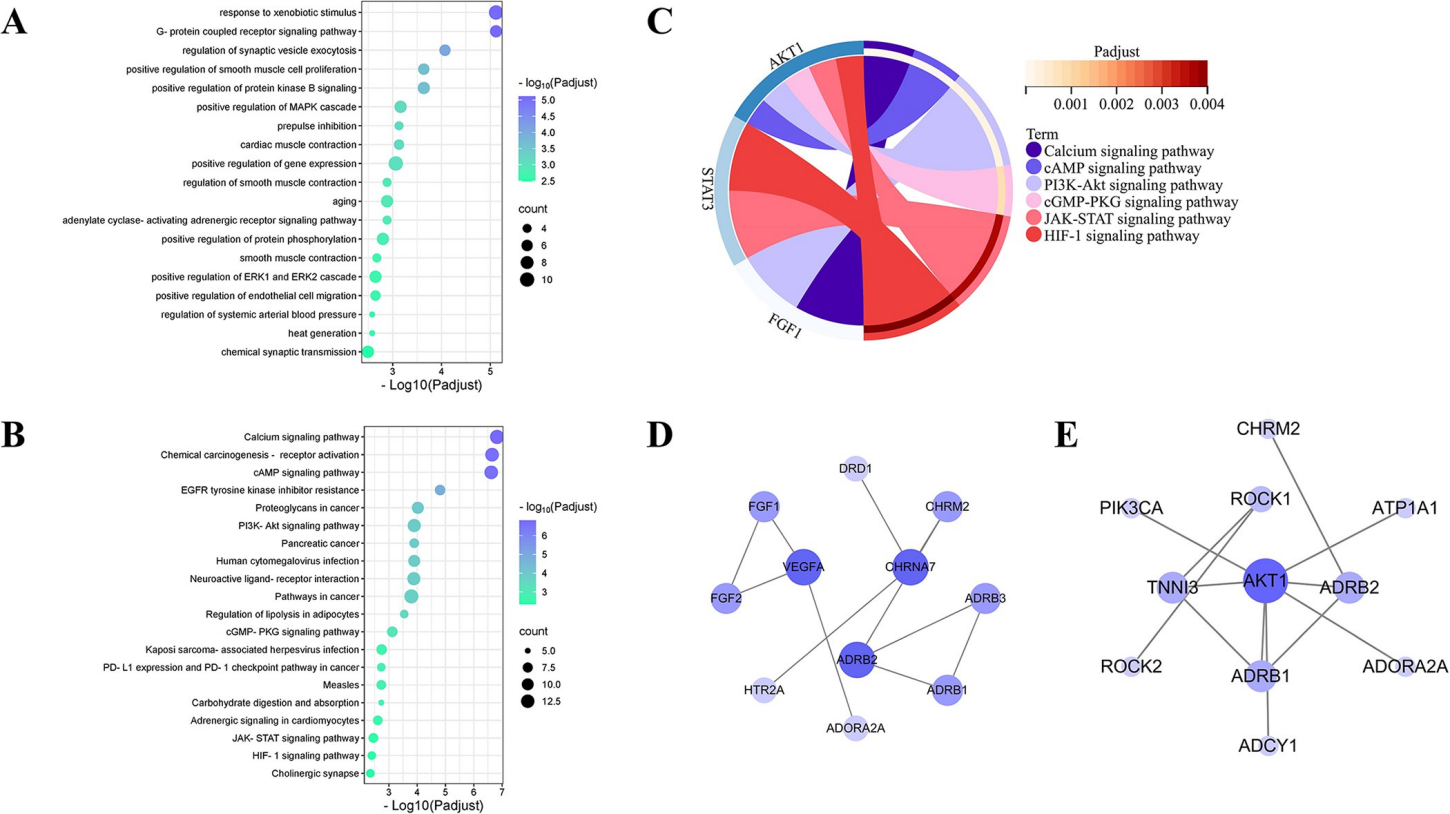
Enrichment analysis. (A, B) Enrichment analysis of BP (A) and KEGG (B) for SFI. (C) Potential therapeutic pathways and their associated core genes. (D, E) PPI network of calcium signaling pathway (D) and cAMP signaling pathway (E). The size and color of nodes were positively related to degree.

In the GO enrichment analysis, 64 of 166 items in the biological process (BP) analysis had a P adjust value <0.05, and the top 20 BP analyses were shown in Fig 3A. The BP enrichment results indicated that the therapeutic mechanism of SFI for SIMI involves multiple biological processes in the human body, including response to drugs, muscle contraction and conduction of multiple signaling pathways.

In the KEGG pathway analysis, 41 of the 87 pathways had a P adjust value <0.05, and the top 20 pathways were shown in Fig 3B. Among them, the calcium signaling pathway, cAMP signaling pathway, PI3K−Akt signaling pathway, cGMP-PKG signaling pathway, adrenergic signaling in cardiomyocytes, Jak−STAT signaling pathway and HIF-1 signaling pathway may be the potential therapeutic mechanisms of SFI for SIMI. Fig 3C demonstrated the core targets closely related to these potential therapeutic pathways, which can be seen that AKT1, FGF1, and STAT3 were all closely involved in, except for LGALS3, so AKT1, FGF1, and STAT3 were considered for further experimental verification.

In addition, since both KEGG and MCODE analyses were enriched in the calcium signaling pathway and cAMP signaling pathway, we exhibited the PPI network in which SFI targets were involved. Fig 3D was a PPI network of calcium signaling pathway with 11 nodes, 11 edges, which was based on 14 targets of SFI. Among them, CHRNA7 was the core target of this PPI network based on degree. And Fig 3E was a PPI network of cAMP signaling pathway with 11 nodes, 12 edges, which was based on 13 targets of SFI. Among them, AKT1 was the core target of this PPI network based on degree (S2 Table in S1 File).

### Molecular docking

We docked the core targets FGF1 (PDB ID: 1RG8), AKT1 (PDB ID: 1UNQ), STAT3 (PDB ID: 6NJS) and LGALS3 (PDB ID: 3ZSJ) with key active compounds, selecting the binding conformation with the lowest binding affinity. Research suggests that the lower the binding energy correlates with the better the ligand-receptor binding. Those with affinities lower than -5 kcal/mol have good binding ability, and those with affinities lower than -7 kcal/mol have strong binding ability [26].

**Fig 4:**
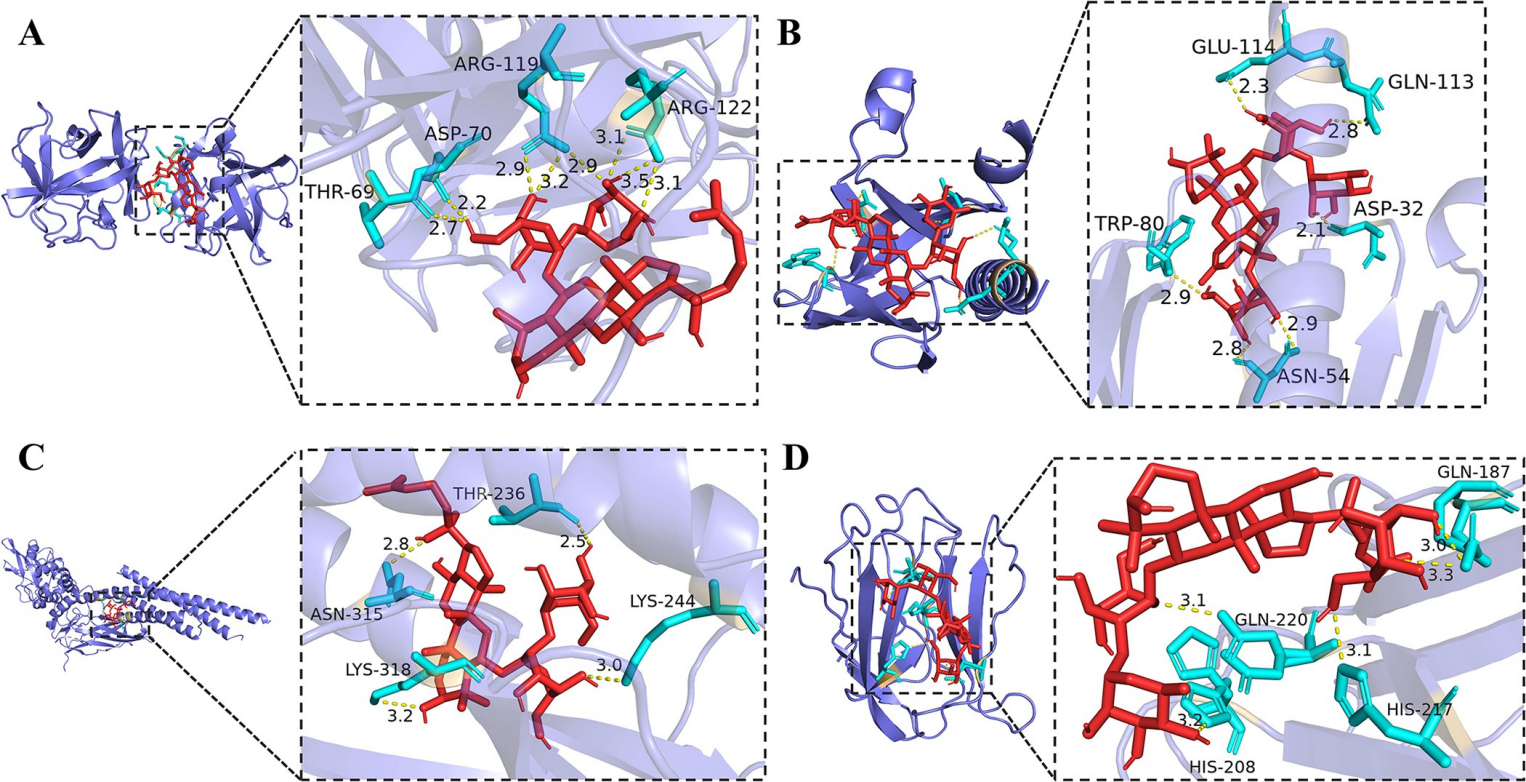
Molecular docking. (A) FGF1 and ginsenoside Rf, affinity = -8.6 kcal/mol. (B) AKT1 and ginsenoside Re, affinity = -9.4 kcal/mol. (C) STAT3 and ginsenoside Rf, affinity = -7.6 kcal/mol. (D) LGALS3 and ginsenoside Re, affinity = -7.8 kcal/mol.

**Fig 5:**
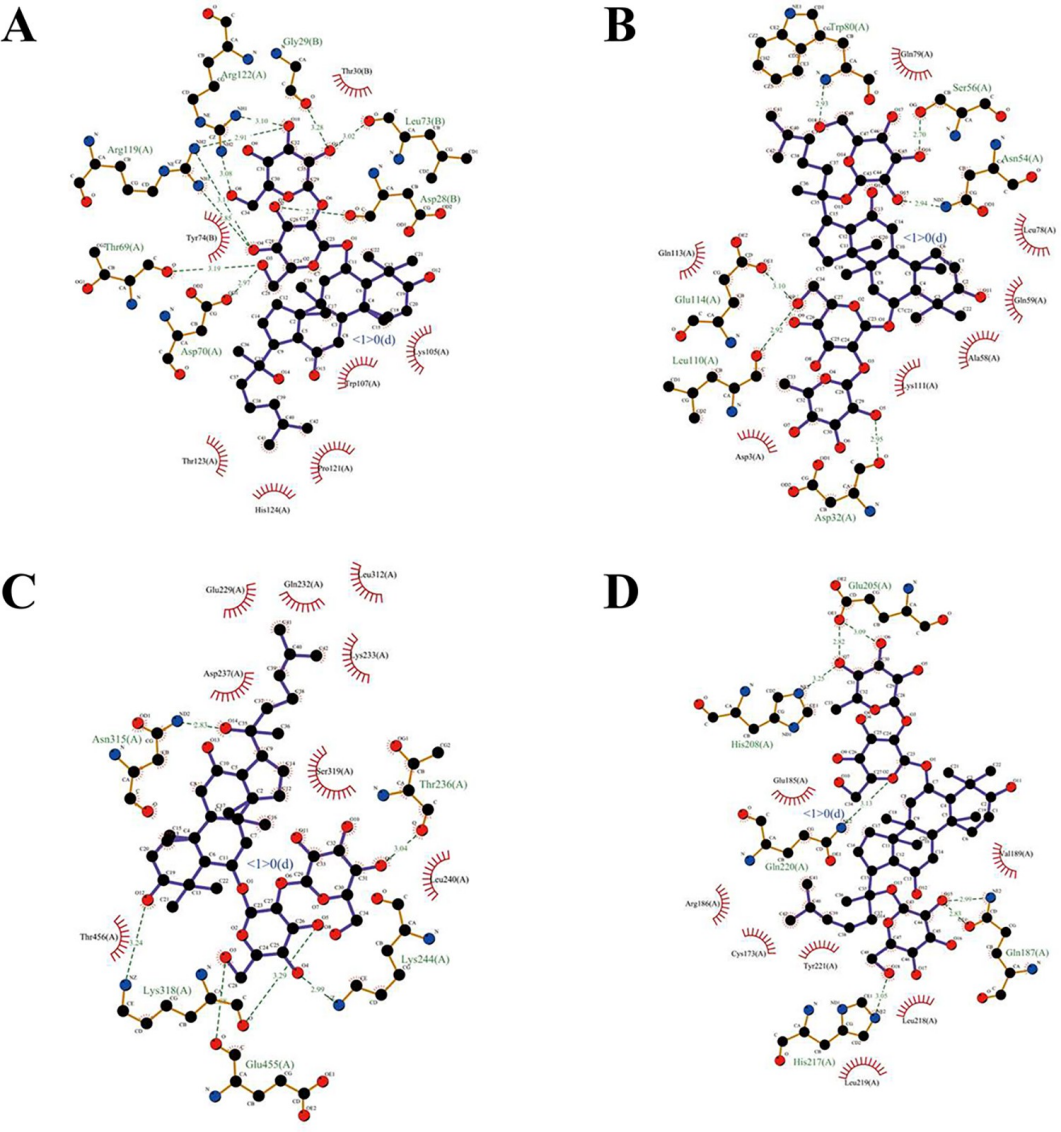
Interactions between compounds and residues. (A) FGF1 and ginsenoside Rf. (B) AKT1 and ginsenoside Re. (C) STAT3 and ginsenoside Rf. (D) LGALS3 and ginsenoside Re.

The docking results of AutoDock Vina 1.1.2 showed that almost all core targets showed strong binding ability to key active compounds (Table 2). We presented the molecular docking results for each target with the strongest binding ability (Fig 4), and demonstrated the interactions of compounds with residues (Fig 5). This further demonstrated the critical role of the core targets in the treatment of SIMI in SFI, and also proved that the key active compounds were important components of SFI, which have the potential to become drug candidates.

**Table 2:** Molecular docking results of key active compounds in SFI with core targets.

Compound	Binding energy (kcal/mol)			
	FGF1	AKT1	STAT3	LGALS3
Songorine	-8.3	-6.7	-7.2	-7.4
Ginsenoside Re	-8.2	-7.5	-7.1	-7.8
Ginsenoside Rf	-8.6	-6.7	-7.6	-7.3

### Protective effect of SFI in SIMI rats

**Fig 6:**
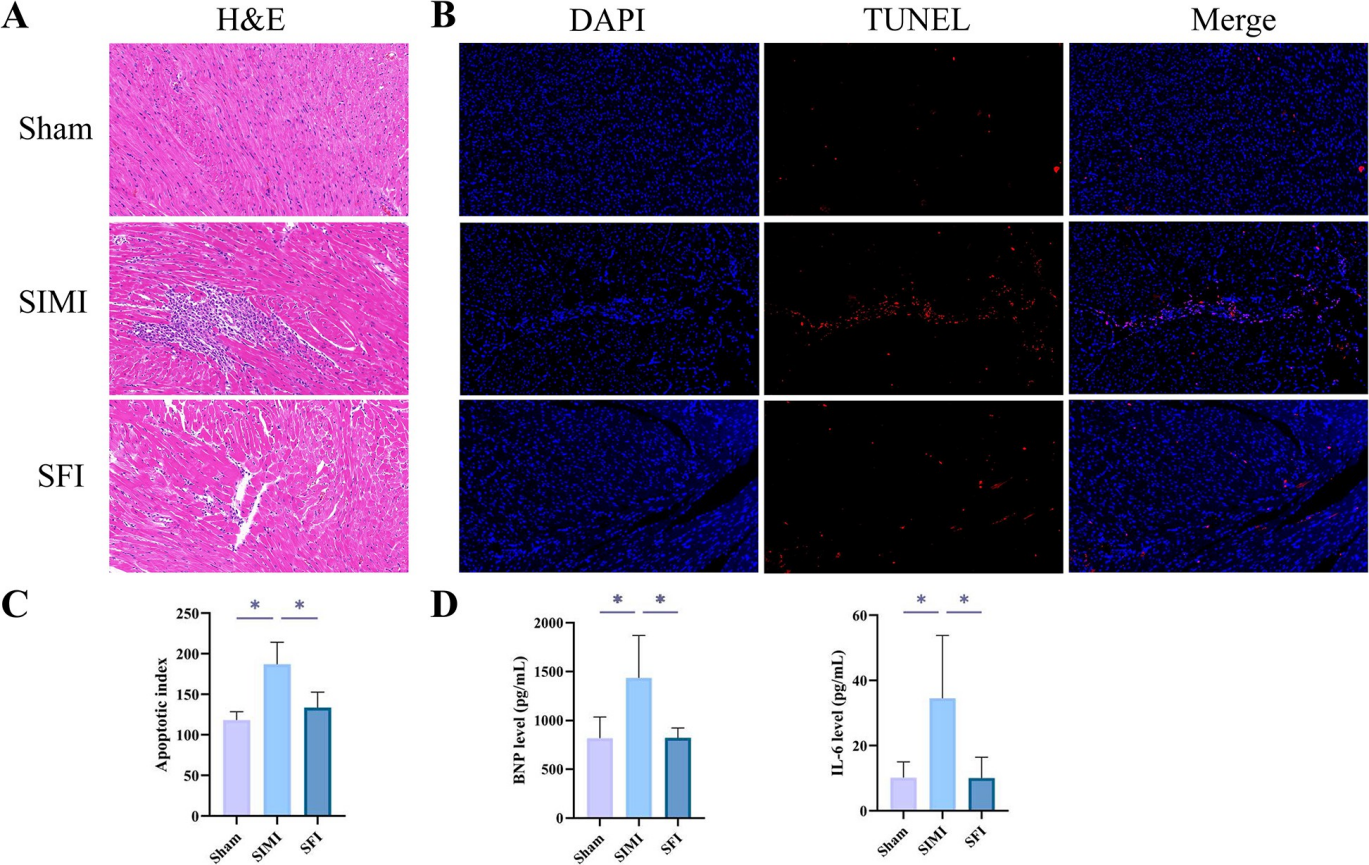
Protective effect of SFI in SIMI rats. (A) Representative images of H&E staining in rat myocardial tissues. (B, C) Representative images of TUNEL staining and its quantitative analysis in rat myocardial tissues. (D) Detection of changes in BNP and IL-6 levels in rats by ELISA. *p < 0.05.

In order to investigate the protective effect of SFI on SIMI, the rat model of SIMI was constructed by CLP surgery. H&E staining indicated that compared with the sham-operation group, the myocardial tissues of rats in the SIMI group exhibited disorganized cardiomyocyte arrangement, nuclear condensation and fragmentation, cytoplasmic disintegration, and lymphocyte infiltration. In contrast, the myocardial tissues of rats in the SFI group exhibited improved infiltration of lymphocytes, clearer nuclear structures and a more orderly cell arrangement (Fig 6A). TUNEL staining demonstrated that SFI improved the degree of cardiomyocyte apoptosis in myocardial tissues in SIMI (Fig 6B and 6C). These findings suggested that SFI can exert a cardioprotective effect on SIMI, reducing the inflammatory response, improving myocardial injury and inhibiting apoptosis of SIMI.

In addition, the ELISA assay found that SIMI treatment increased the levels of BNP and IL-6 in myocardial tissues, while SFI significantly reduced the levels of BNP and IL-6 (Fig 6D). This further confirmed that SFI can effectively improve SIMI and reduce the inflammatory response.

### Treatment of SIMI with SFI through FGF1/AKT1/STAT3

**Fig 7:**
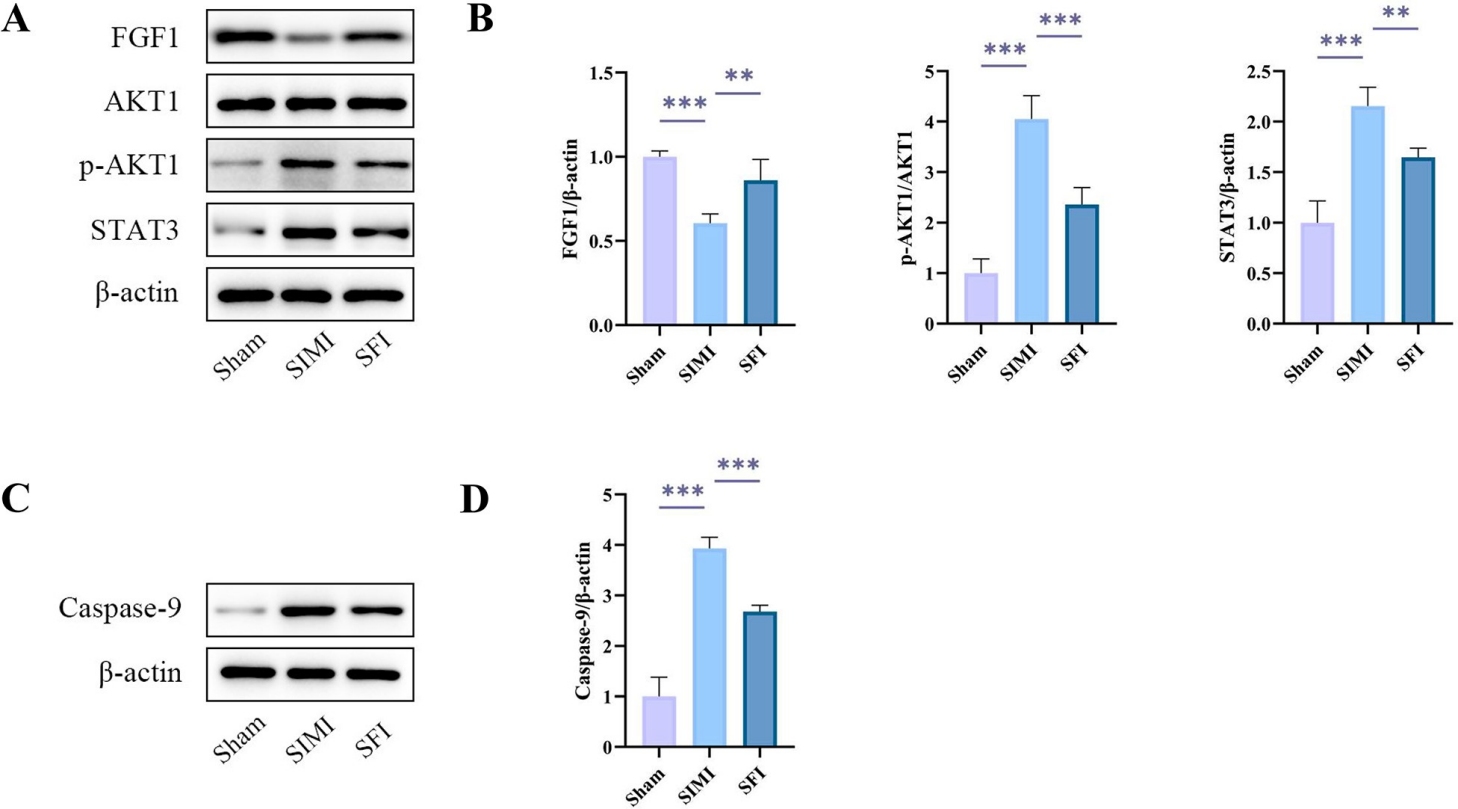
The expression levels of core target proteins through western blot. (A, B) Western bot analysis of FGF1, total AKT1, p-AKT1, and STAT3 in each group (n = 4). (C, D) Western bot analysis of caspase-9 in each group (n = 4). p-AKT1, phospho-AKT1; *p < 0.05, **p < 0.01, ***p < 0.001.

To further verify whether FGF1, AKT1 and STAT3 were the key targets of SFI for the treatment of AMI, we used western blot to detect the expression levels of related proteins in SIMI (Fig 7). We observed that SIMI treatment reduced the expression of FGF1, while SFI treatment significantly upregulated the expression of FGF1. Moreover, SIMI treatment upregulated the expression of STAT3, whereas SFI treatment significantly reduced the expression of STAT3. Interestingly, there was no significant difference in the total protein levels of AKT1, but a significant difference was observed in the active form of AKT1, p-AKT1. SIMI treatment upregulated the expression of p-AKT1, and SFI treatment significantly reduced the expression of p-AKT1 (Fig 7A and 7B).

In addition, we detected the expression level of apoptotic protein by western blot (Fig 7C). SIMI treatment upregulated the expression of caspase-9, whereas SFI treatment significantly reduced the expression of caspase-9 (Fig 7D). It can be seen that SFI can effectively inhibit apoptosis of the myocardium.

## Discussion

SIMI is a common complication of sepsis and a key cause of adverse outcomes in sepsis [27]. A study found that about 60% of patients with sepsis had myocardial injury, which was independently associated with increased early mortality [28]. SFI has been proved to be effective in treating sepsis [7], inhibiting cardiomyocyte apoptosis and preventing SIMI [11, 12]. Our study showed that songorine, ginsenoside Re and ginsenoside Rf were key compounds in the SFI treatment of SIMI. LGALS3, STAT3, FGF1 and AKT1 were identified as the core targets of SFI. Our experiments further demonstrated that SFI effectively reduced the inflammatory response, inhibited apoptosis and improved SIMI by reducing the expression of STAT3 and p-AKT1 while enhancing the expression of FGF1. In addition, the calcium signaling pathway, cAMP signaling pathway, PI3K-Akt signaling pathway, cGMP-PKG signaling pathway, adrenergic signaling in cardiomyocytes, Jak-STAT signaling pathway and HIF-1 signaling pathway may be the potential therapeutic mechanisms of SFI for SIMI.

According to the MCODE analysis, we identified LGALS3, STAT3, FGF1 and AKT1 as the core targets of SFI in the treatment of SIMI. LGALS3 is a β-galactose and participates in cell growth, apoptosis, inflammatory reaction, angiogenesis and other cell activities, which is considered as a novel inflammatory factor [29, 30]. The study found that elevated level of LGALS3 can be observed in almost all cardiovascular diseases and plays an important role in clinical prognosis [29]. The expression of LGALS3 promotes myocardial ischemia-reperfusion injury [31], induces inflammation and exacerbates sepsis [32].

AKT1 and STAT3 have been demonstrated as key genes in pyroptosis in sepsis, with high expression levels observed in lung tissue and lymphocytes [33]. STAT3 is a signaling transcriptional factor intimately involved in various biological processes, including anti/pro-inflammatory responses [34]. STAT3 was found to exacerbate the septic response by inducing macrophages activation [35], and was involved in the pathological process of SIMI [36]. The downregulation of STAT3 levels in cardiac myocytes can protect the heart by reducing inflammation and cell death in sepsis [36]. AKT can be intimately involved in apoptosis, metabolism and other life processes through phosphorylation, improving sepsis survival by participating in apoptosis of lymphocytes [37]. AKT1, one of the isoforms of AKT, has been confirmed to be closely associated with acute inflammation [38] and is intimately involved in cardiac injury and remodeling [39]. The activation of AKT1 can promote apoptosis and inflammatory responses, accelerating the progression of sepsis [40].

FGF1, a member of the fibroblast growth factor family, has been shown to provide protective effects against injurious stimuli in a variety of disease models, and exhibits anti-inflammatory properties, inhibits apoptosis, and resist oxidation stress [41, 42]. The activation of FGF1 was found to improve oxidative stress, inhibit cardiomyocyte apoptosis, and protect the heart [41, 43].

The molecular docking result showed that the core targets of SIMI docked well with the key compounds of SFI, which demonstrated the key role of the core targets in the treatment of SIMI in SFI. And the experiment further demonstrated that SFI could effectively reduce the inflammatory response, inhibit apoptosis and improve SIMI by reducing the expression of STAT3 and p-AKT1 and enhancing the expression of FGF1.

SFI is derived from the TCM formulation, which has been shown to be effective in a variety of critical illnesses such as sepsis [13], acute heart failure [44] and acute myocardial infarction [45], improving patient prognosis and increasing survival rates. Although studies have confirmed that SFI can effectively inhibit apoptosis in cardiomyocytes, reduce the inflammatory response and improve SIMI [10–12], the exact mechanism of action is unclear and clinical application is limited. Our study showed that songorine, ginsenoside Re and ginsenoside Rf are key compounds in the SFI treatment of SIMI. And SFI can improve SIMI by decreasing STAT3 expression and p-AKT1, enhancing FGF1 expression, and inhibiting cardiomyocyte apoptosis and reducing the inflammatory response.

However, some limitations should be acknowledged. First, although our findings were consistent with previous studies [10–12], the lack of a positive treatment control affects the accuracy of the study and should be included in future work to further validate and extend the implications of our findings. Second, our study was analyzed and validated mainly by quantitative data, and the lack of representative rat images was a shortcoming of this study, which stems from our efforts to minimize animal discomfort and suffering.

## Conclusions

This study explores the therapeutic mechanism of SFI for SIMI based on network pharmacology, and validates it through molecular docking and experiment. The potential mechanisms of SFI for SIMI include reduced STAT3 and p-AKT1 expression, enhanced FGF1 expression, inhibited cardiomyocyte apoptosis and reduced inflammatory response.

## Supporting information

S1 File (PDF)

S1 Raw data (ZIP)
