# Supplementary material for: Exploration and verification of the therapeutic mechanism of shenfu injection in sepsis-induced myocardial injury
Source: PLoS One. 2025 Jan 17;20(1):e0317738. doi: 10.1371/journal.pone.0317738 (PMC11741597; doi:10.1371/journal.pone.0317738)
Supplement: S1 File — (PDF) [file pone.0317738.s001.pdf]

S1 Table. The potential therapeutic targets of SFI and their corresponding active compounds.

Compound	Target
Benzoylhypaconine	BCHE, PIK3CA, MTOR, SLC6A3, GBA, UGCG, CHRNA7, ADCY1
Fuziline	GBA, MGAM, UGCG, GAA
Ginsenoside Re	LGALS3, STAT3, IL2, VEGFA, FGF2, HSP90AA1, BCL2L1, PTAFR, FGF1, HPSE, ATP1A1, HSD11B2
Ginsenoside Rf	STAT3, VEGFA, FGF2, IL2, FGF1, HPSE, PTAFR, F7, SLC5A1, HSD11B2
Hypaconine	GBA, UGCG, MGAM, GAA, PNP, OGA
Karacoline	ADRB2, DRD1, CHRM2, GBA, FUCA1, PNP, BACE1, ADRB1, ADRB3
Mesaconine	GBA, GAA, UGCG, MGAM, ADA, OGA, PNP
Neoline	GBA, UGCG, GAA, MGAM, CHRM2, BACE1
Songorine	AKT1, DRD1, ADRB2, BCHE, TLR4, CYP2D6, SLC6A3, HTR1A, HTR2A, ABCB1, PARP1, CHRNA3, ADRB1, ADRB3, HTR3A, JAK1, ROCK2, ROCK1, CHRNA3, TNNT2, TNNI3, CCR1, HRH1, ADORA2A, ROCK2, KDM1A
Talatisamine	CHRM2, GBA, BACE1, PNP, UGCG, GAA

S2 Table. PPI networks constructed by SFI targets.

PPI	Name	Degree
Calcium signaling pathway	CHRNA7	4
	ADRB2	3
	VEGFA	3
	FGF1	2
	FGF2	2
	ADRB3	2
	ADRB1	2
	ADORA2A	1
	CHRM2	1
	DRD1	1
	HTR2A	1
cAMP signaling pathway	AKT1	7
	ADRB1	3
	ADRB2	3
	TNNI3	3
	ROCK1	2
	ADCY1	1
	ADORA2A	1
	CHRM2	1
	PIK3CA	1
	ATP1A1	1
	ROCK2	1
