# Supplementary figures and images for: Exploration and verification of the therapeutic mechanism of shenfu injection in sepsis-induced myocardial injury
Source: PLoS One. 2025 Jan 17;20(1):e0317738. doi: 10.1371/journal.pone.0317738 (PMC11741597; doi:10.1371/journal.pone.0317738)

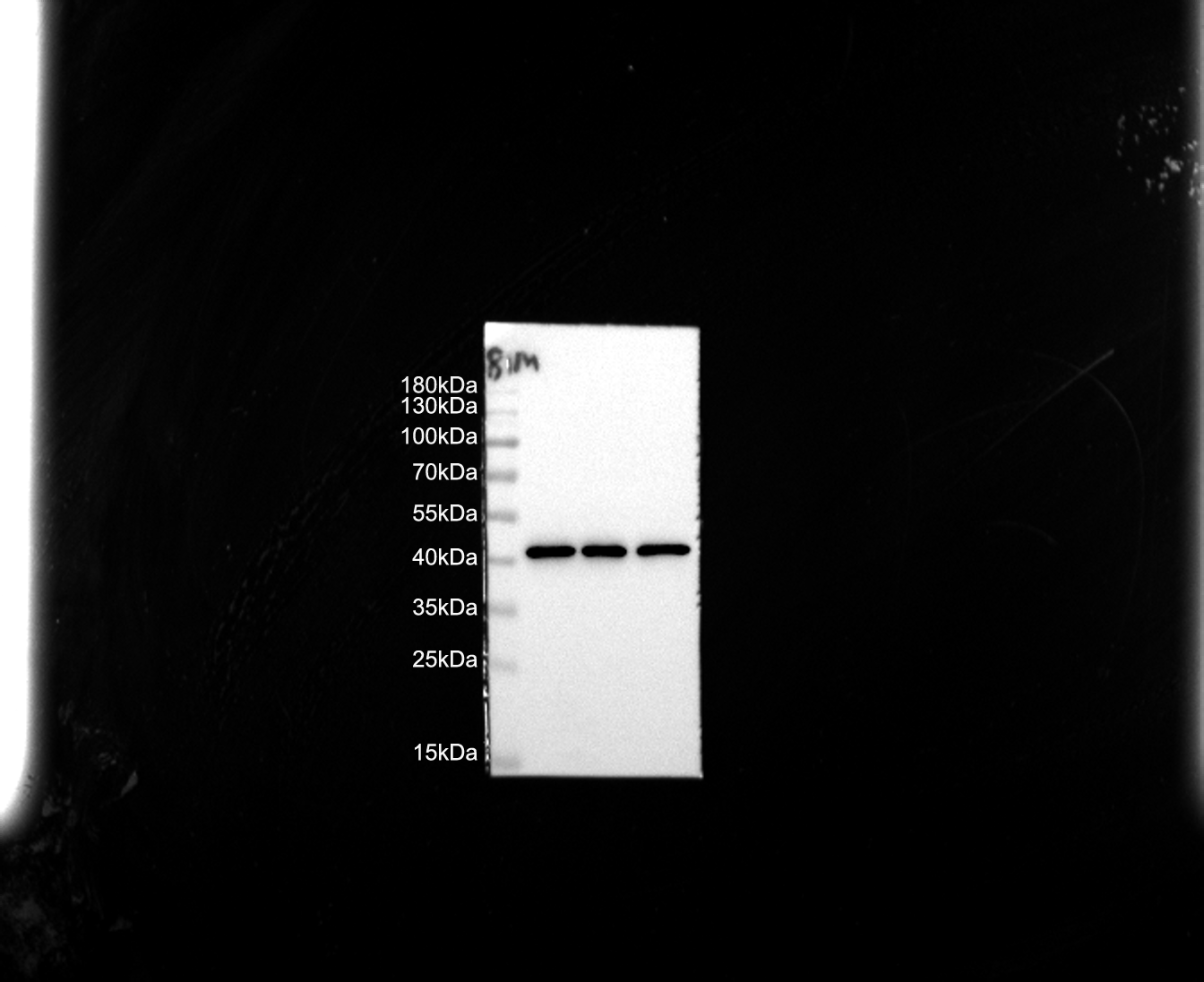

Supplement: S1 Raw data — (ZIP) [file pone.0317738.s002.zip › S1 Raw data/repeat 1/ACTIN..Tif]

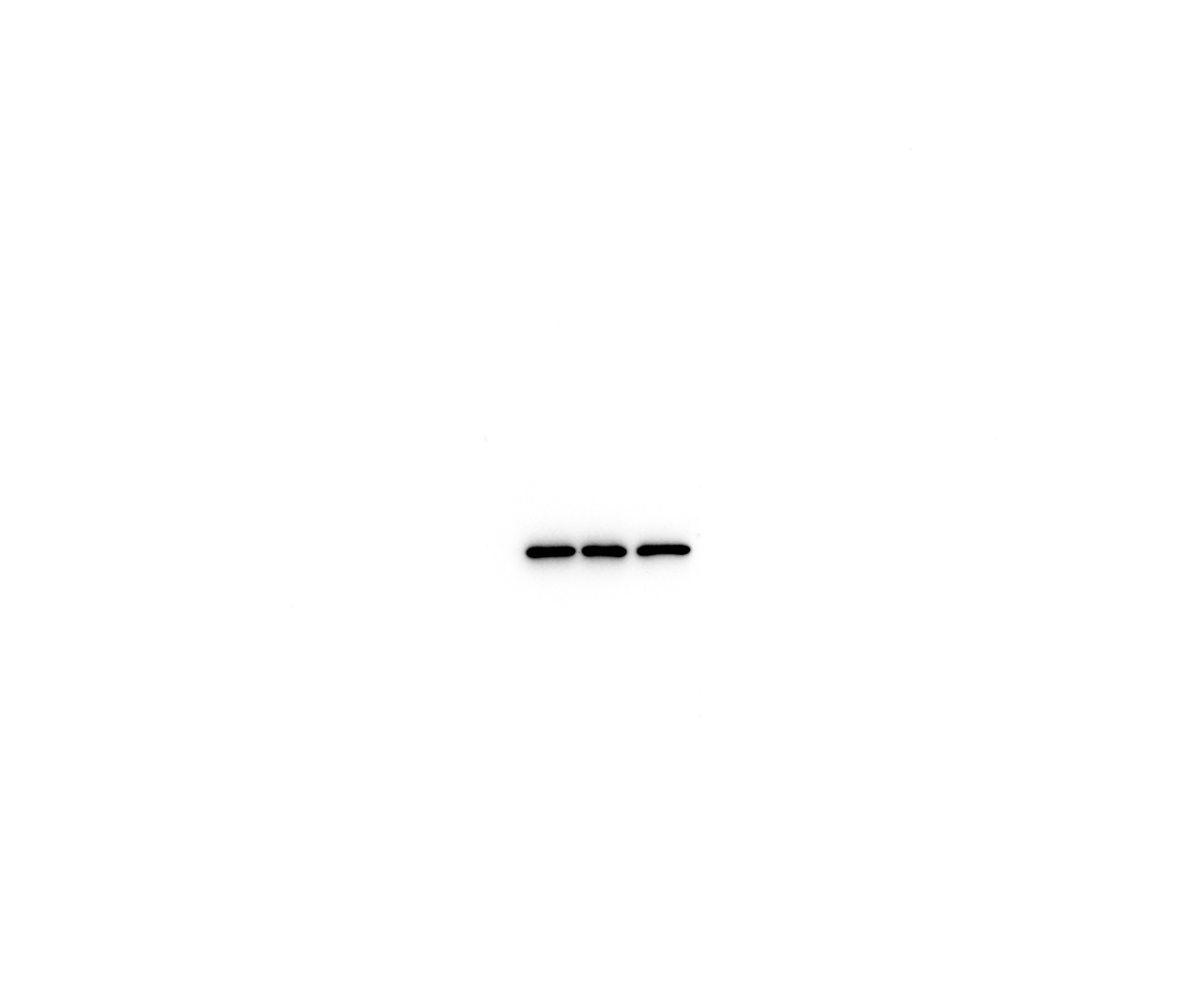

Supplement: S1 Raw data — (ZIP) [file pone.0317738.s002.zip › S1 Raw data/repeat 1/ACTIN.Tif]

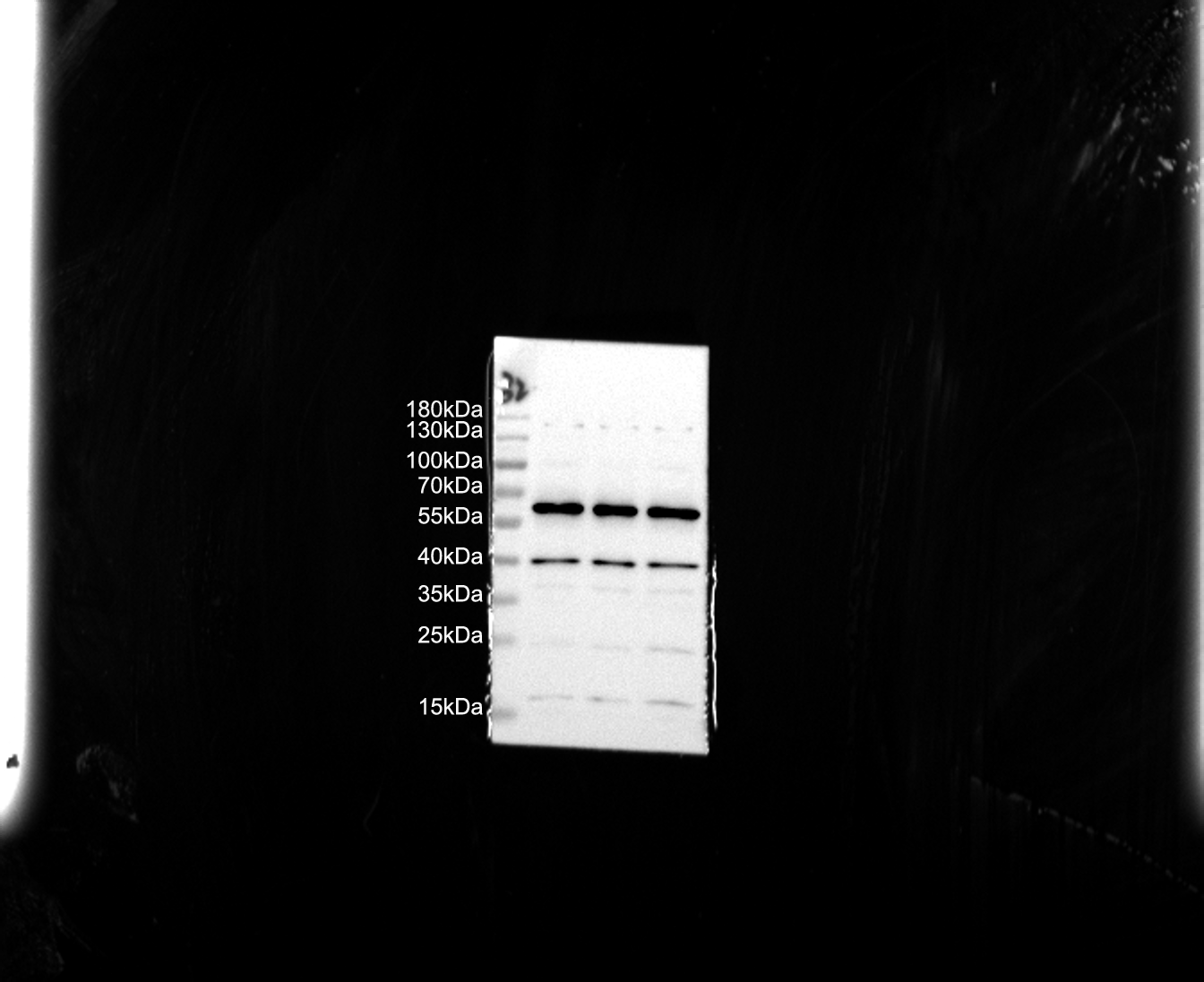

Supplement: S1 Raw data — (ZIP) [file pone.0317738.s002.zip › S1 Raw data/repeat 1/AKT..Tif]

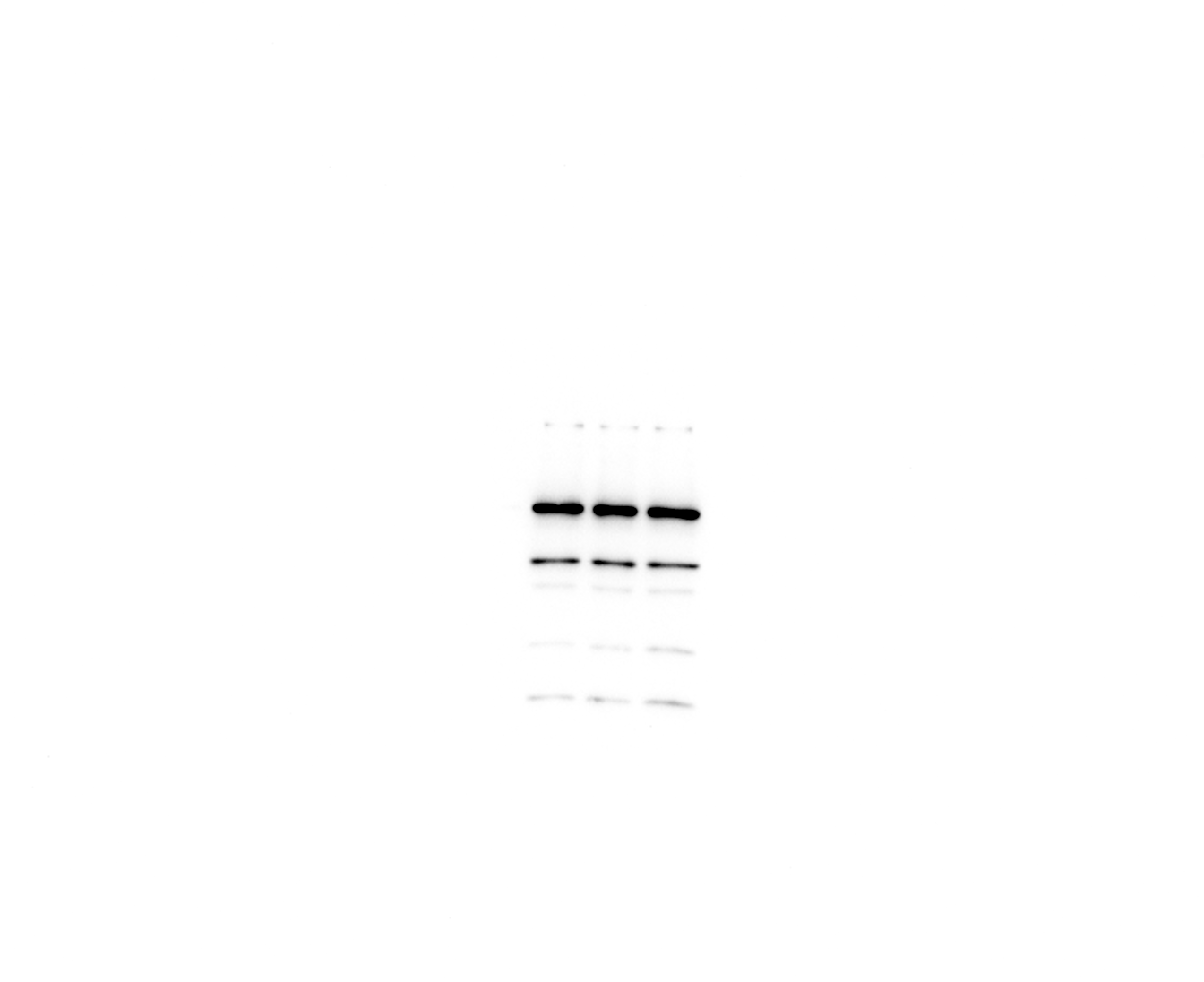

Supplement: S1 Raw data — (ZIP) [file pone.0317738.s002.zip › S1 Raw data/repeat 1/AKT.Tif]

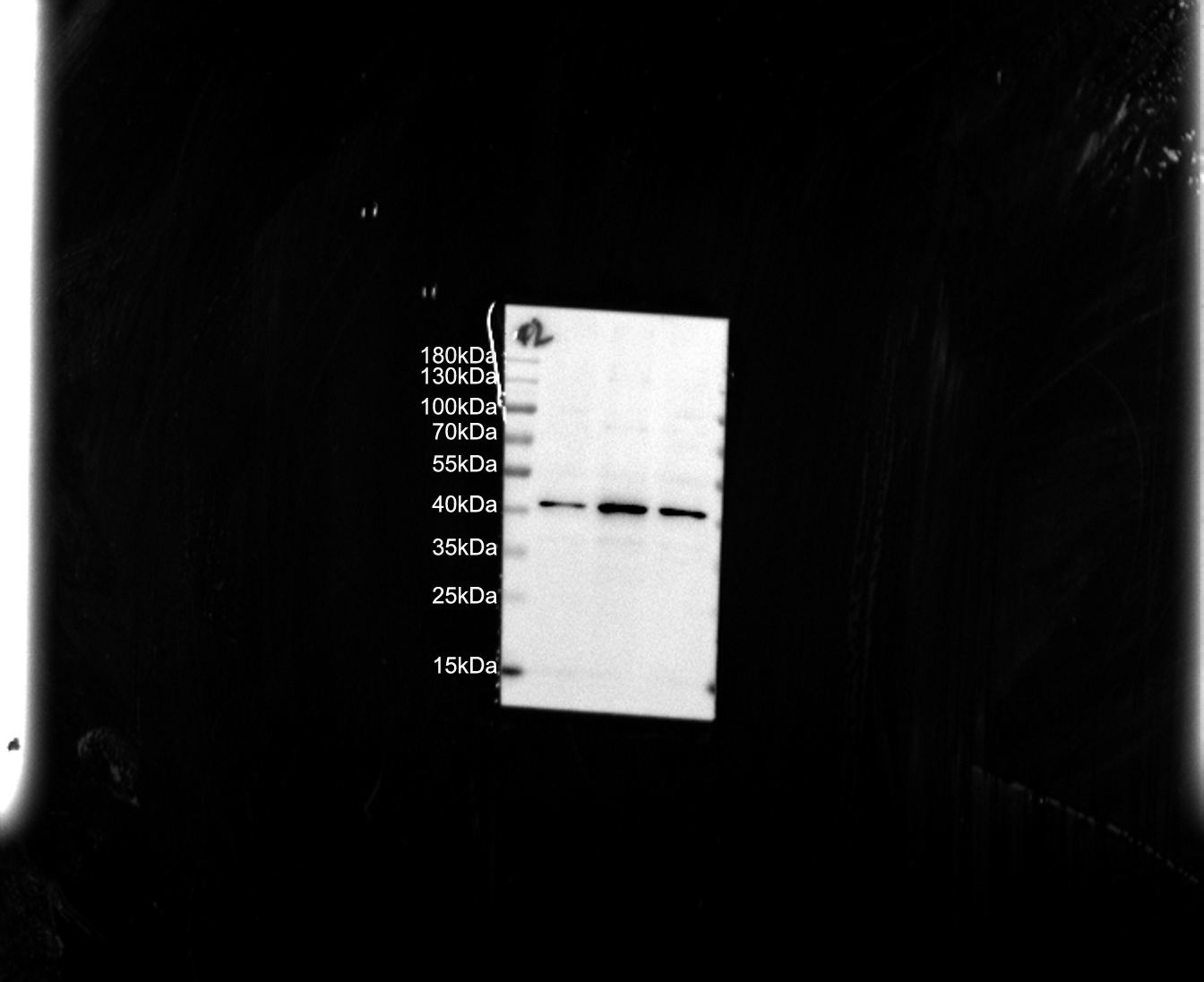

Supplement: S1 Raw data — (ZIP) [file pone.0317738.s002.zip › S1 Raw data/repeat 1/CAS9..Tif]

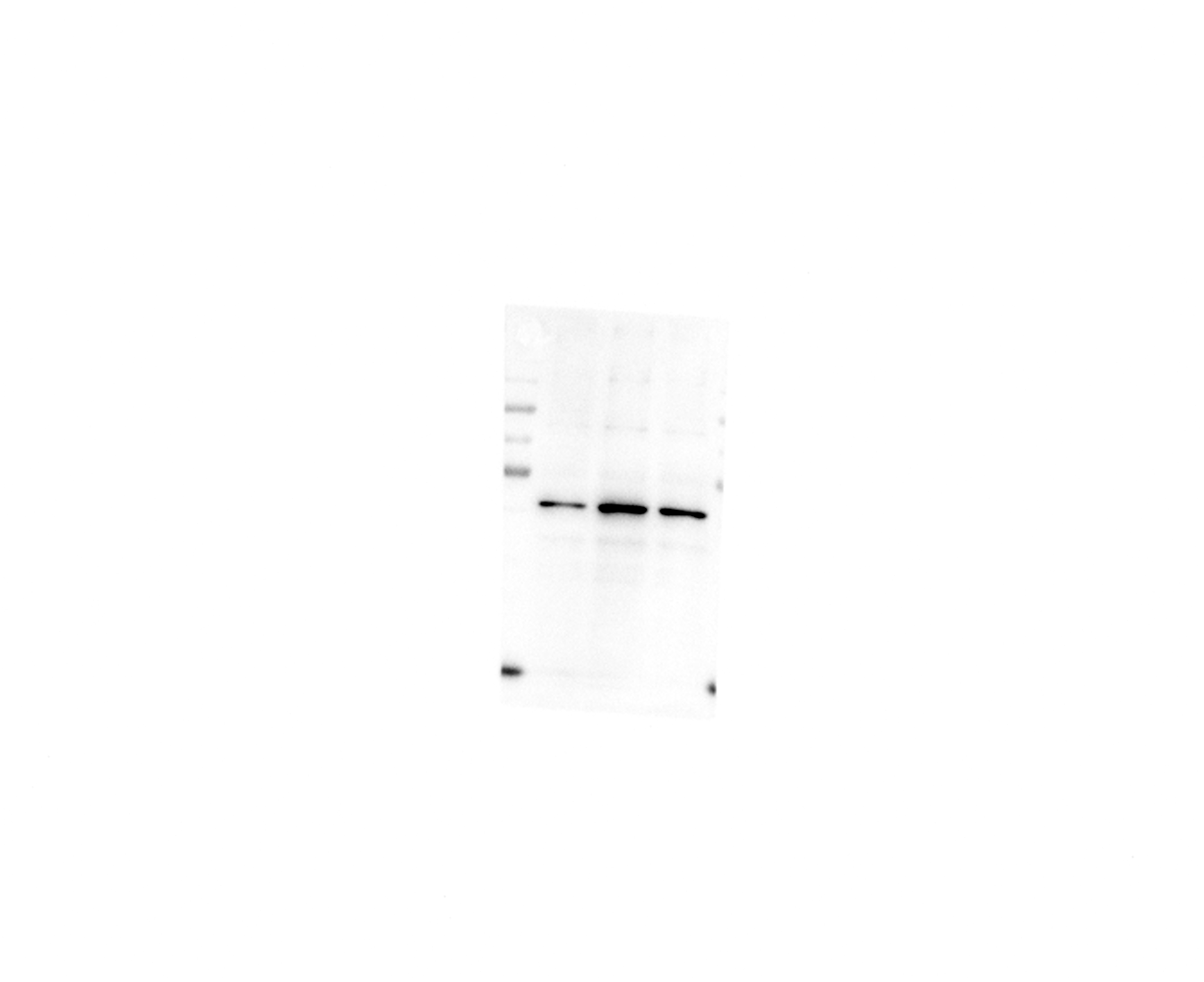

Supplement: S1 Raw data — (ZIP) [file pone.0317738.s002.zip › S1 Raw data/repeat 1/CAS9.Tif]

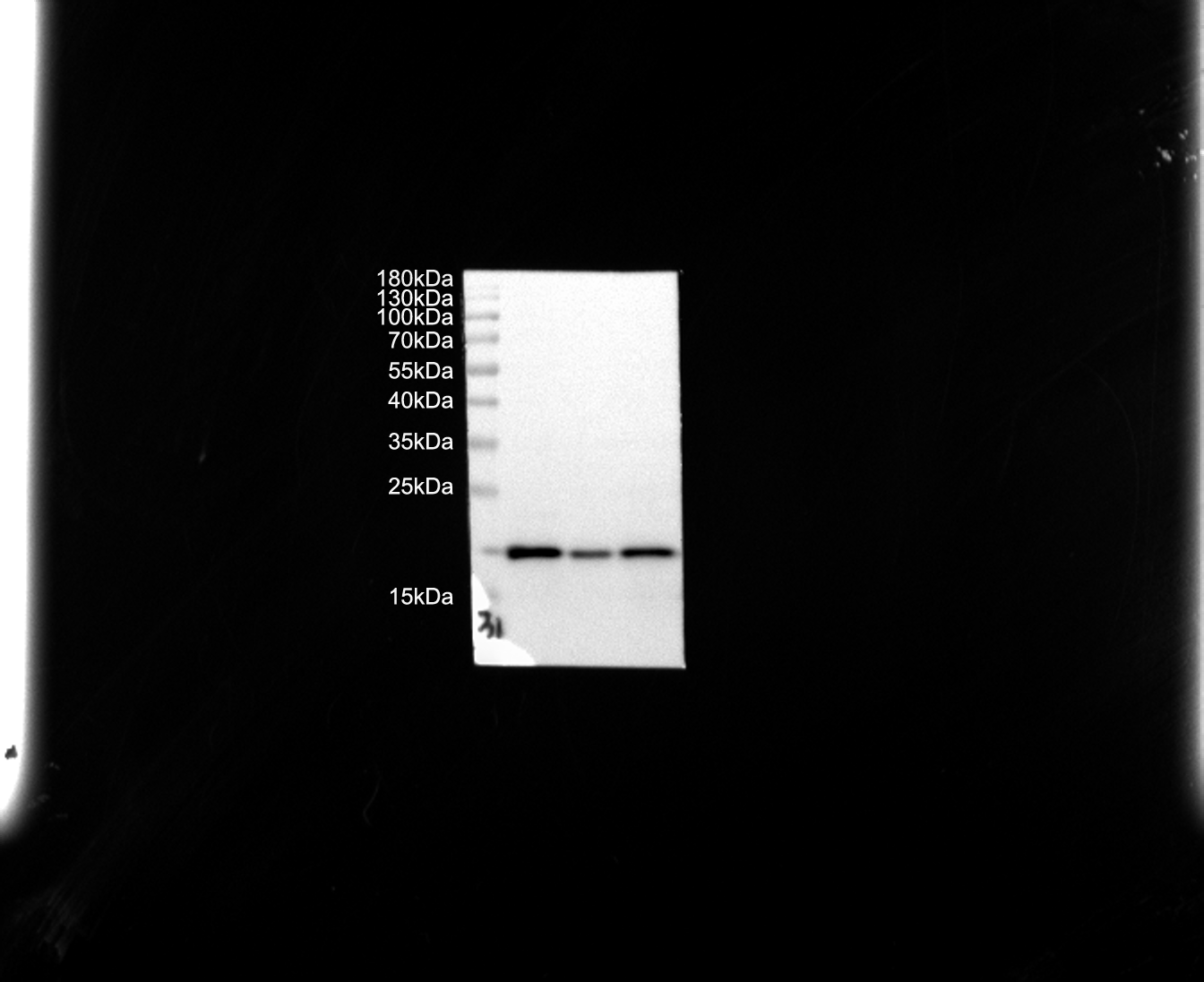

Supplement: S1 Raw data — (ZIP) [file pone.0317738.s002.zip › S1 Raw data/repeat 1/FGF1..Tif]

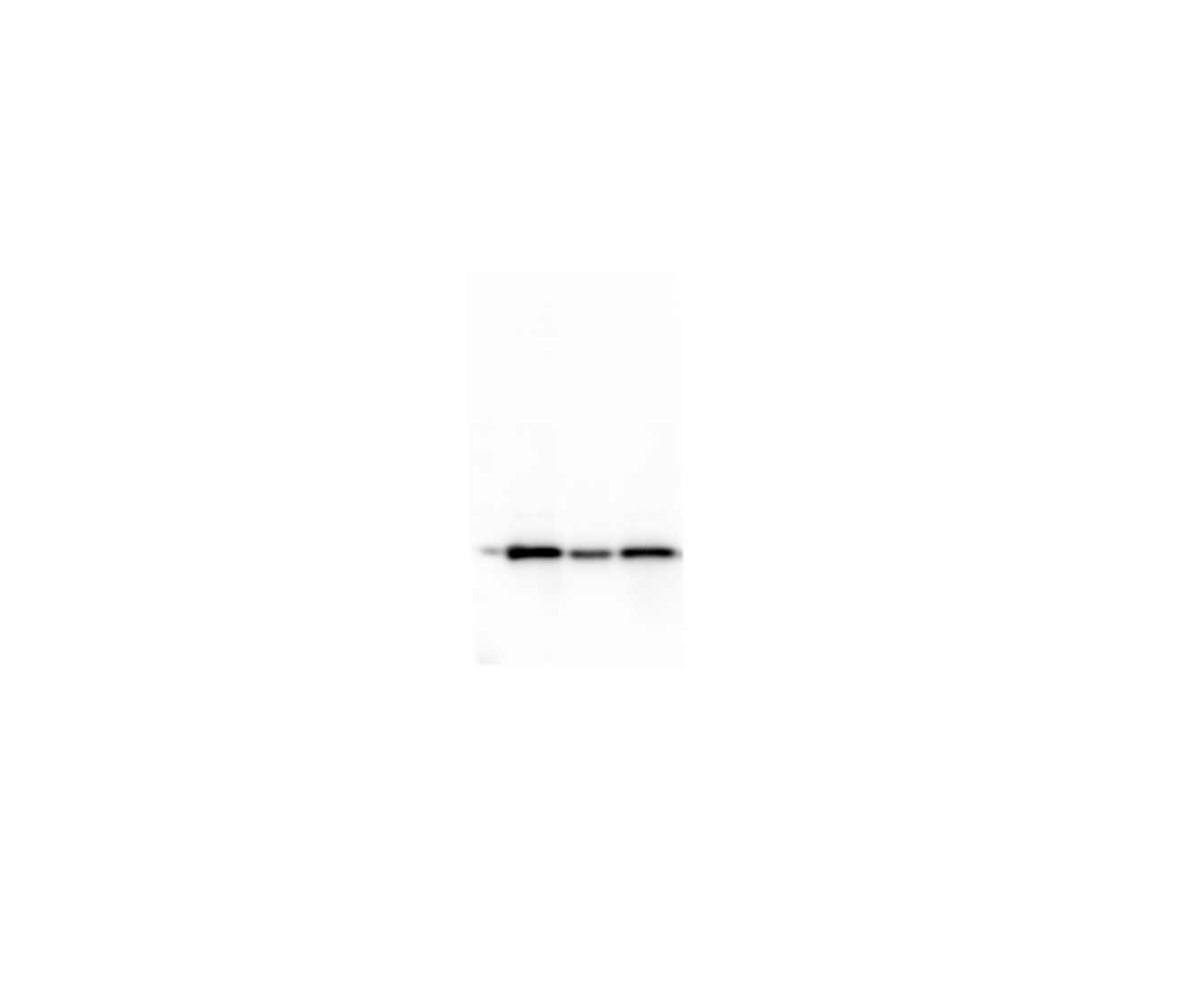

Supplement: S1 Raw data — (ZIP) [file pone.0317738.s002.zip › S1 Raw data/repeat 1/FGF1.Tif]

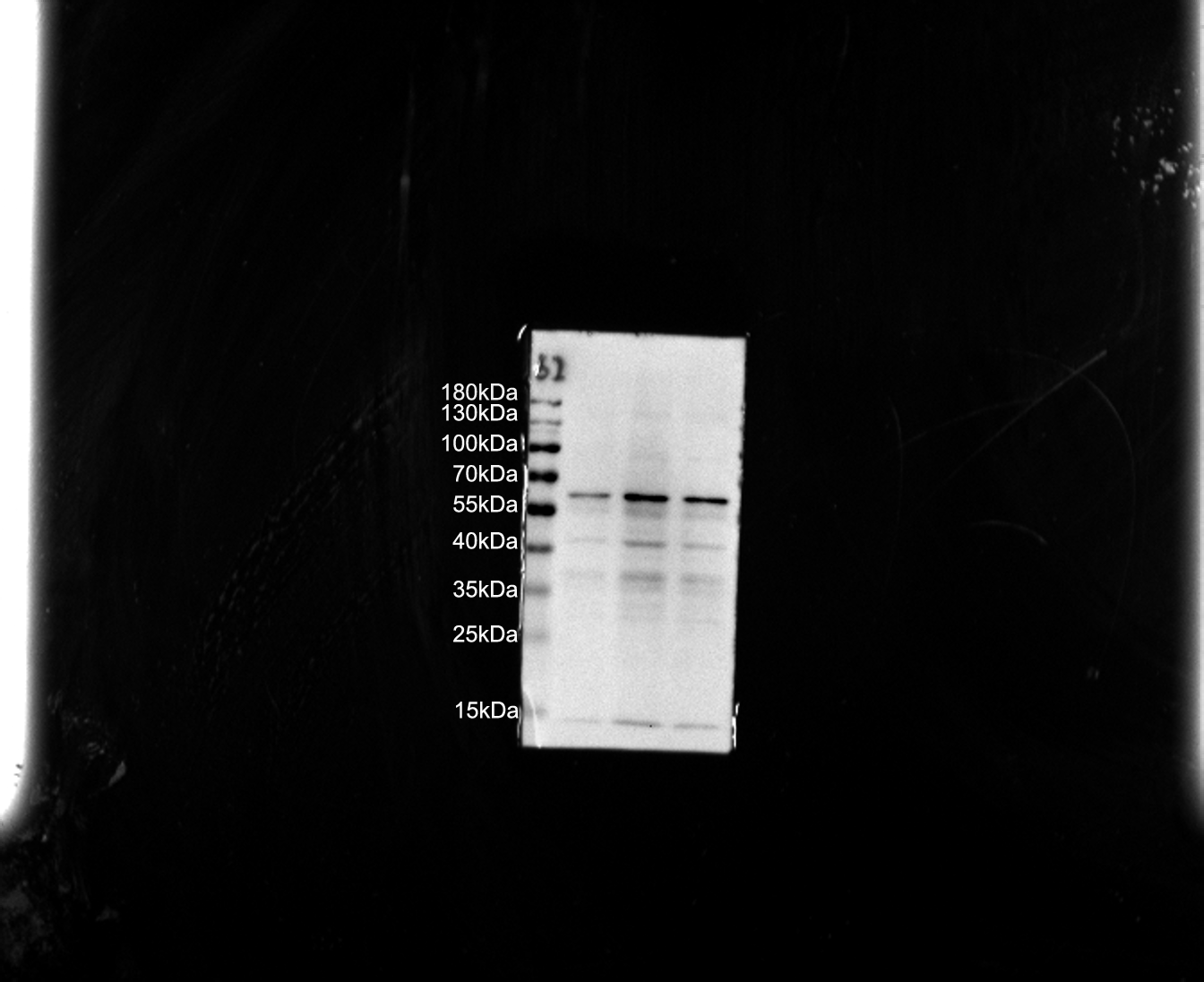

Supplement: S1 Raw data — (ZIP) [file pone.0317738.s002.zip › S1 Raw data/repeat 1/P-AKT..Tif]

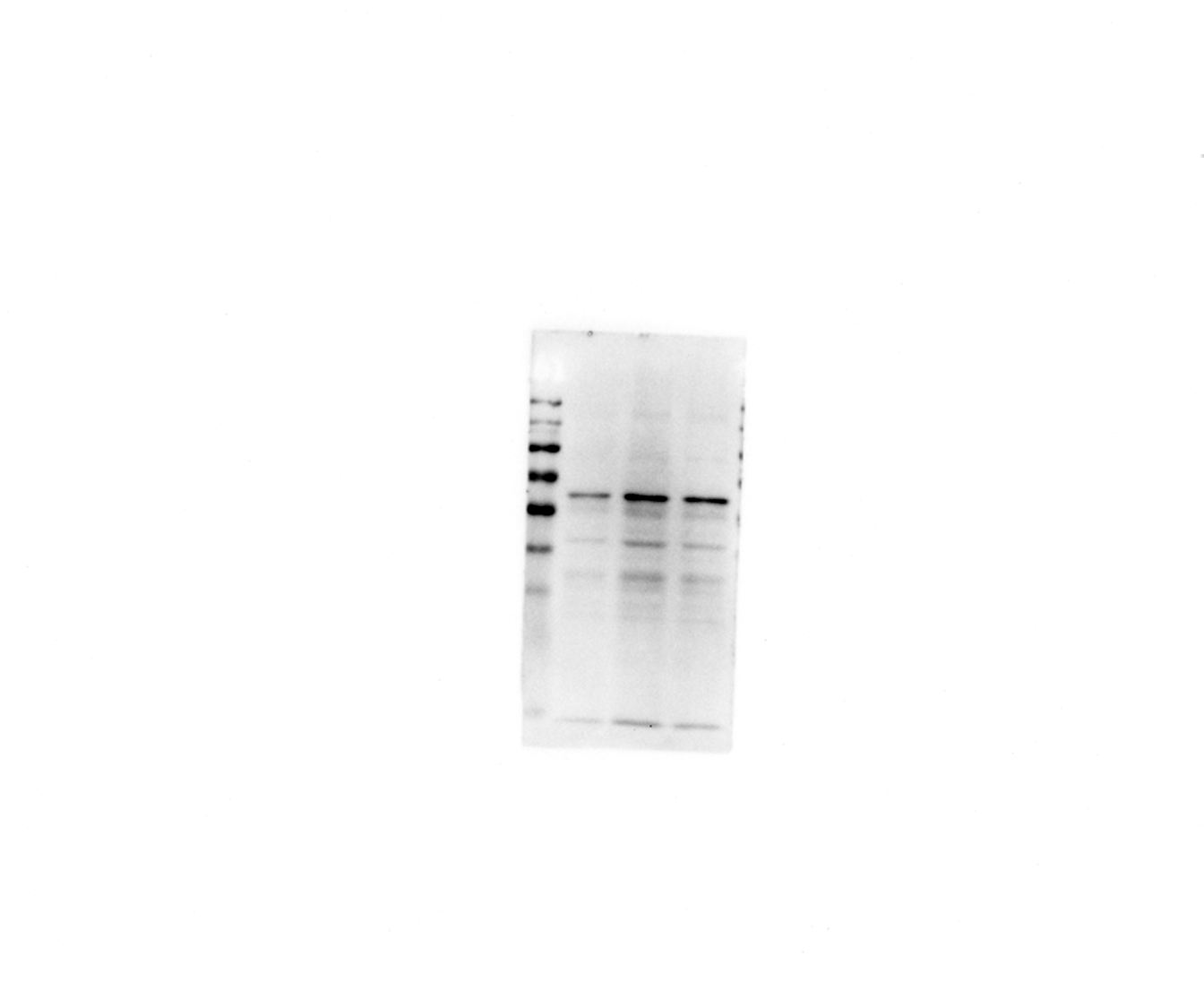

Supplement: S1 Raw data — (ZIP) [file pone.0317738.s002.zip › S1 Raw data/repeat 1/P-AKT.Tif]

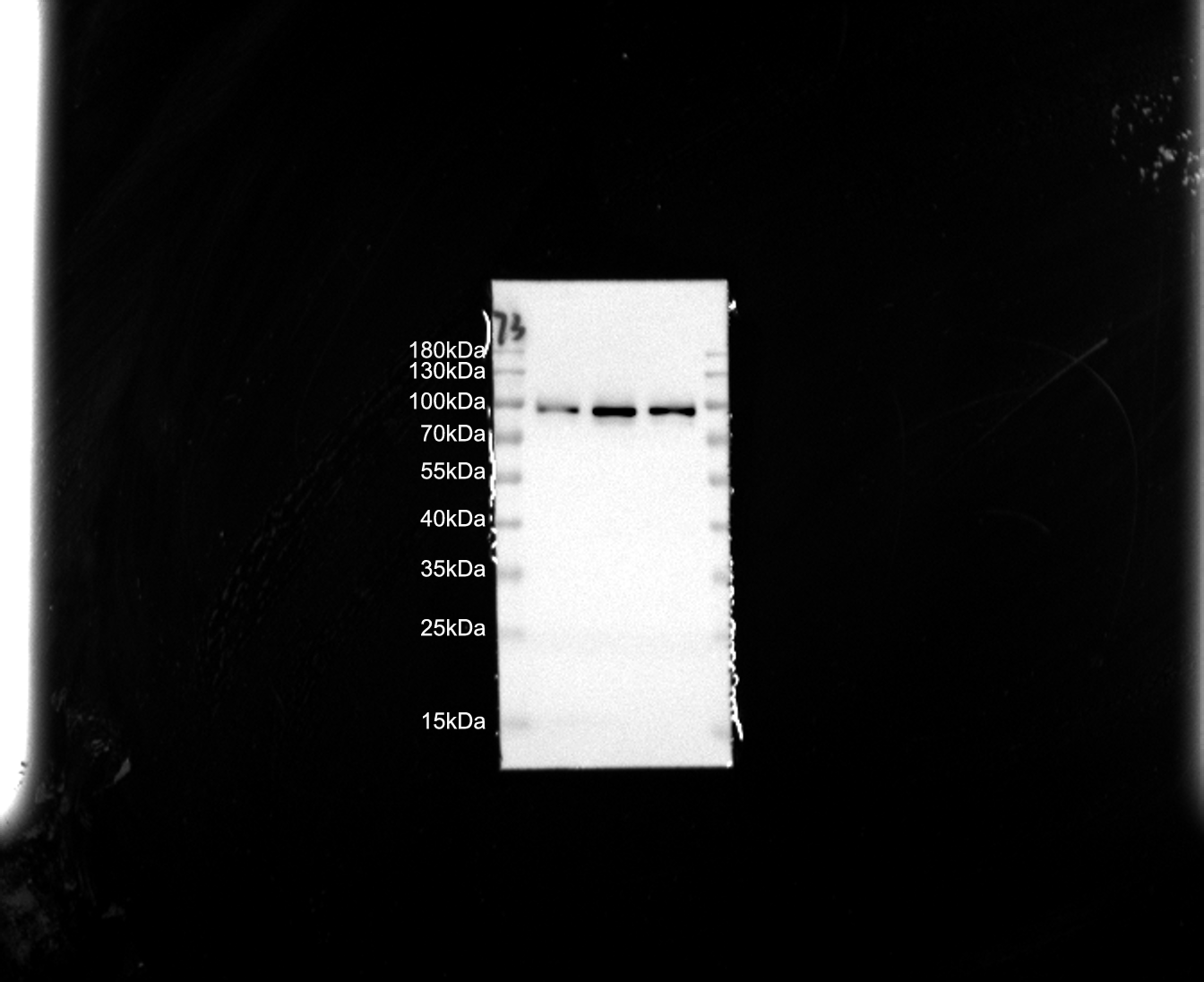

Supplement: S1 Raw data — (ZIP) [file pone.0317738.s002.zip › S1 Raw data/repeat 1/STAT3..Tif]

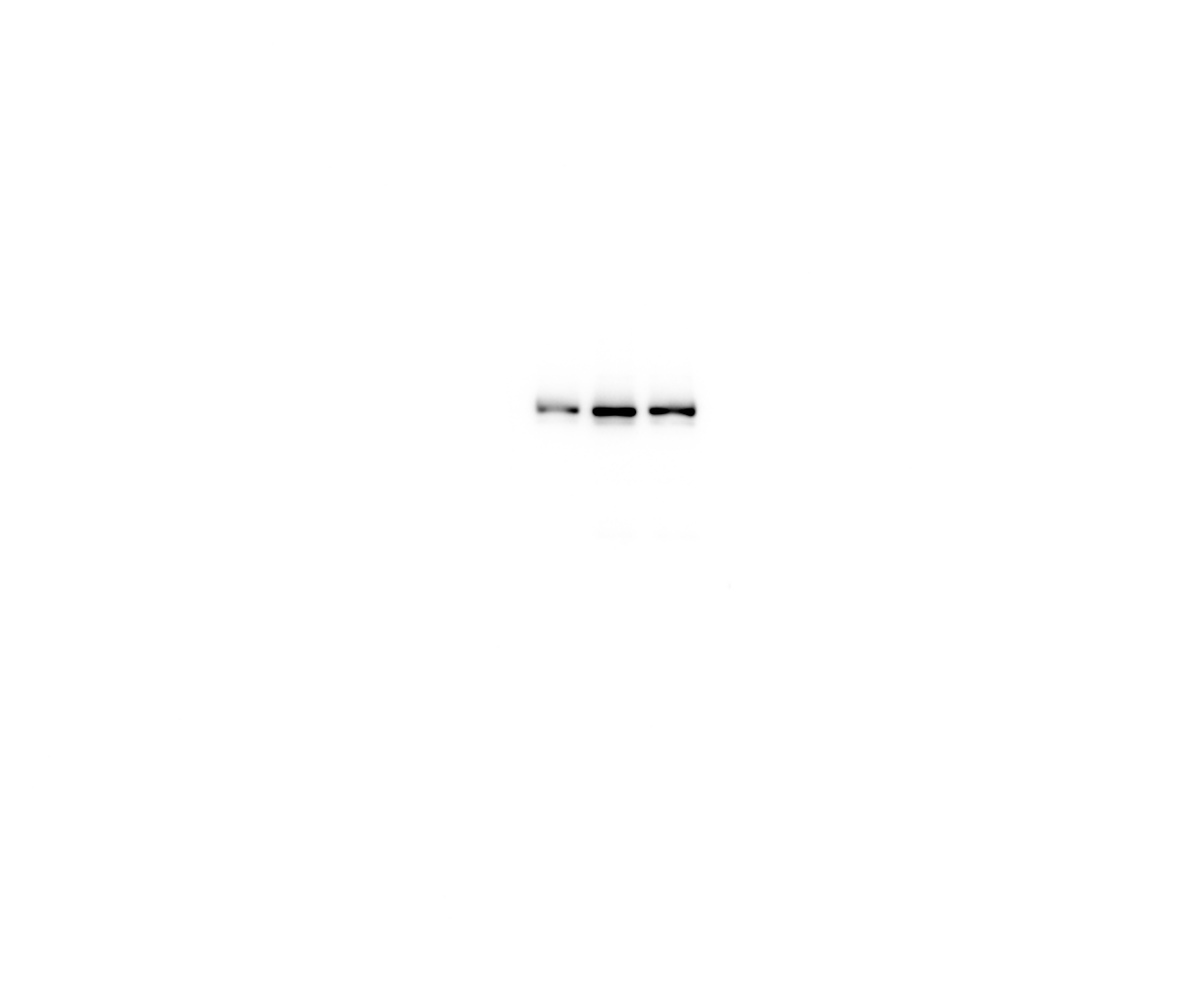

Supplement: S1 Raw data — (ZIP) [file pone.0317738.s002.zip › S1 Raw data/repeat 1/STAT3.Tif]

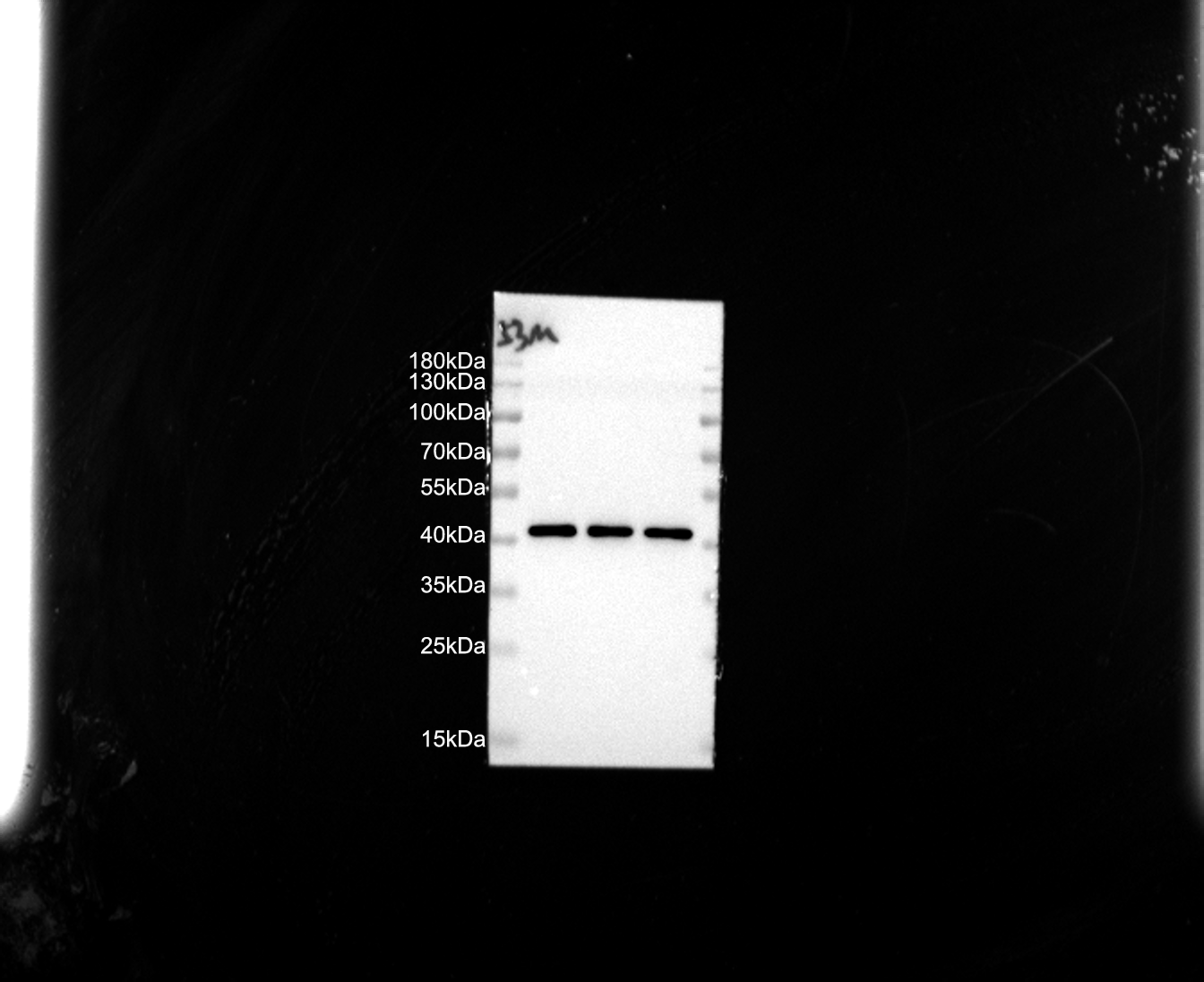

Supplement: S1 Raw data — (ZIP) [file pone.0317738.s002.zip › S1 Raw data/repeat 2/ACTIN..Tif]

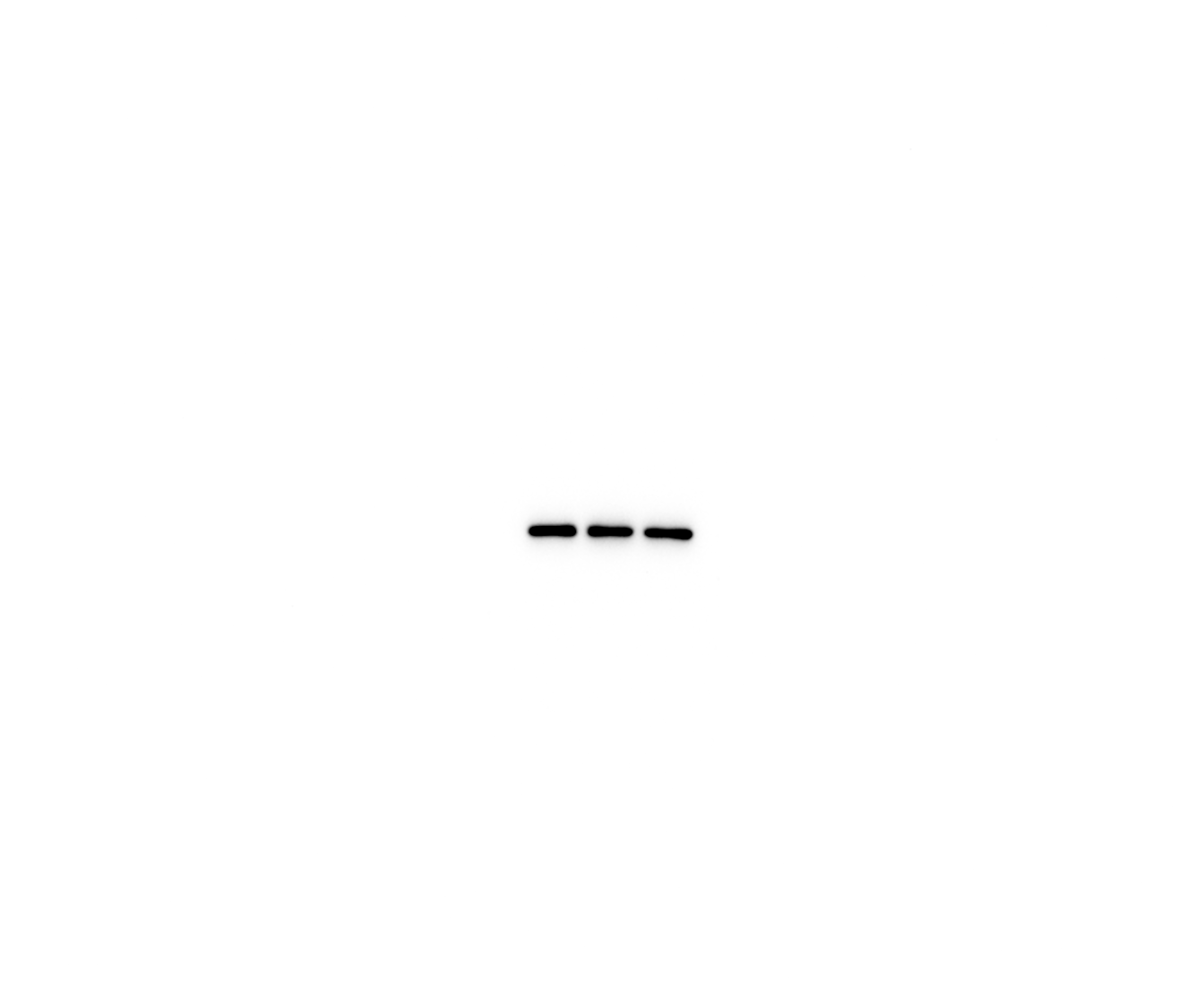

Supplement: S1 Raw data — (ZIP) [file pone.0317738.s002.zip › S1 Raw data/repeat 2/ACTIN.Tif]

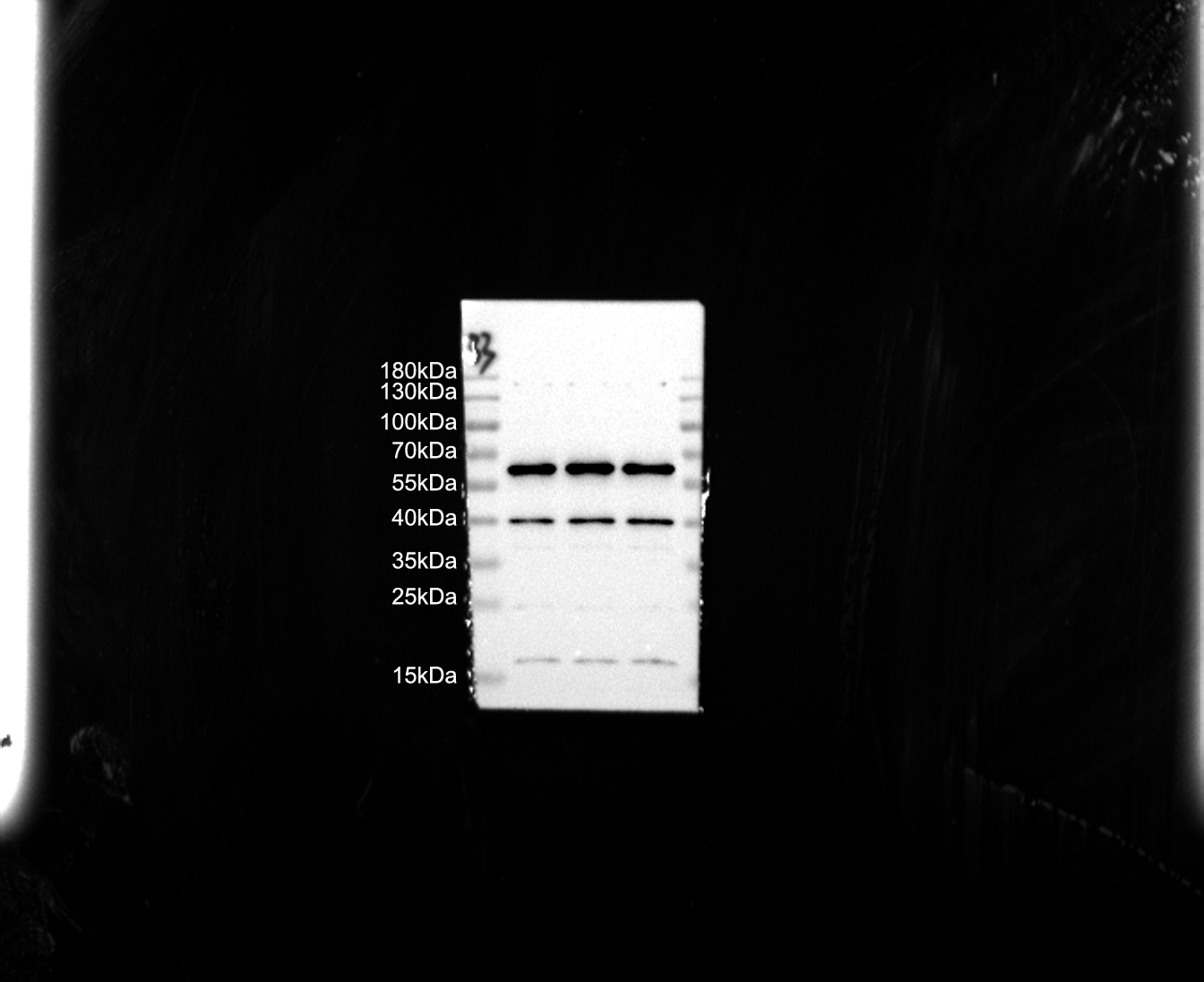

Supplement: S1 Raw data — (ZIP) [file pone.0317738.s002.zip › S1 Raw data/repeat 2/AKT..Tif]

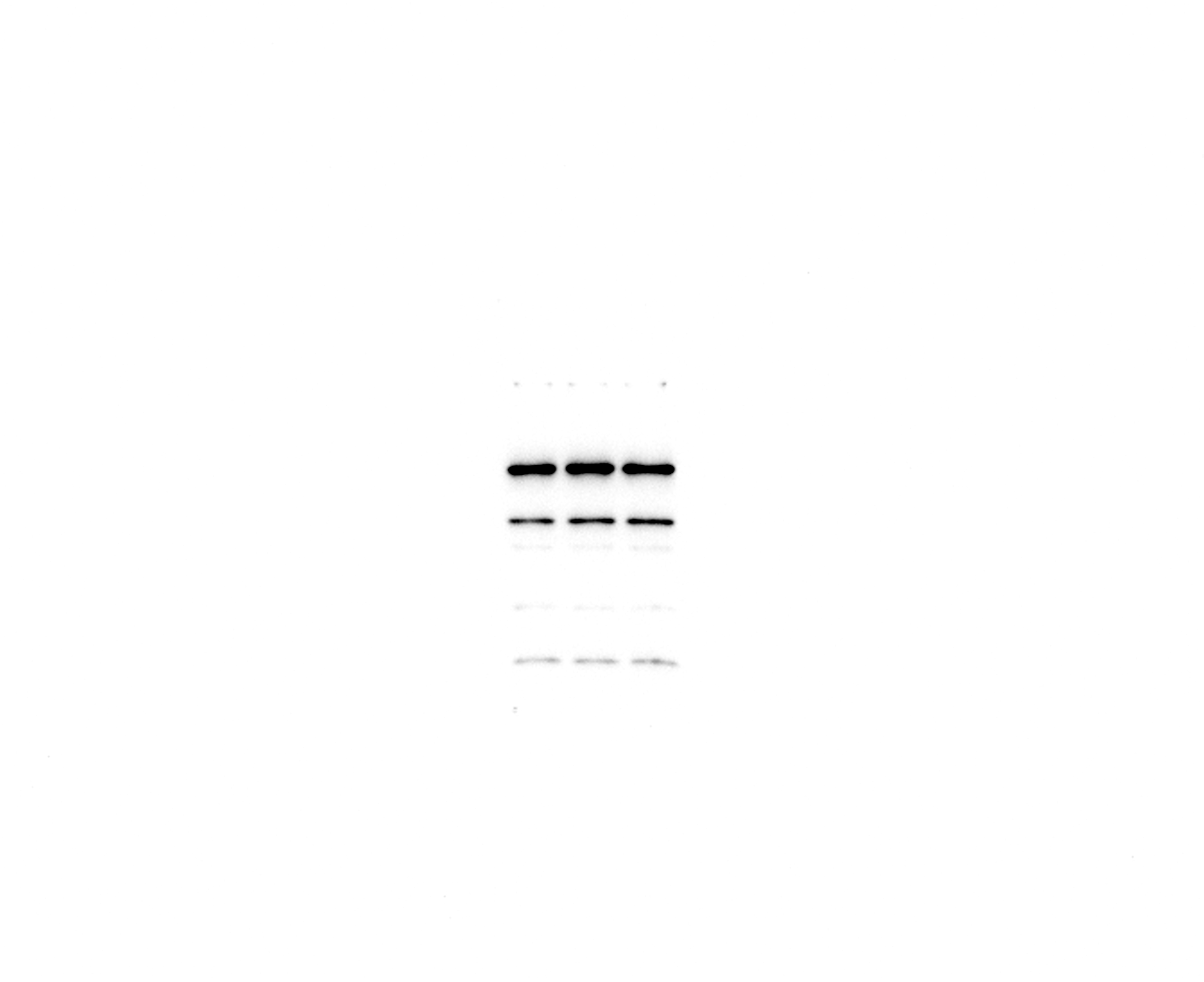

Supplement: S1 Raw data — (ZIP) [file pone.0317738.s002.zip › S1 Raw data/repeat 2/AKT.Tif]

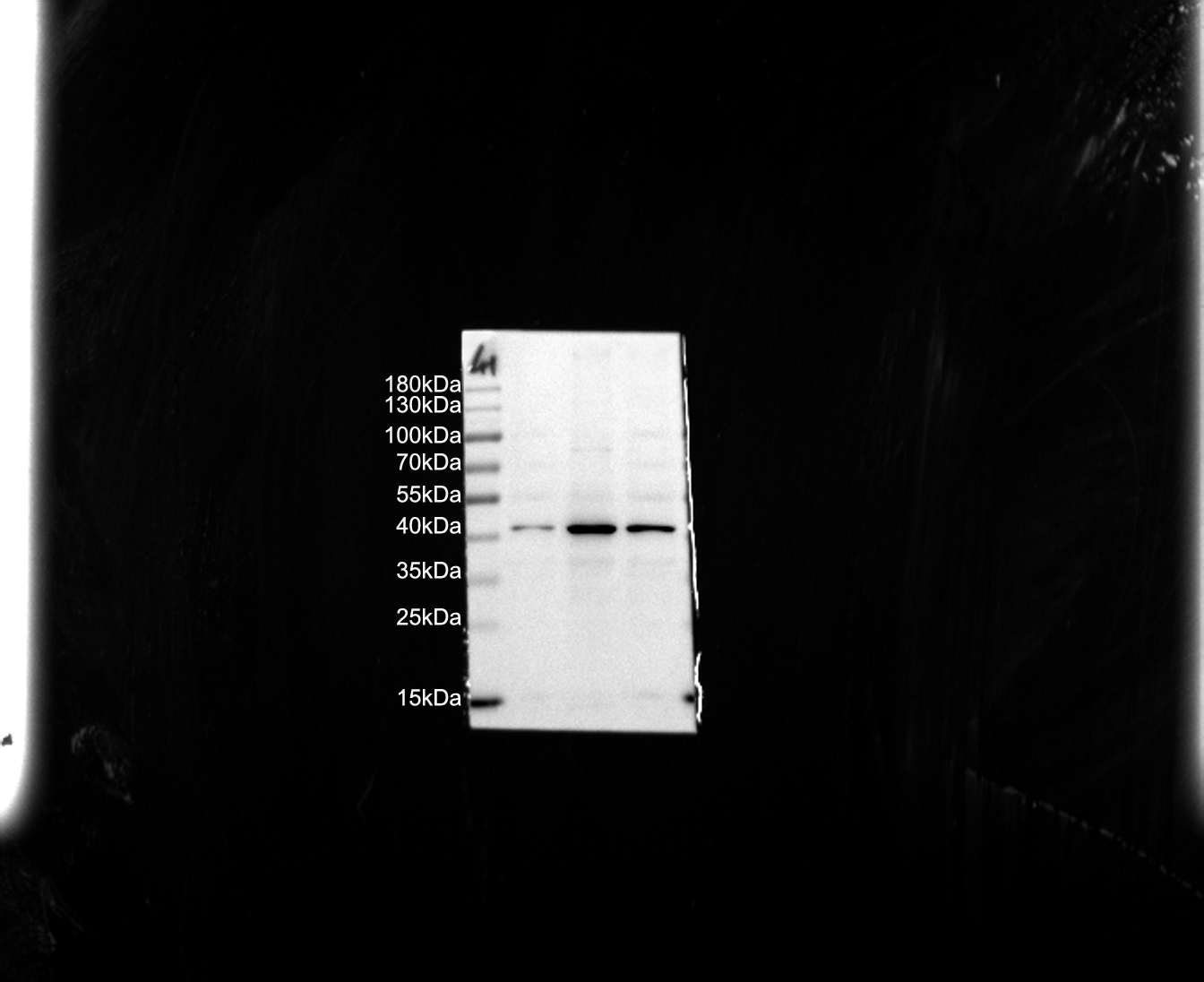

Supplement: S1 Raw data — (ZIP) [file pone.0317738.s002.zip › S1 Raw data/repeat 2/CAS9..Tif]

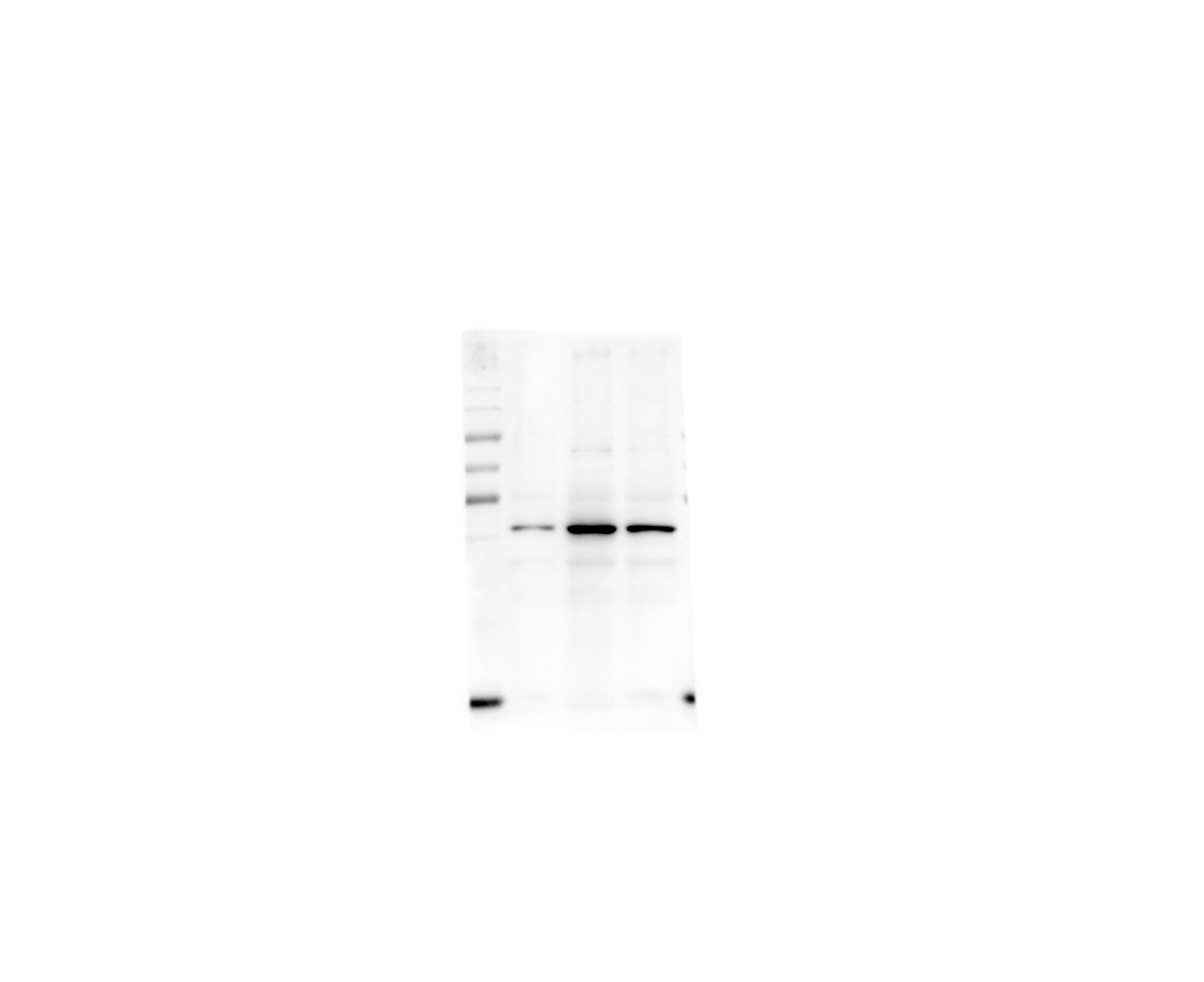

Supplement: S1 Raw data — (ZIP) [file pone.0317738.s002.zip › S1 Raw data/repeat 2/CAS9.Tif]

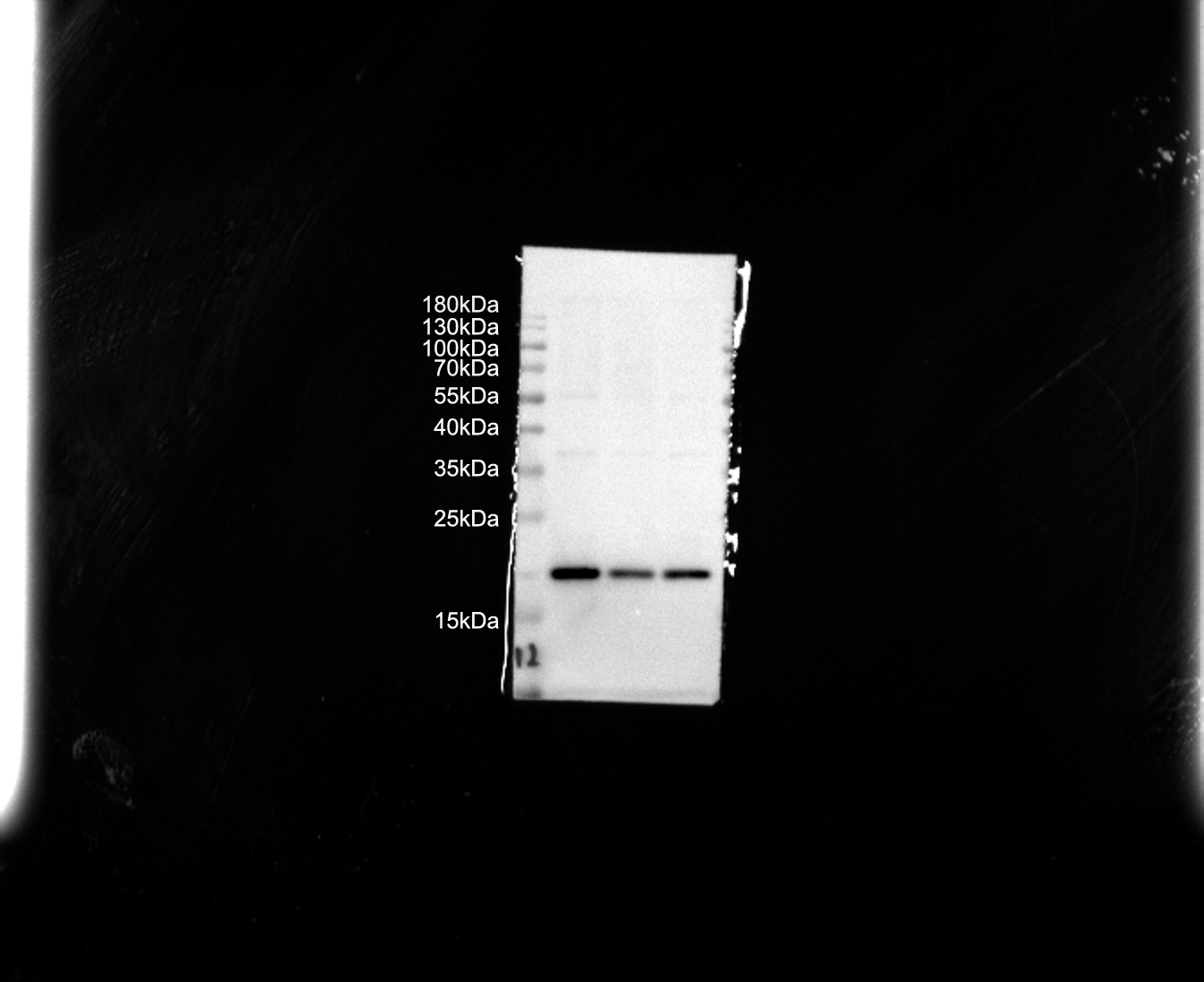

Supplement: S1 Raw data — (ZIP) [file pone.0317738.s002.zip › S1 Raw data/repeat 2/FGF1..Tif]

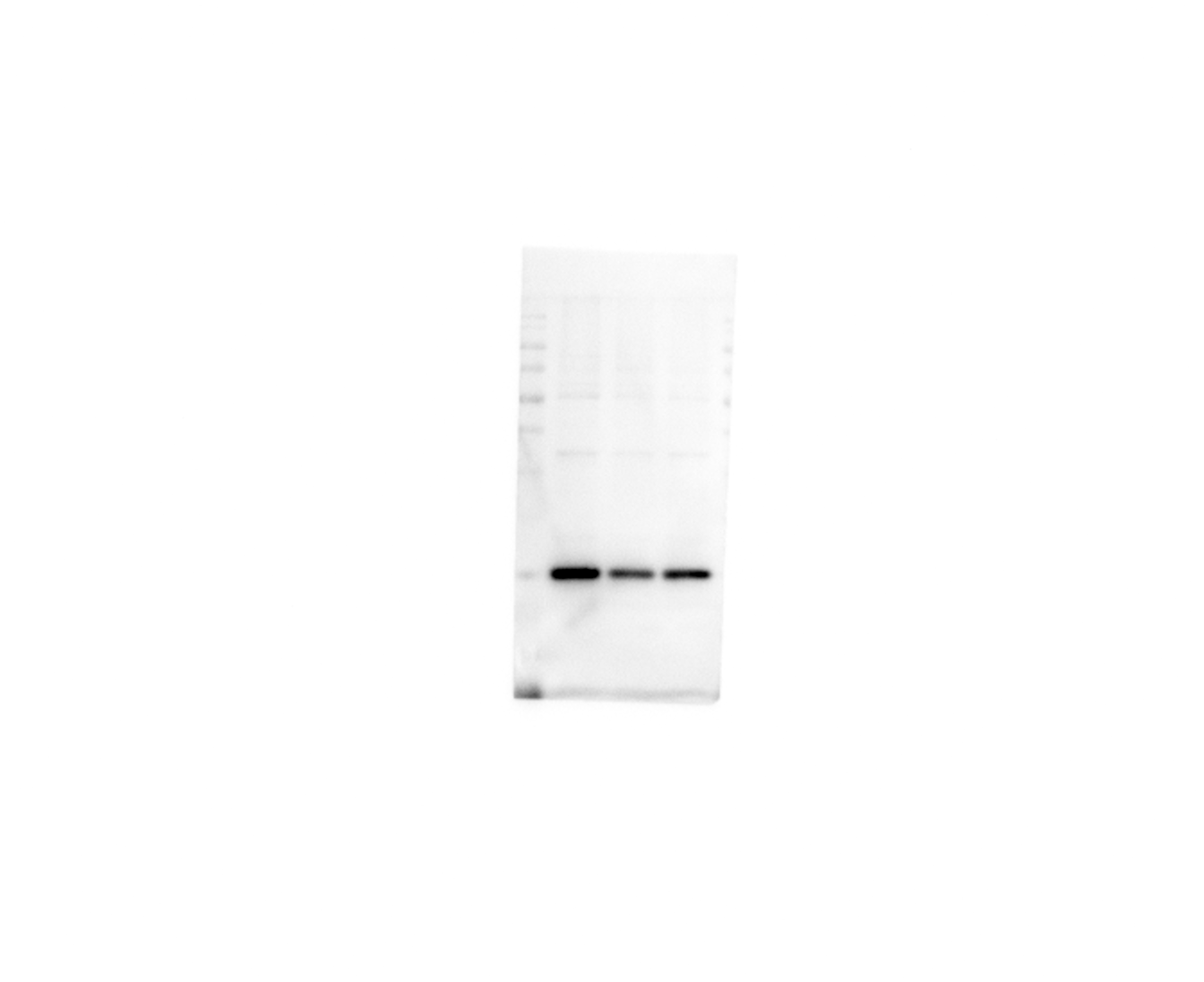

Supplement: S1 Raw data — (ZIP) [file pone.0317738.s002.zip › S1 Raw data/repeat 2/FGF1.Tif]

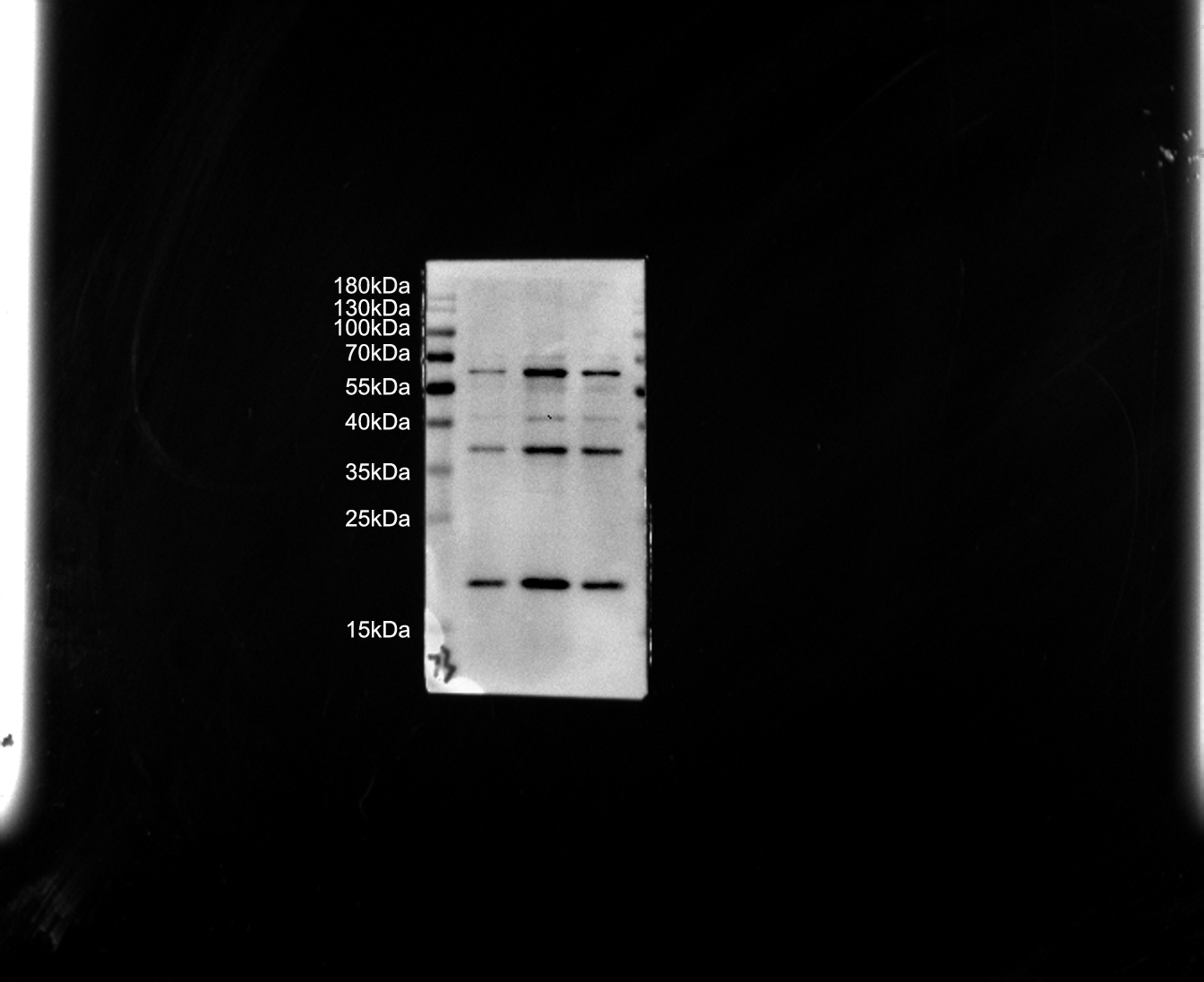

Supplement: S1 Raw data — (ZIP) [file pone.0317738.s002.zip › S1 Raw data/repeat 2/P-AKT..Tif]

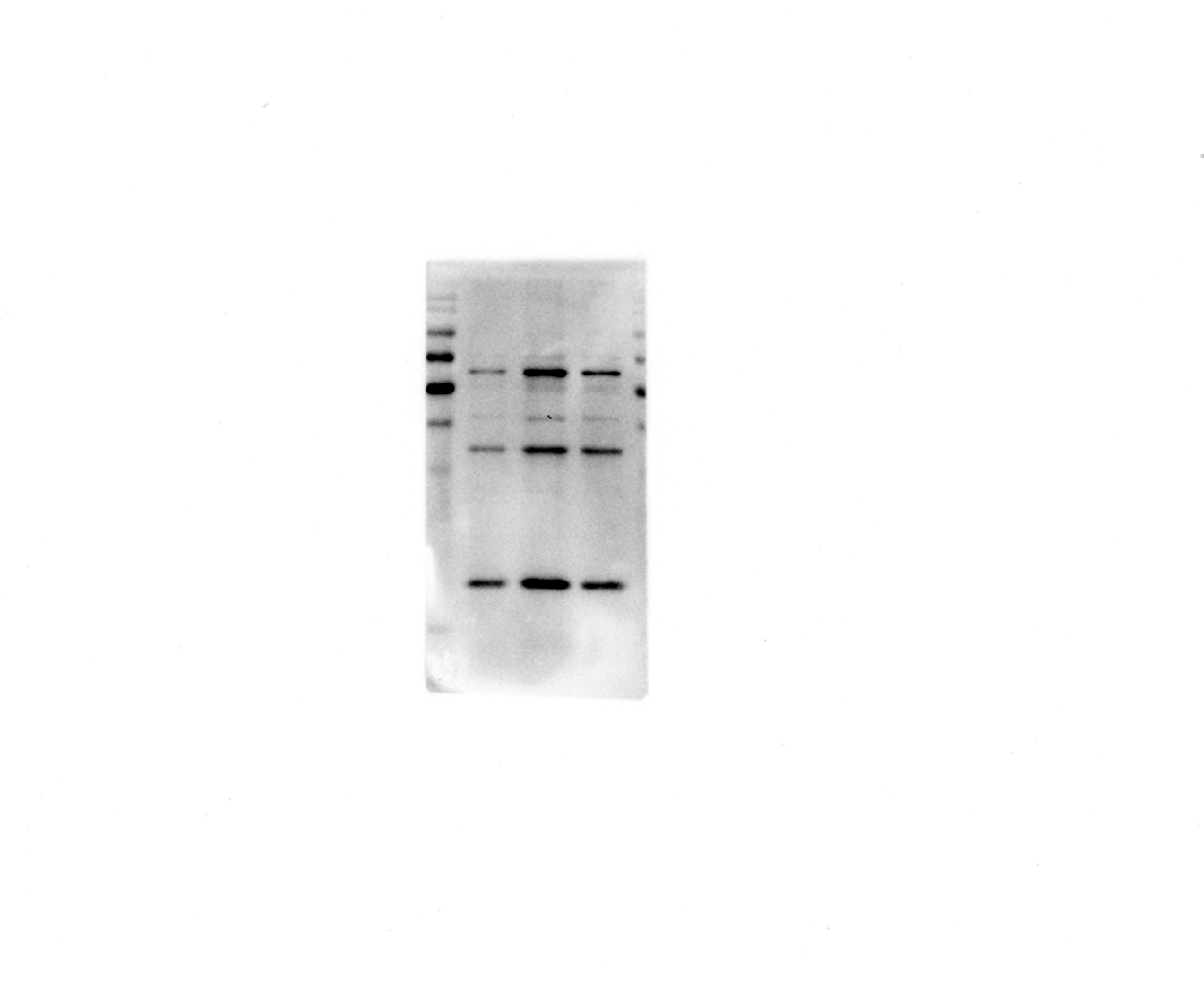

Supplement: S1 Raw data — (ZIP) [file pone.0317738.s002.zip › S1 Raw data/repeat 2/P-AKT.Tif]

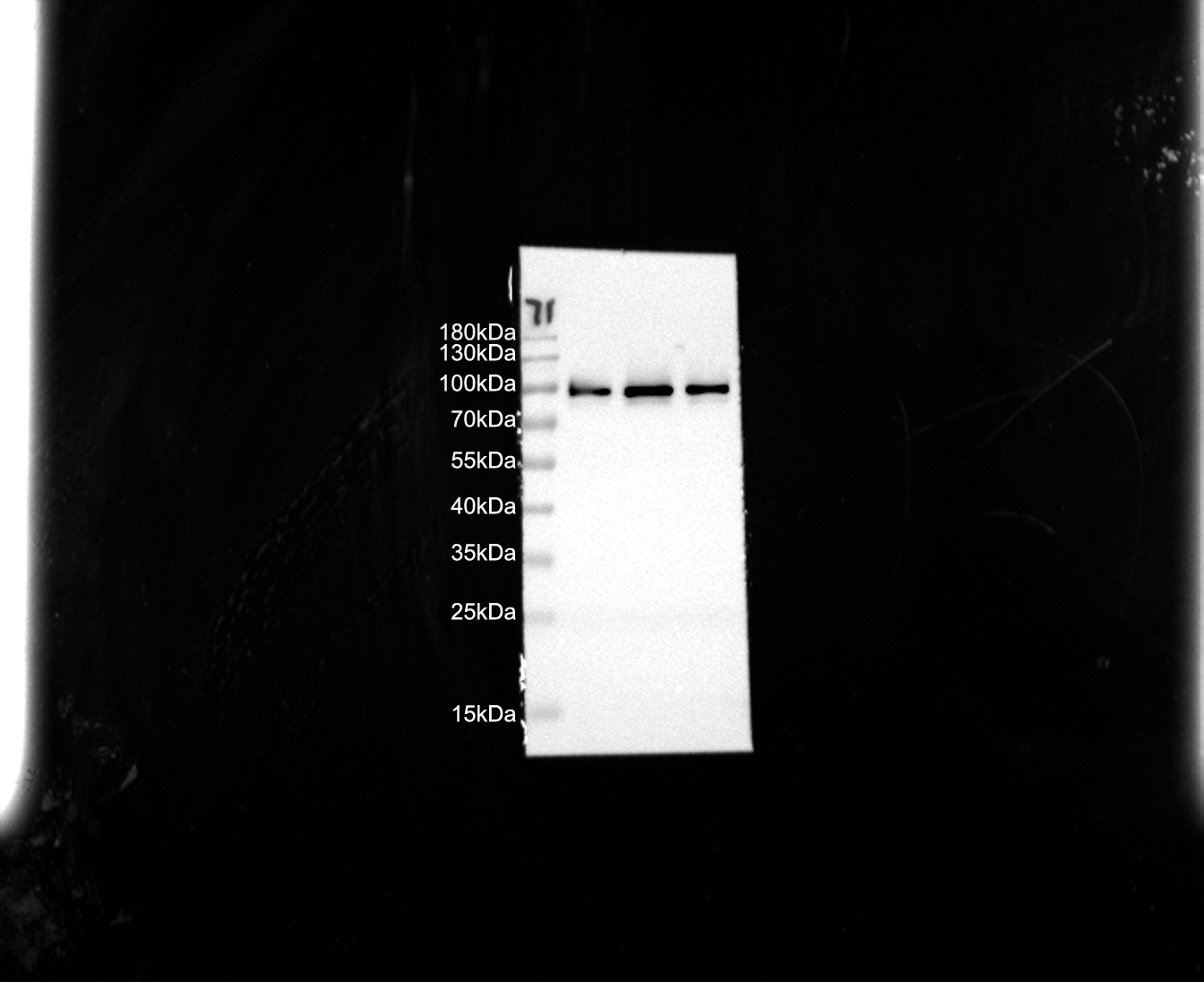

Supplement: S1 Raw data — (ZIP) [file pone.0317738.s002.zip › S1 Raw data/repeat 2/STAT3..Tif]

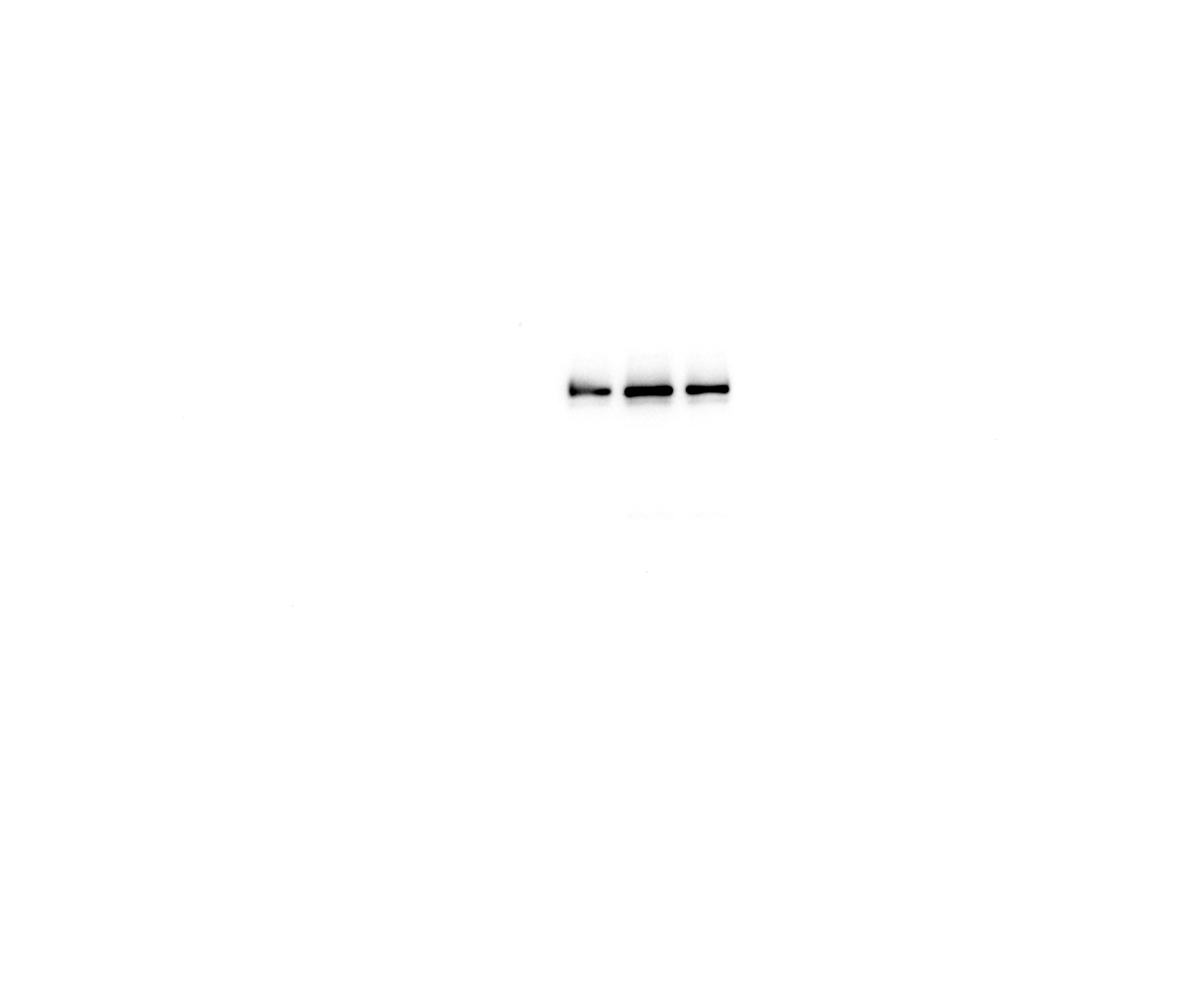

Supplement: S1 Raw data — (ZIP) [file pone.0317738.s002.zip › S1 Raw data/repeat 2/STAT3.Tif]

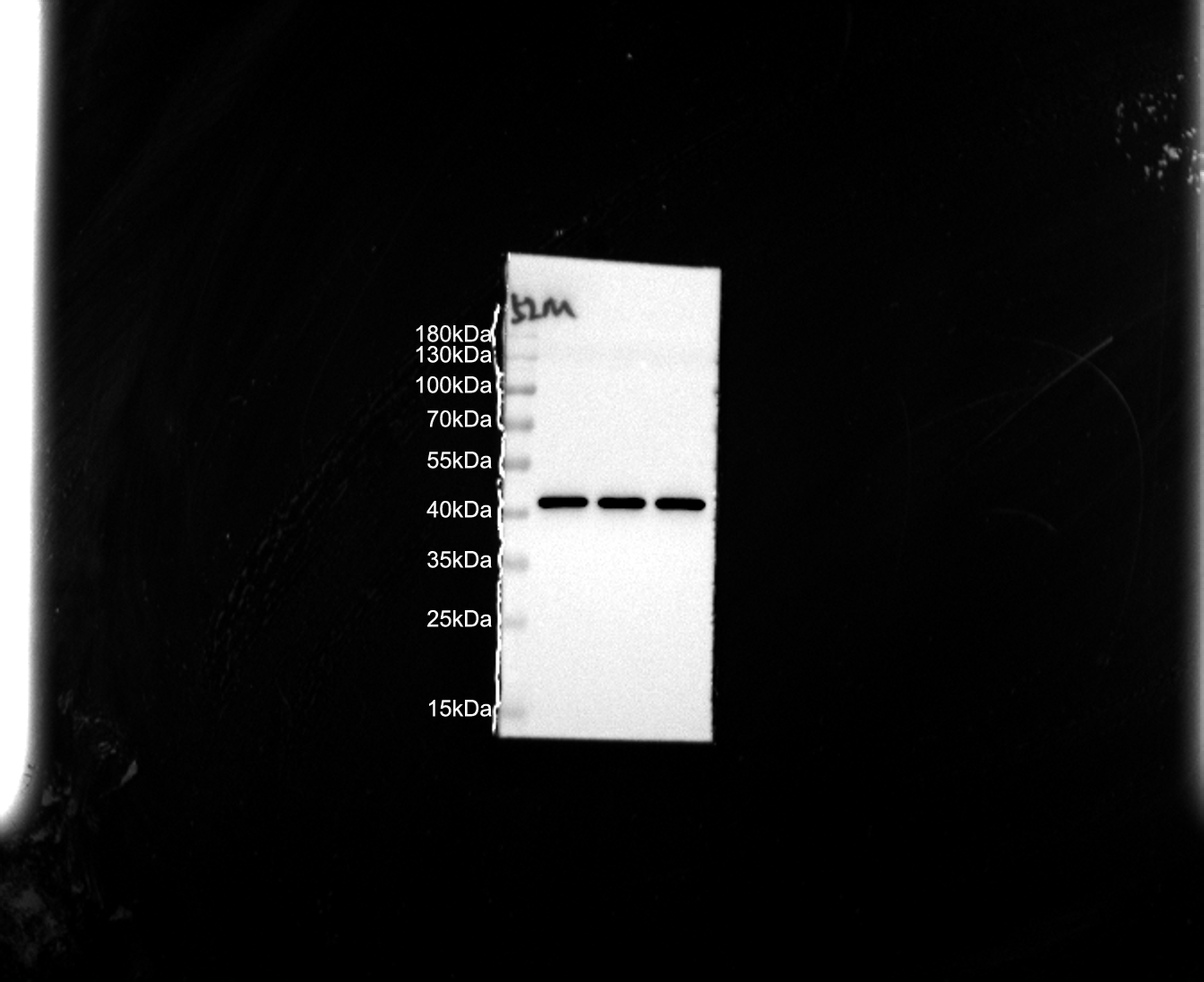

Supplement: S1 Raw data — (ZIP) [file pone.0317738.s002.zip › S1 Raw data/repeat 3/ACTIN..Tif]

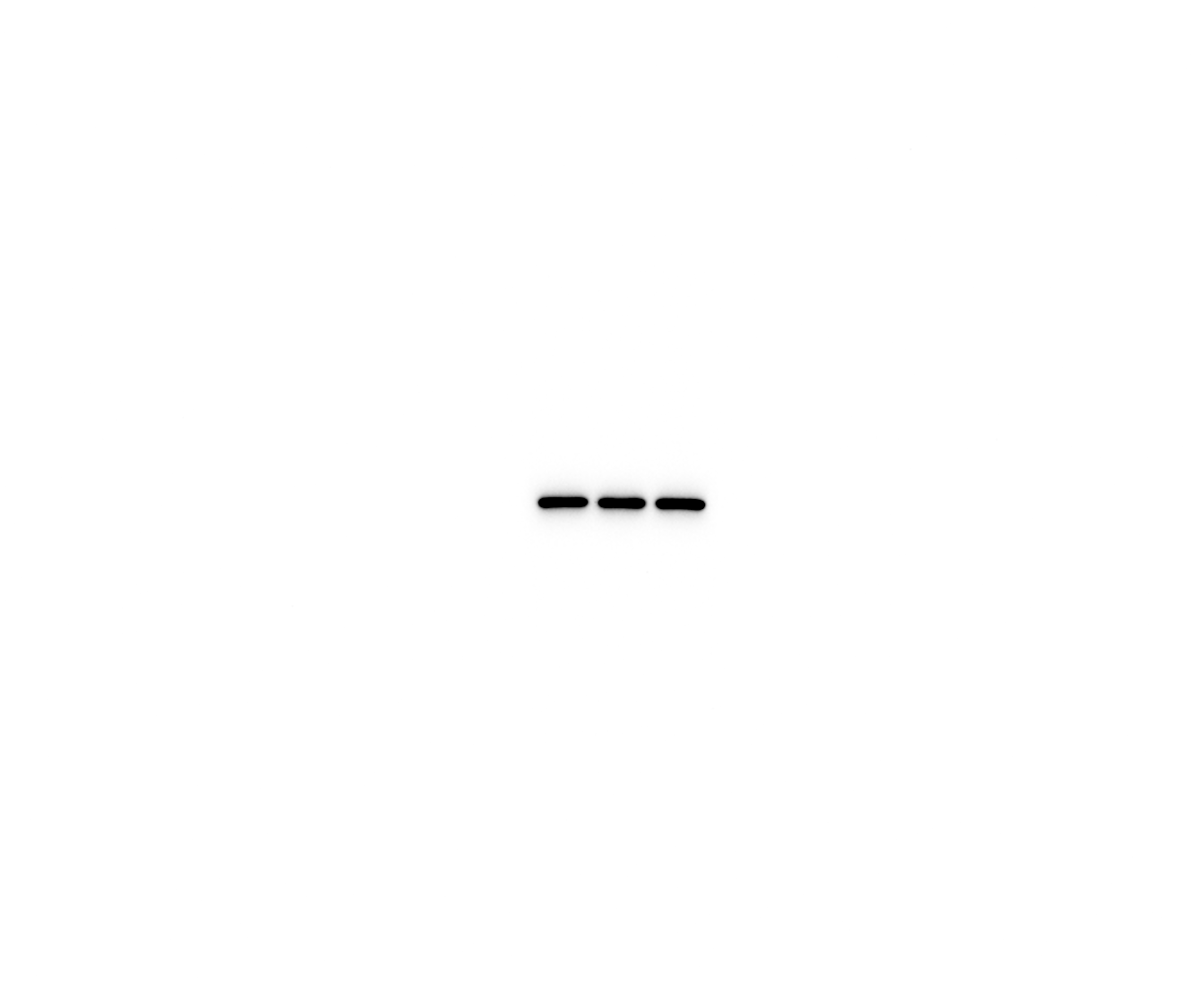

Supplement: S1 Raw data — (ZIP) [file pone.0317738.s002.zip › S1 Raw data/repeat 3/ACTIN.Tif]

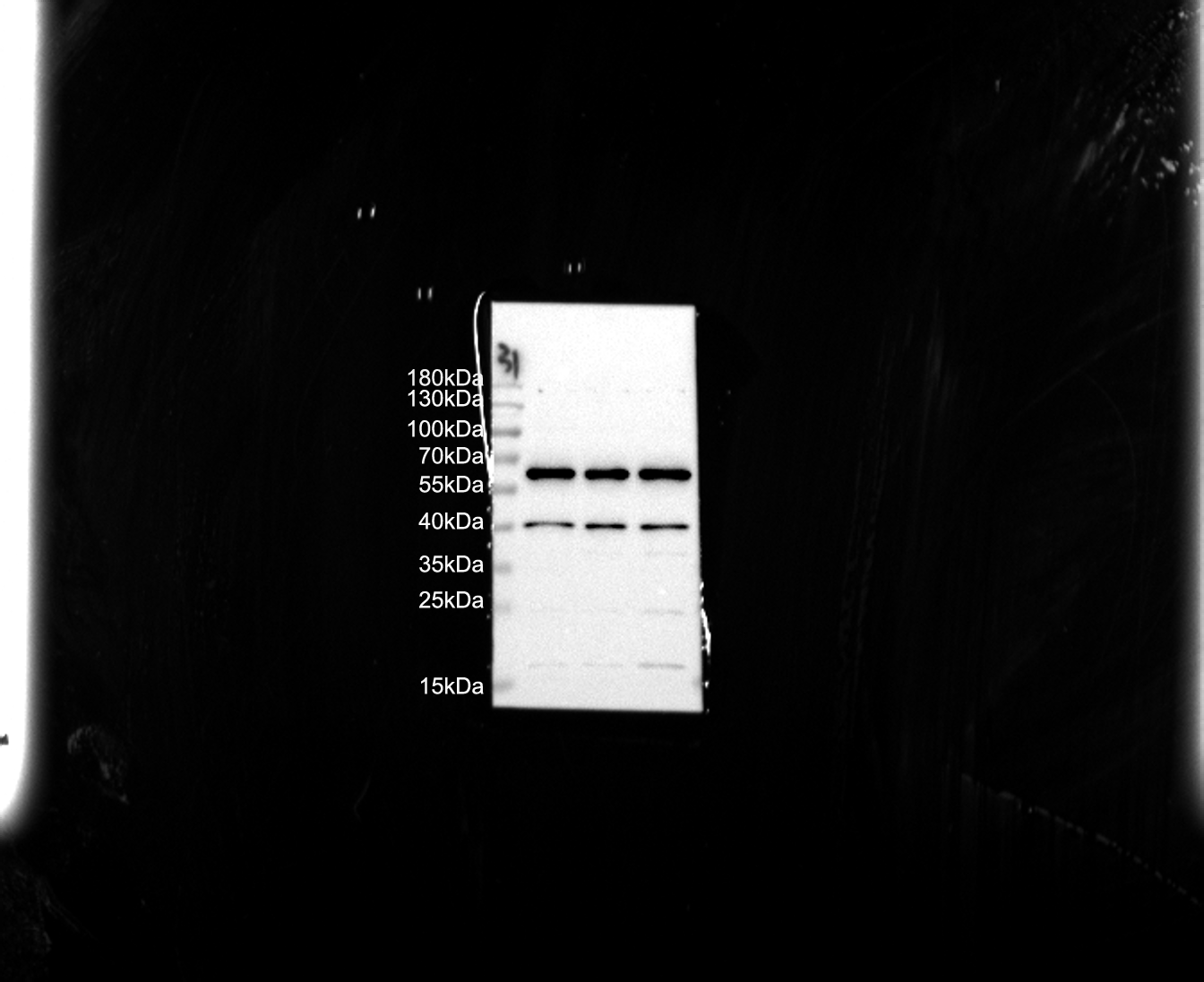

Supplement: S1 Raw data — (ZIP) [file pone.0317738.s002.zip › S1 Raw data/repeat 3/AKT..Tif]

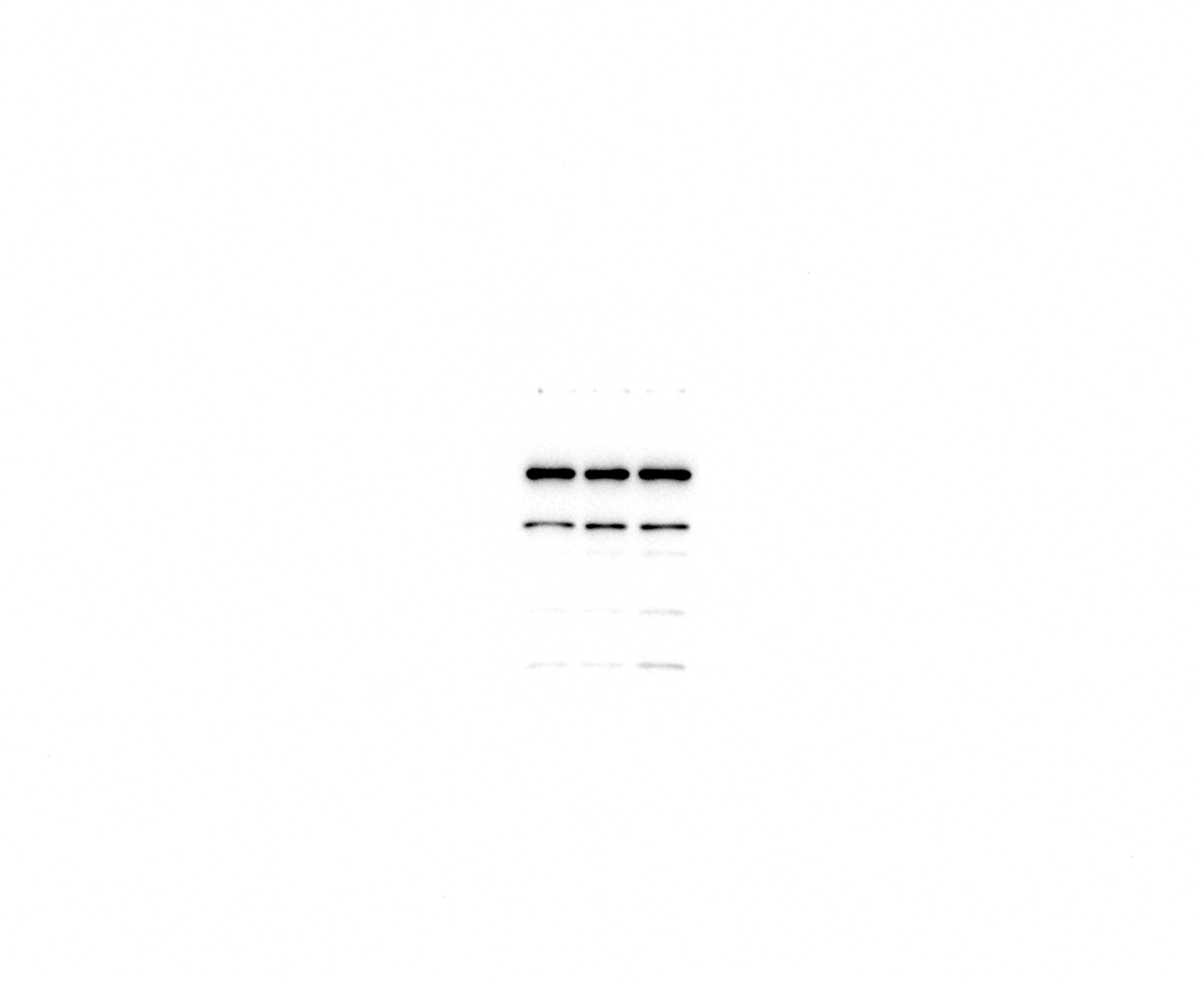

Supplement: S1 Raw data — (ZIP) [file pone.0317738.s002.zip › S1 Raw data/repeat 3/AKT.Tif]

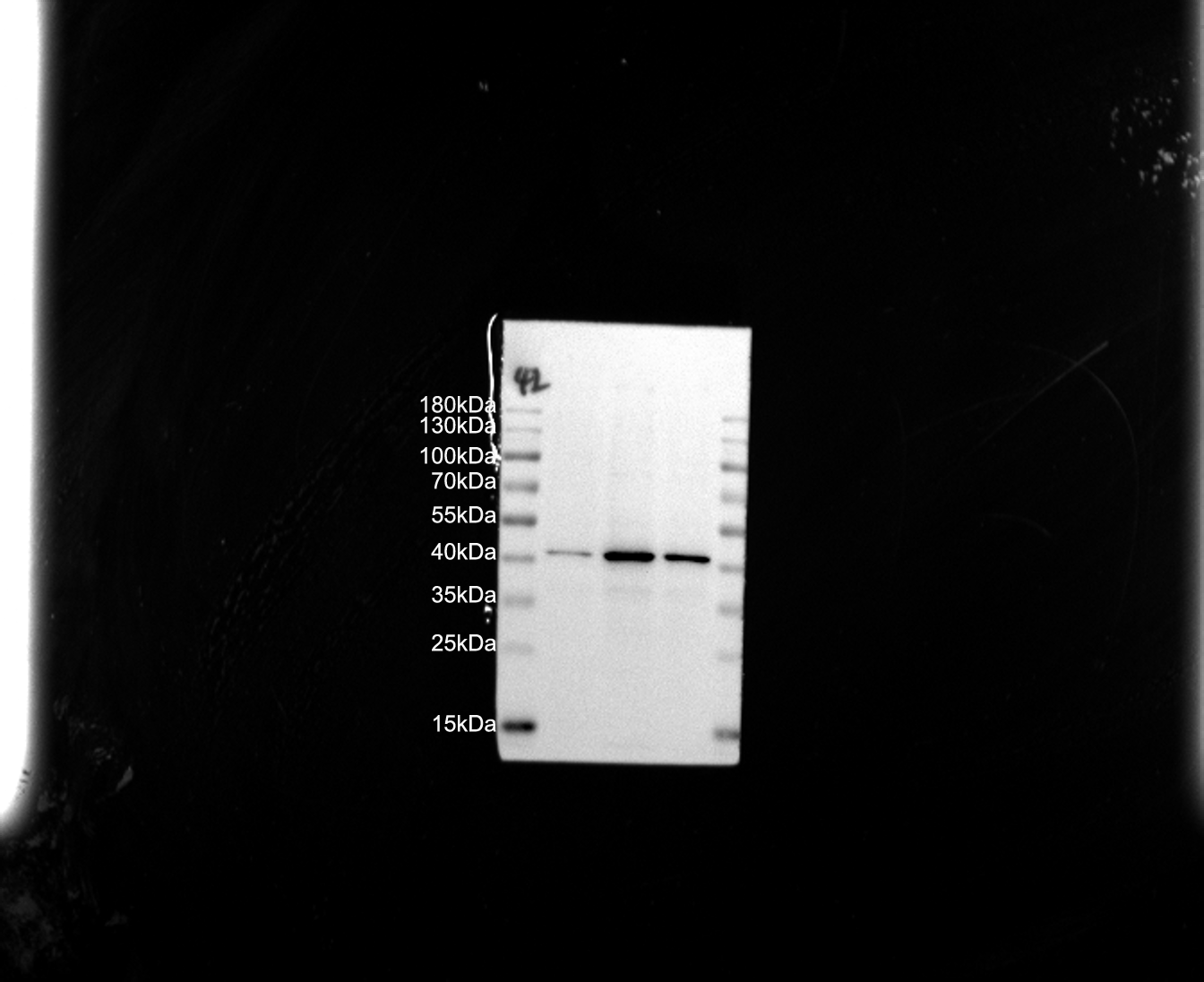

Supplement: S1 Raw data — (ZIP) [file pone.0317738.s002.zip › S1 Raw data/repeat 3/CAS9..Tif]

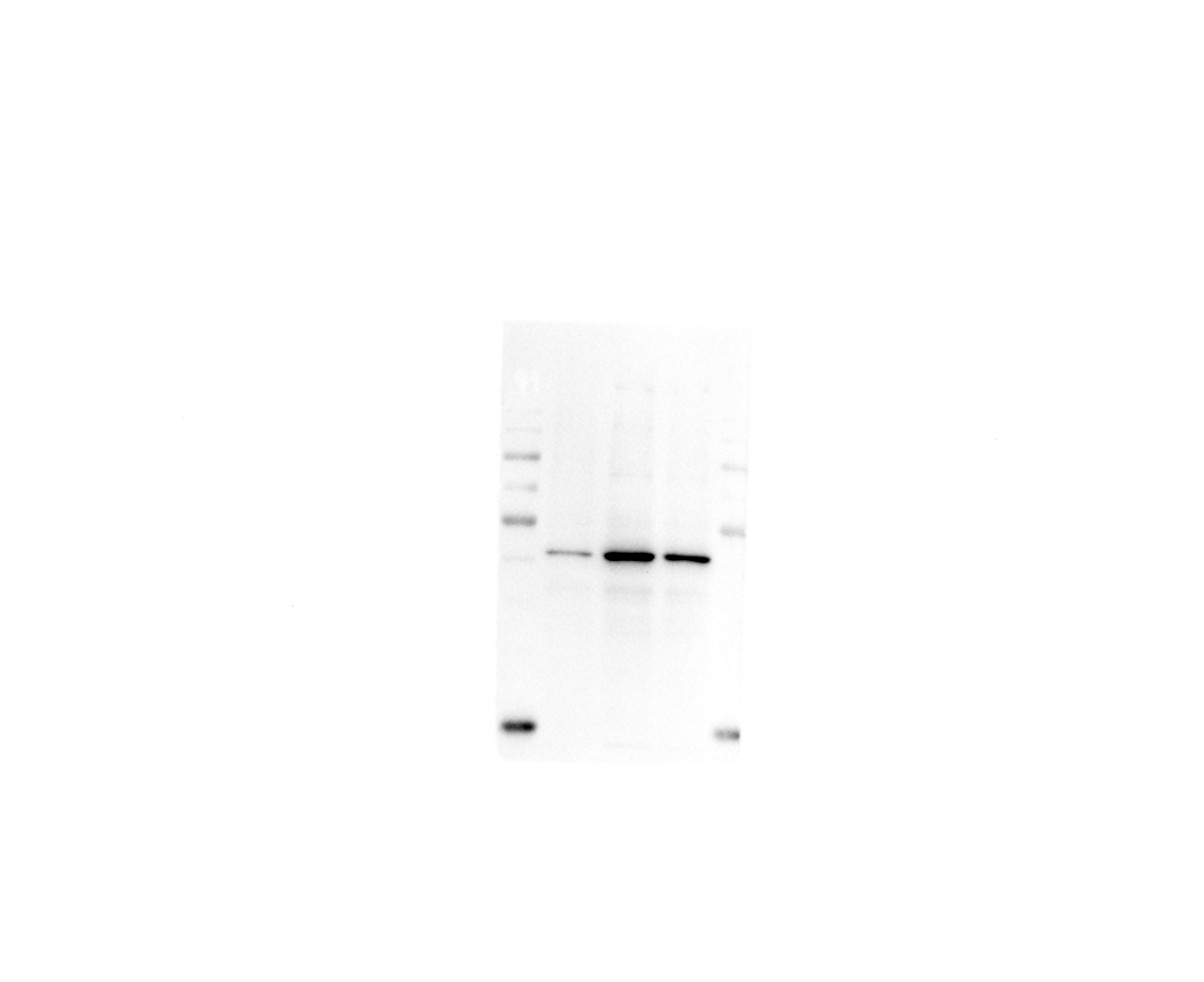

Supplement: S1 Raw data — (ZIP) [file pone.0317738.s002.zip › S1 Raw data/repeat 3/CAS9.Tif]

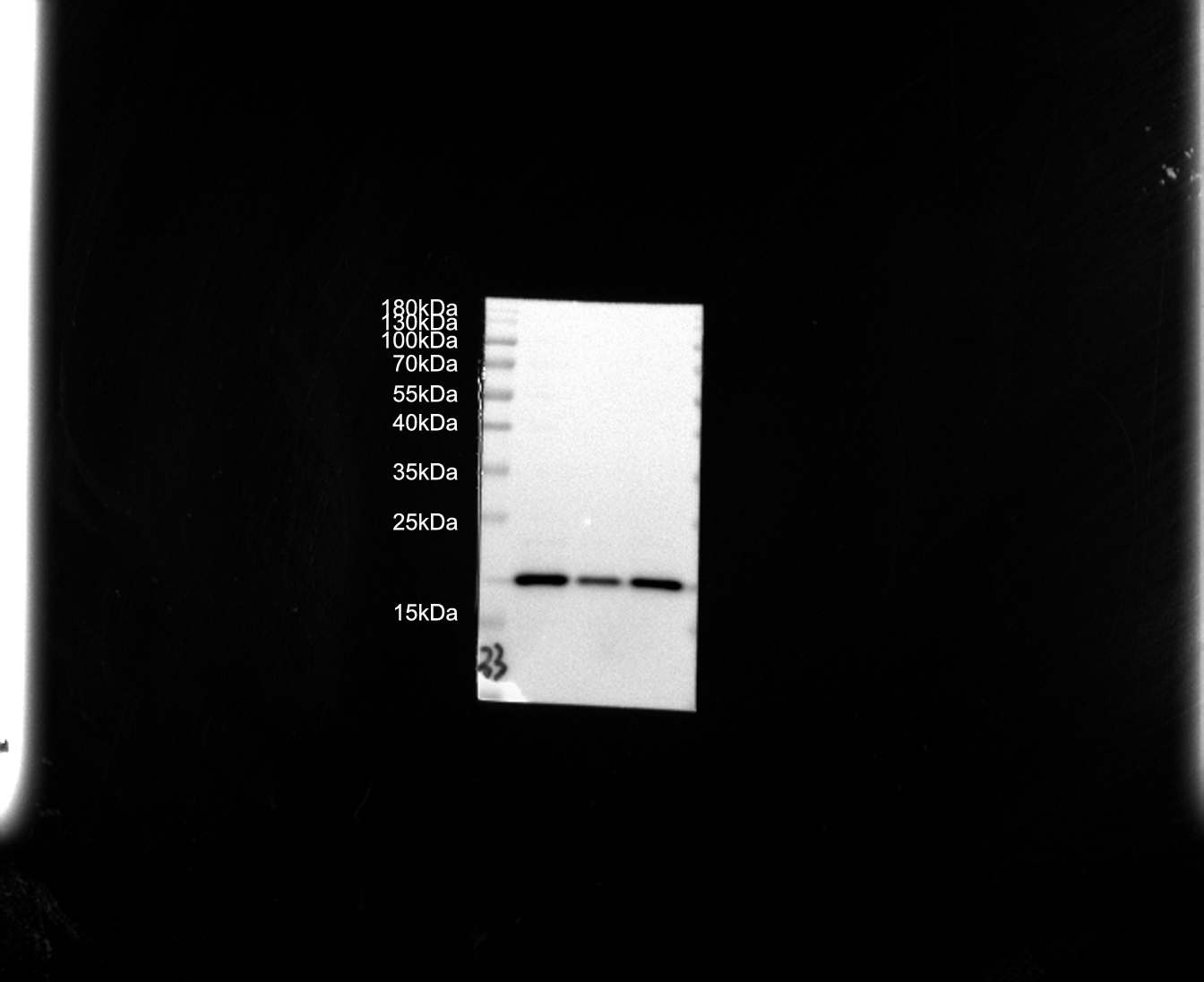

Supplement: S1 Raw data — (ZIP) [file pone.0317738.s002.zip › S1 Raw data/repeat 3/FGF1..Tif]

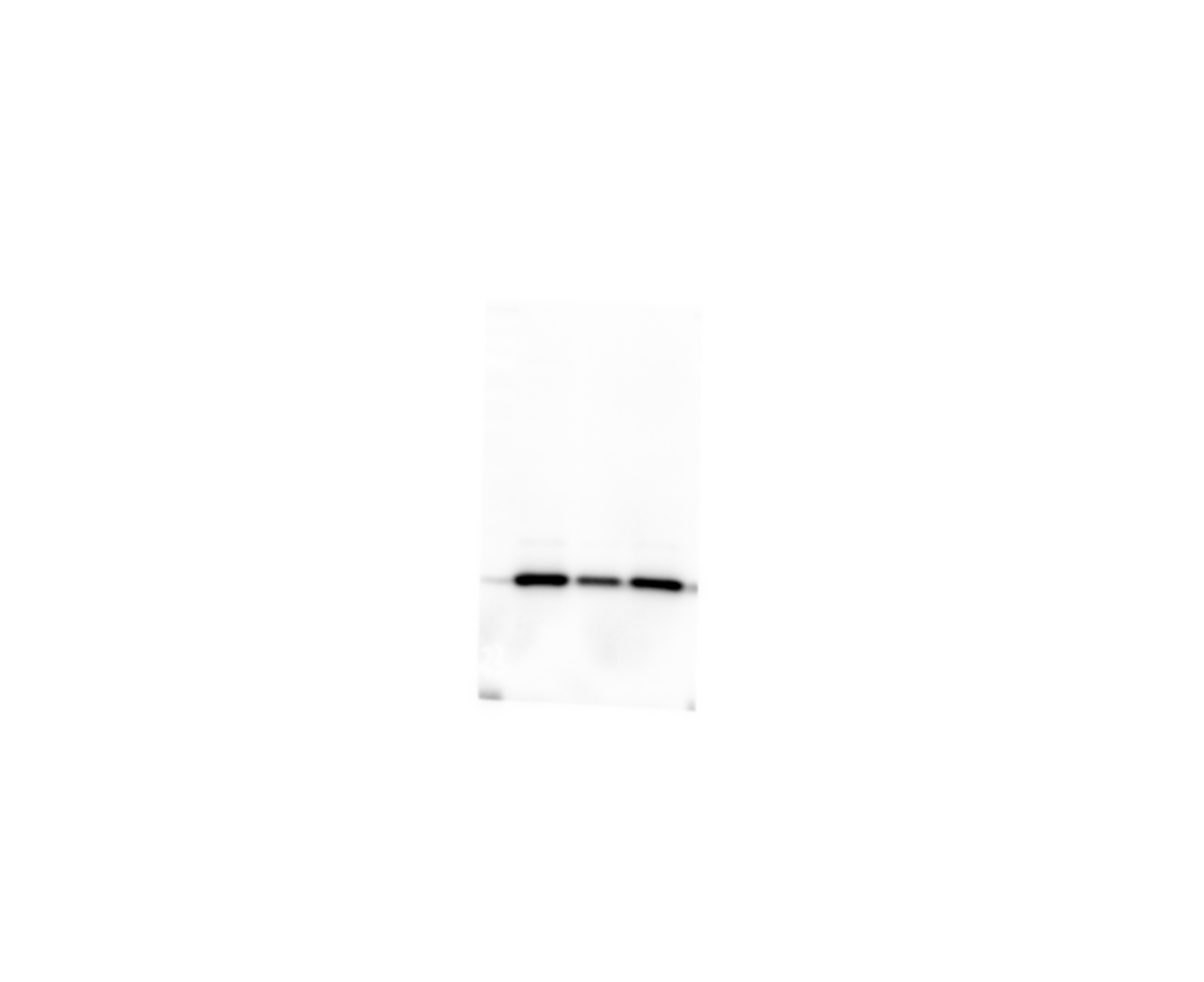

Supplement: S1 Raw data — (ZIP) [file pone.0317738.s002.zip › S1 Raw data/repeat 3/FGF1.Tif]

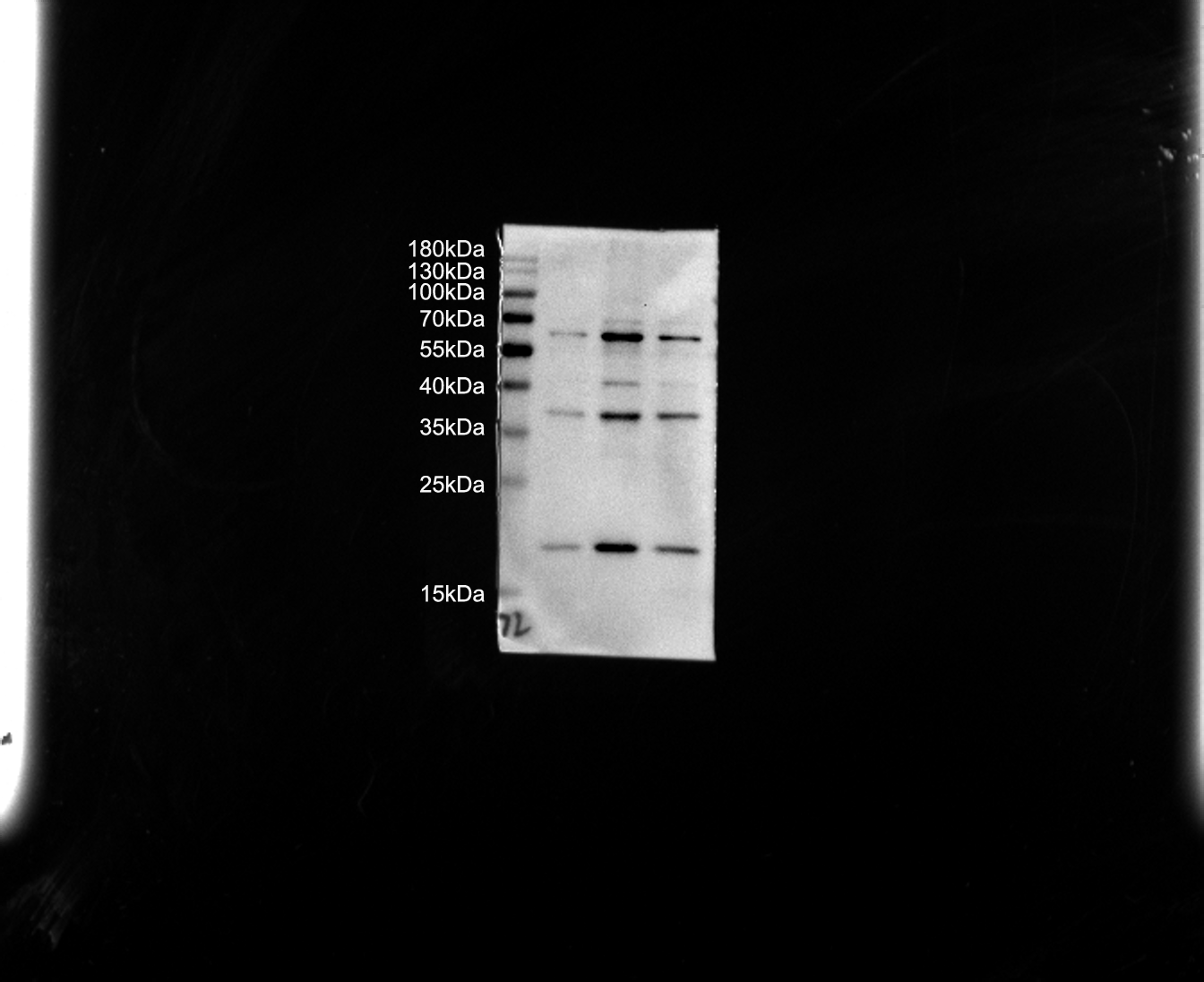

Supplement: S1 Raw data — (ZIP) [file pone.0317738.s002.zip › S1 Raw data/repeat 3/P-AKT..Tif]

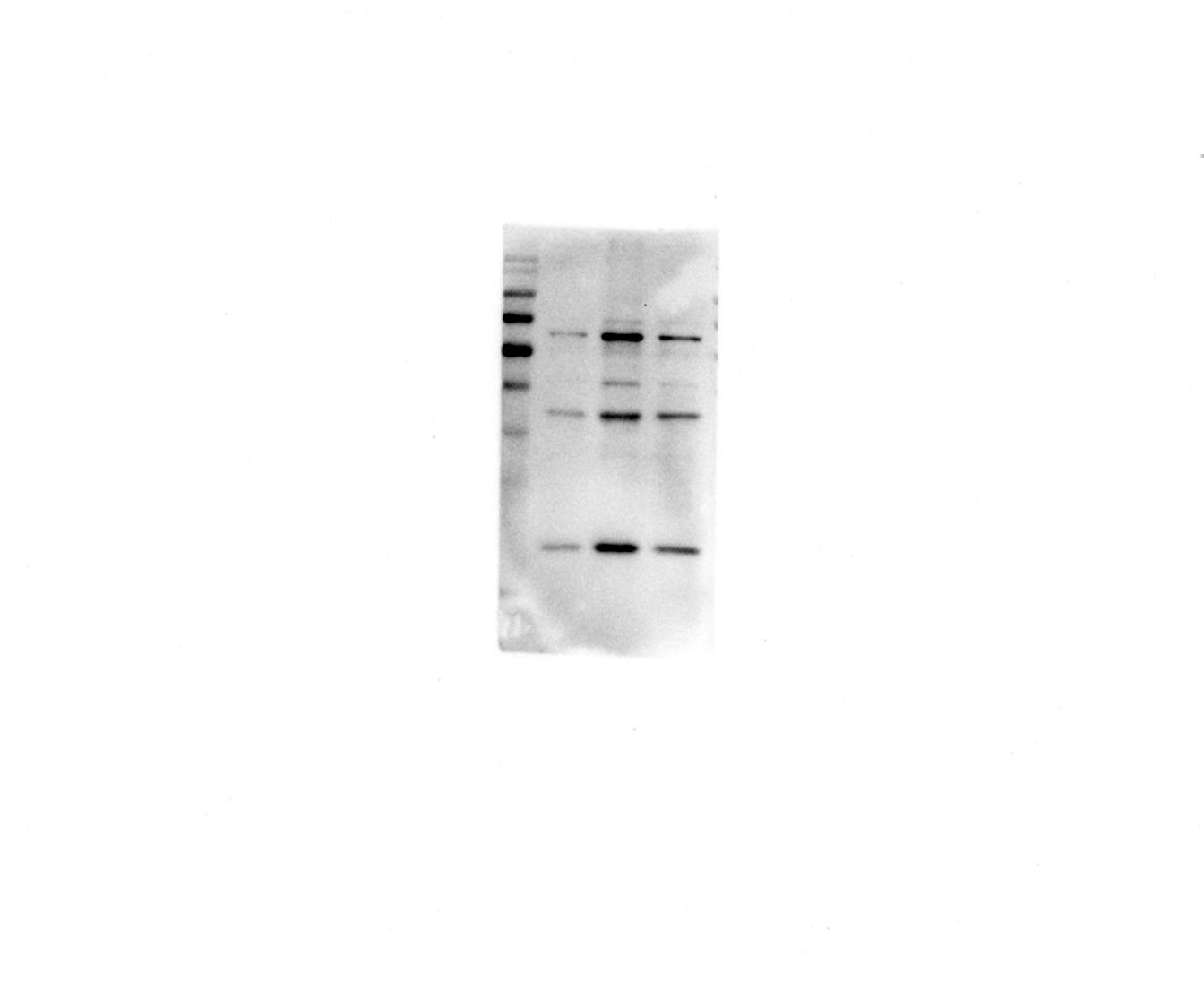

Supplement: S1 Raw data — (ZIP) [file pone.0317738.s002.zip › S1 Raw data/repeat 3/P-AKT.Tif]

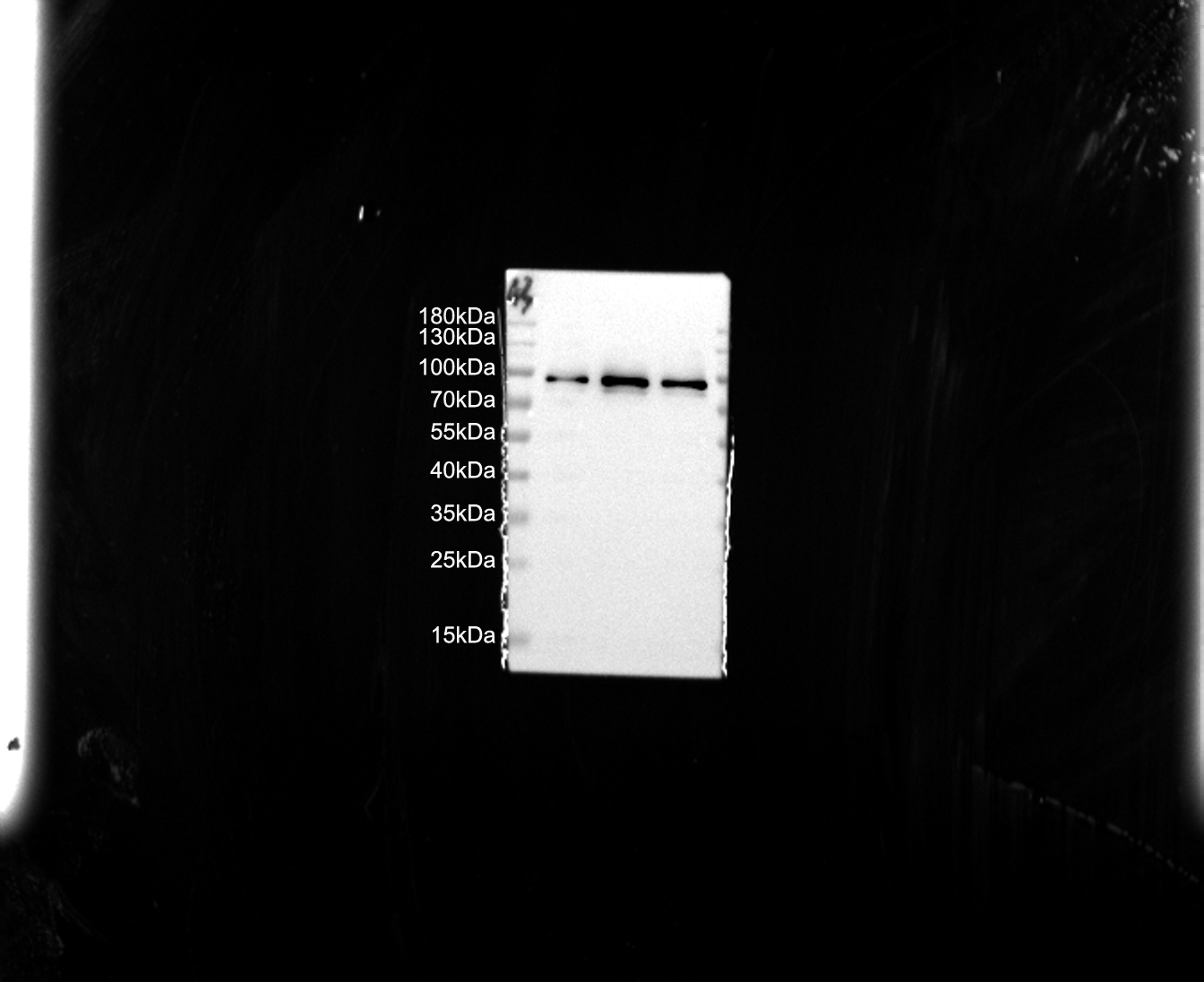

Supplement: S1 Raw data — (ZIP) [file pone.0317738.s002.zip › S1 Raw data/repeat 3/STAT3..Tif]

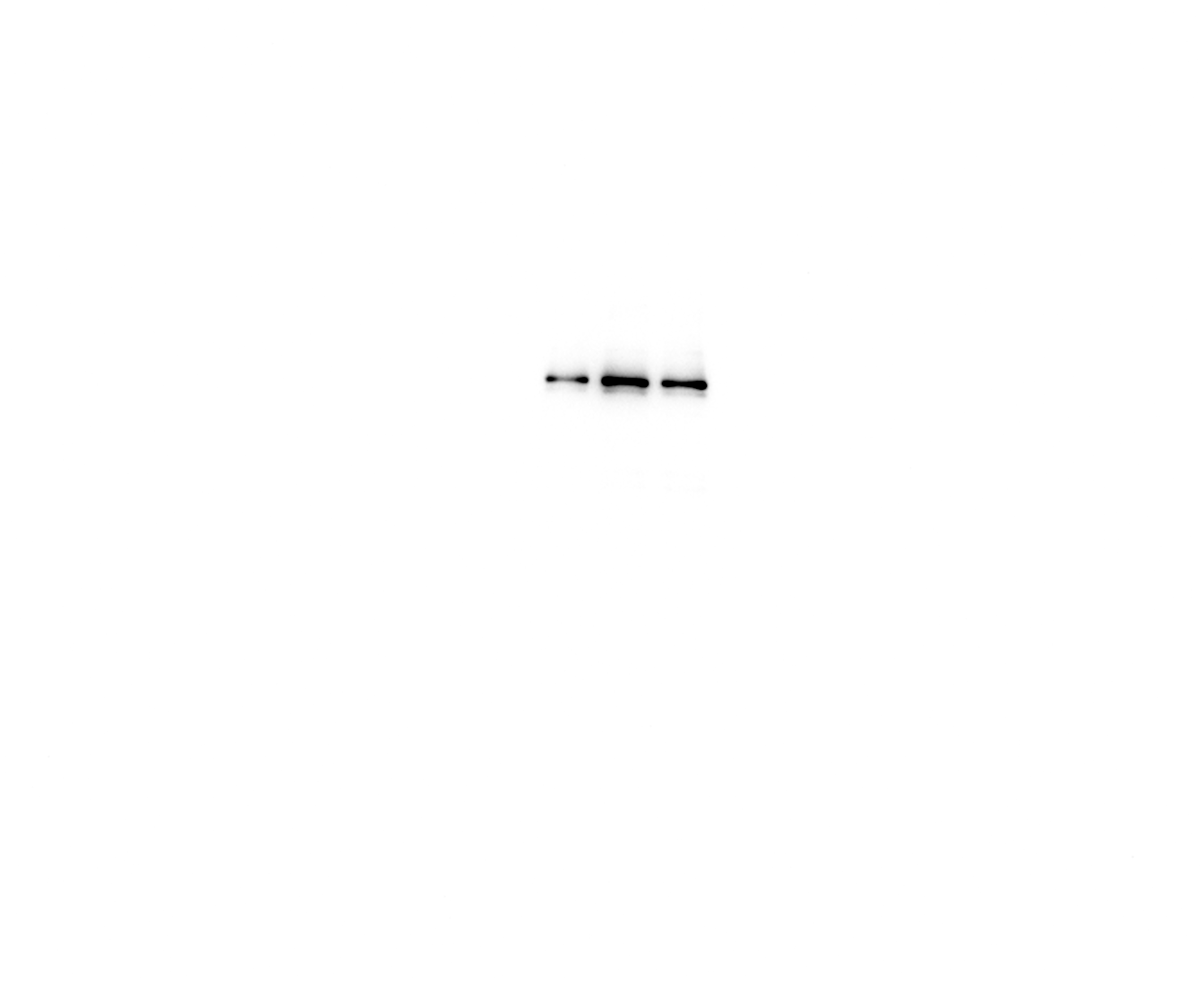

Supplement: S1 Raw data — (ZIP) [file pone.0317738.s002.zip › S1 Raw data/repeat 3/STAT3.Tif]

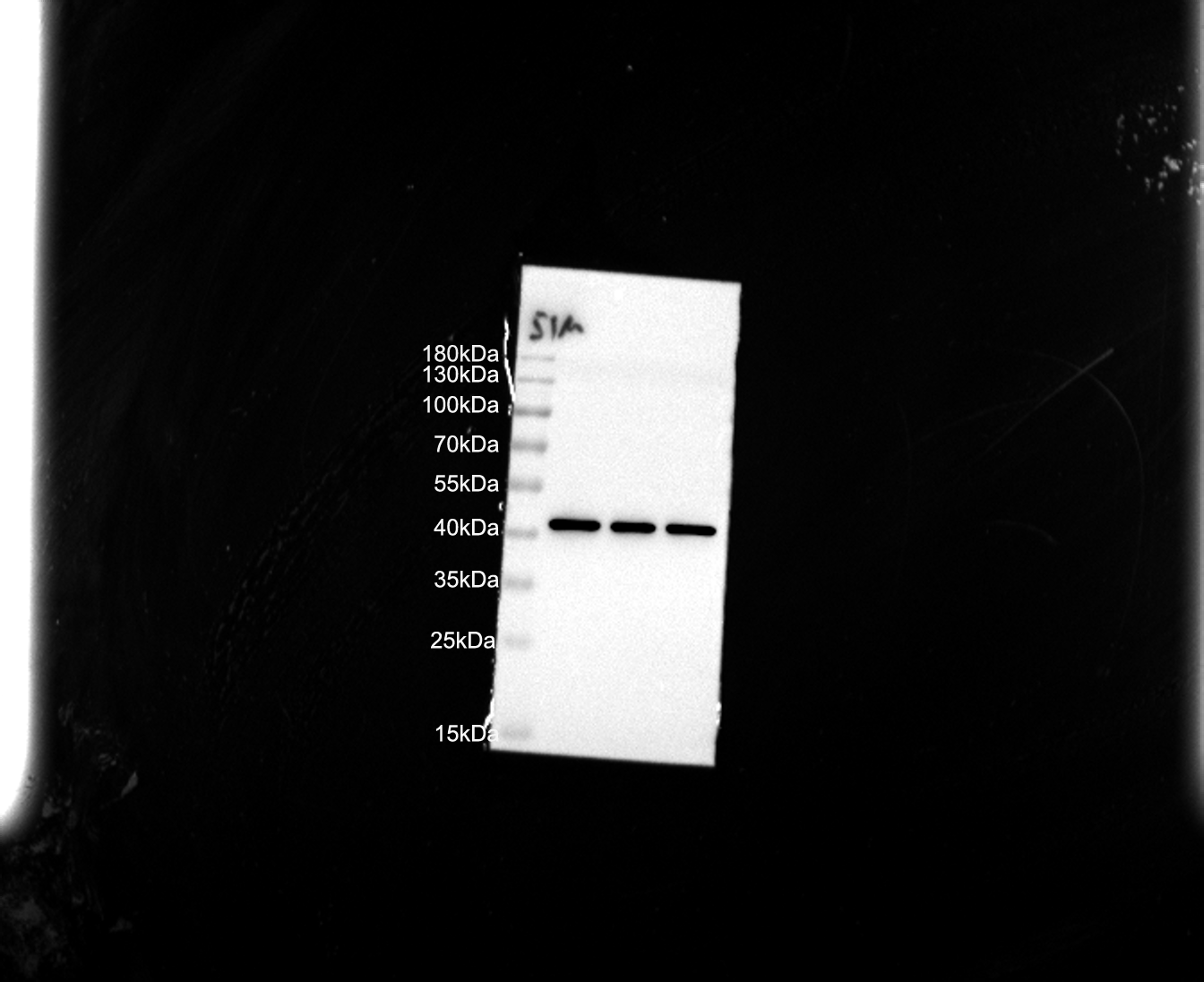

Supplement: S1 Raw data — (ZIP) [file pone.0317738.s002.zip › S1 Raw data/repeat 4/ACTIN..Tif]

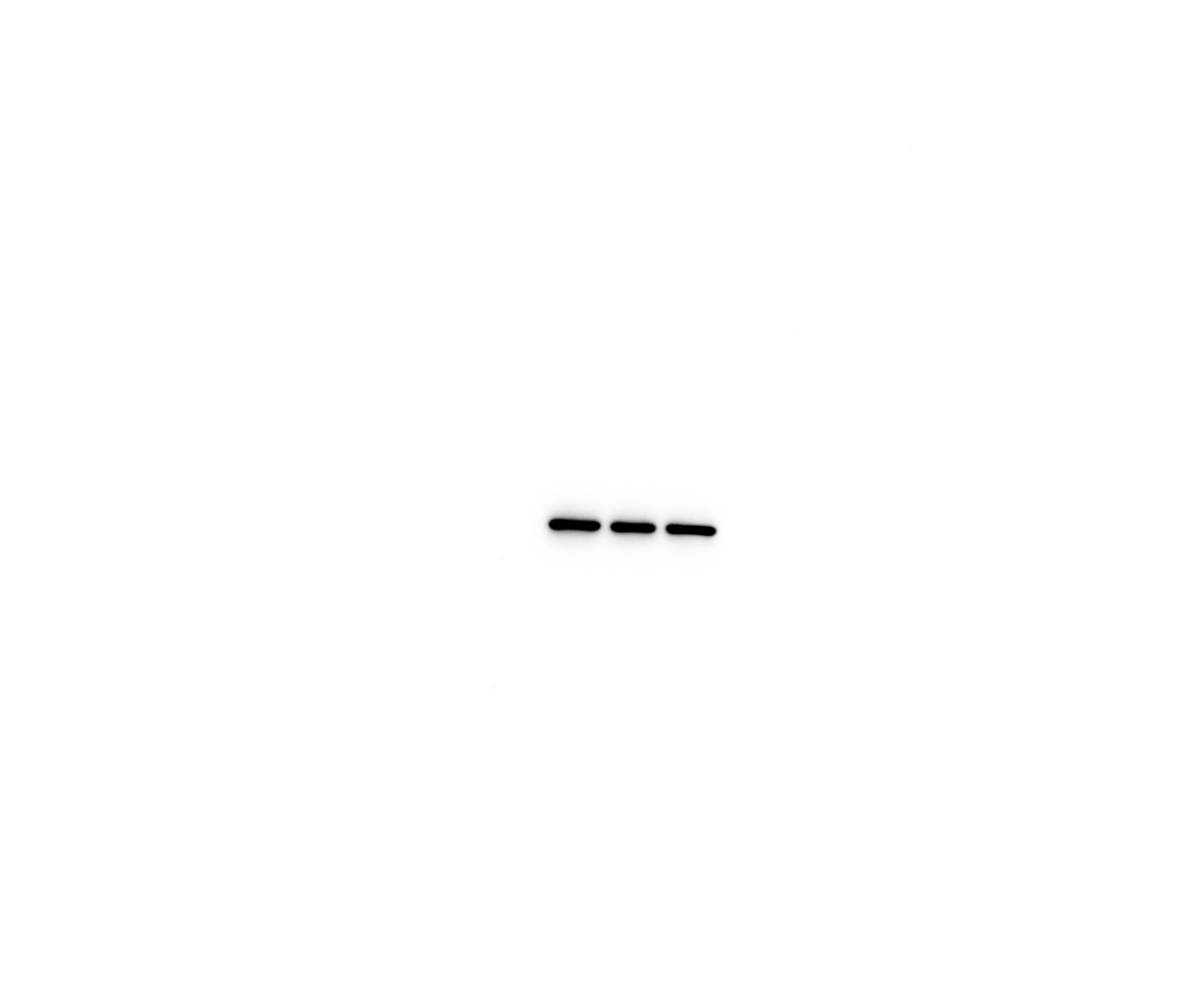

Supplement: S1 Raw data — (ZIP) [file pone.0317738.s002.zip › S1 Raw data/repeat 4/ACTIN.Tif]

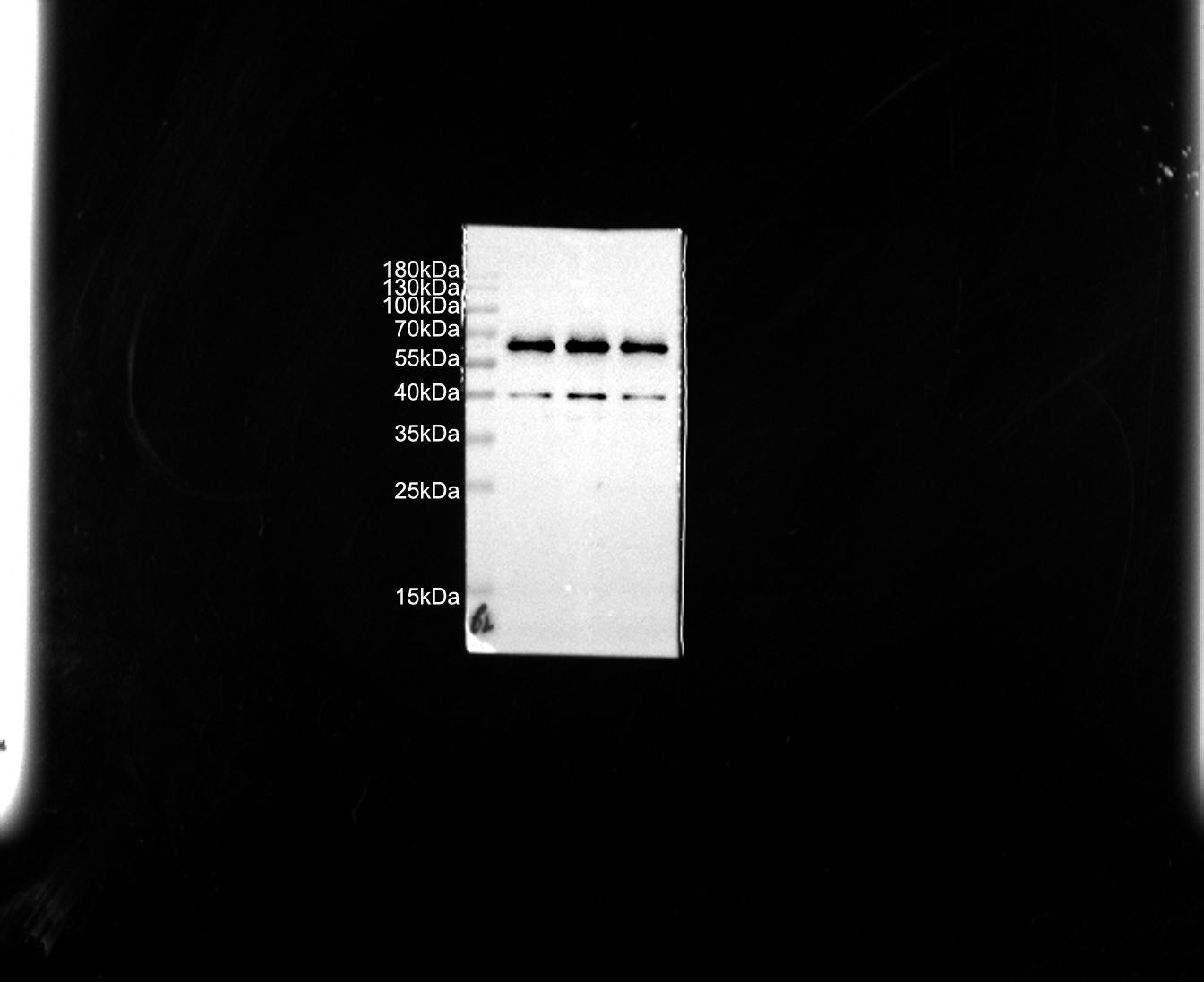

Supplement: S1 Raw data — (ZIP) [file pone.0317738.s002.zip › S1 Raw data/repeat 4/AKT..Tif]

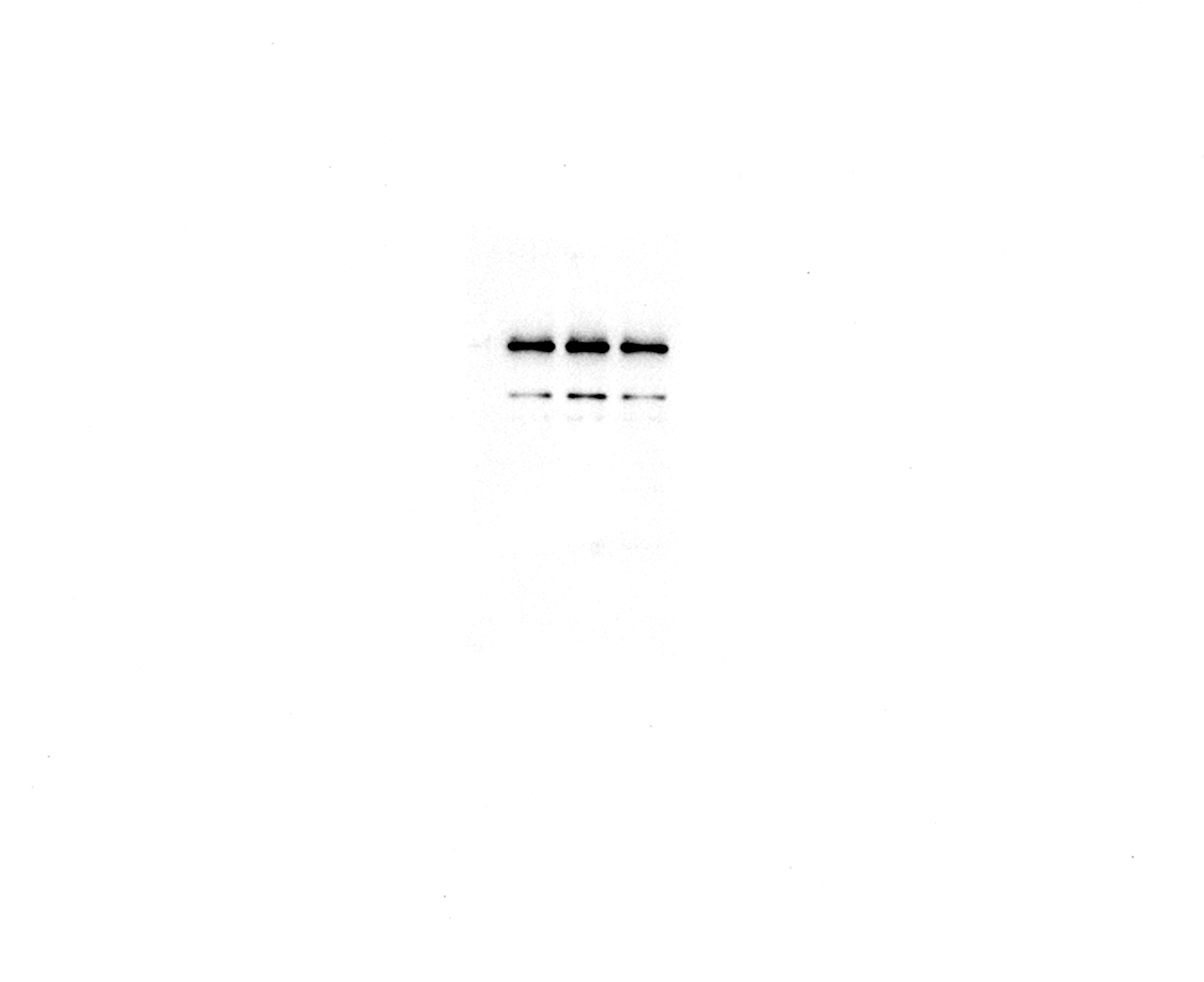

Supplement: S1 Raw data — (ZIP) [file pone.0317738.s002.zip › S1 Raw data/repeat 4/AKT.Tif]

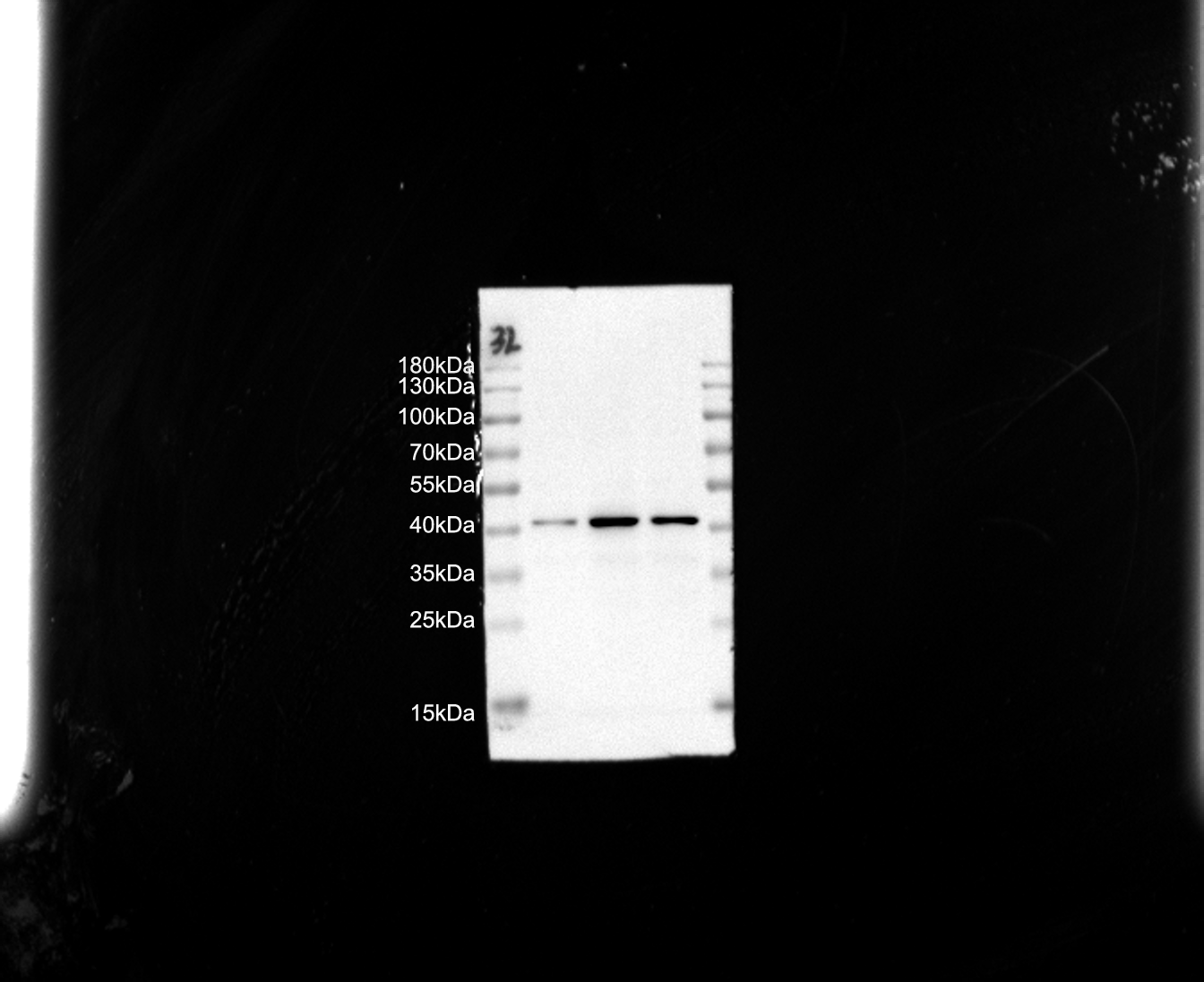

Supplement: S1 Raw data — (ZIP) [file pone.0317738.s002.zip › S1 Raw data/repeat 4/CAS9..Tif]

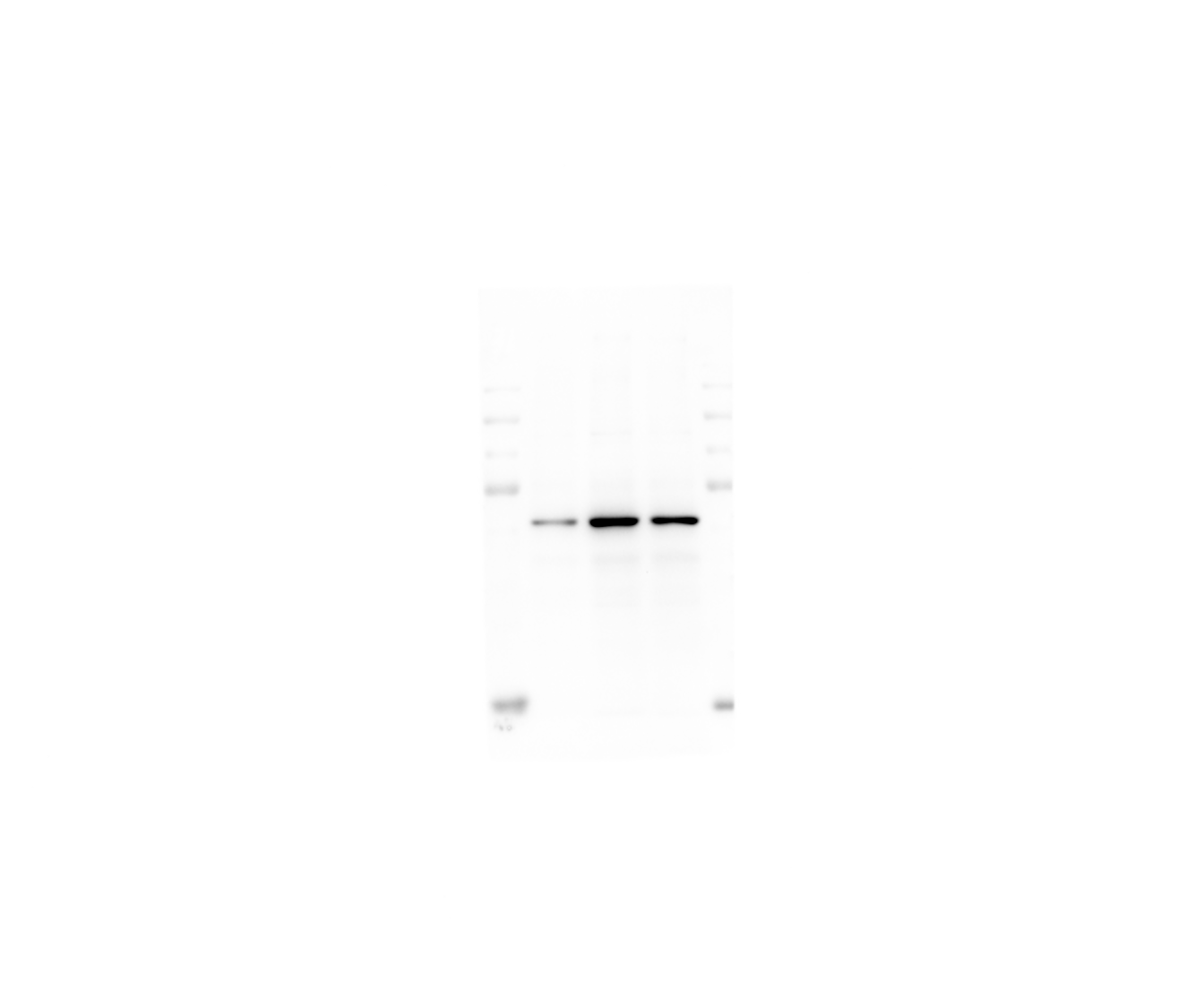

Supplement: S1 Raw data — (ZIP) [file pone.0317738.s002.zip › S1 Raw data/repeat 4/CAS9.Tif]

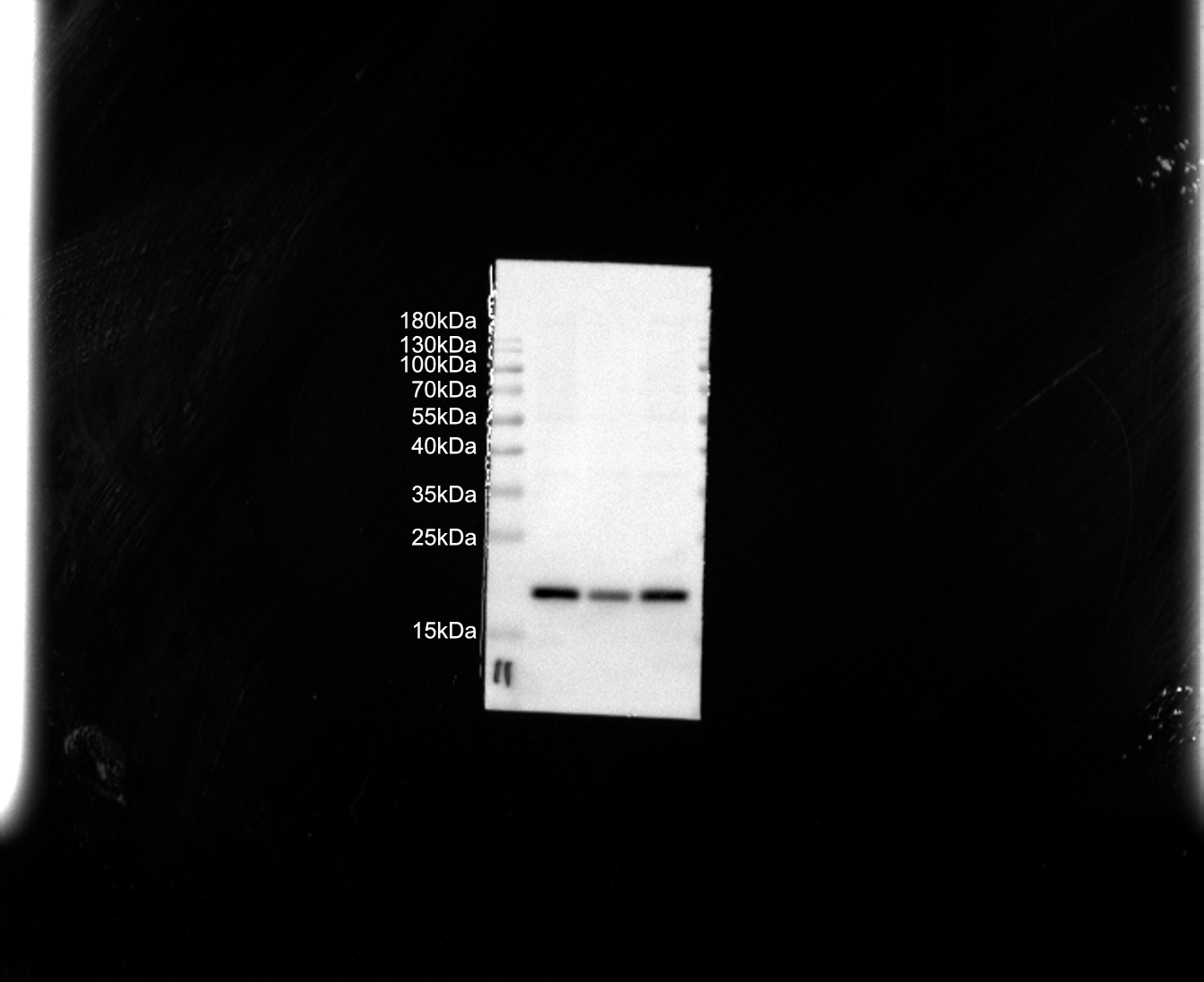

Supplement: S1 Raw data — (ZIP) [file pone.0317738.s002.zip › S1 Raw data/repeat 4/FGF1..Tif]

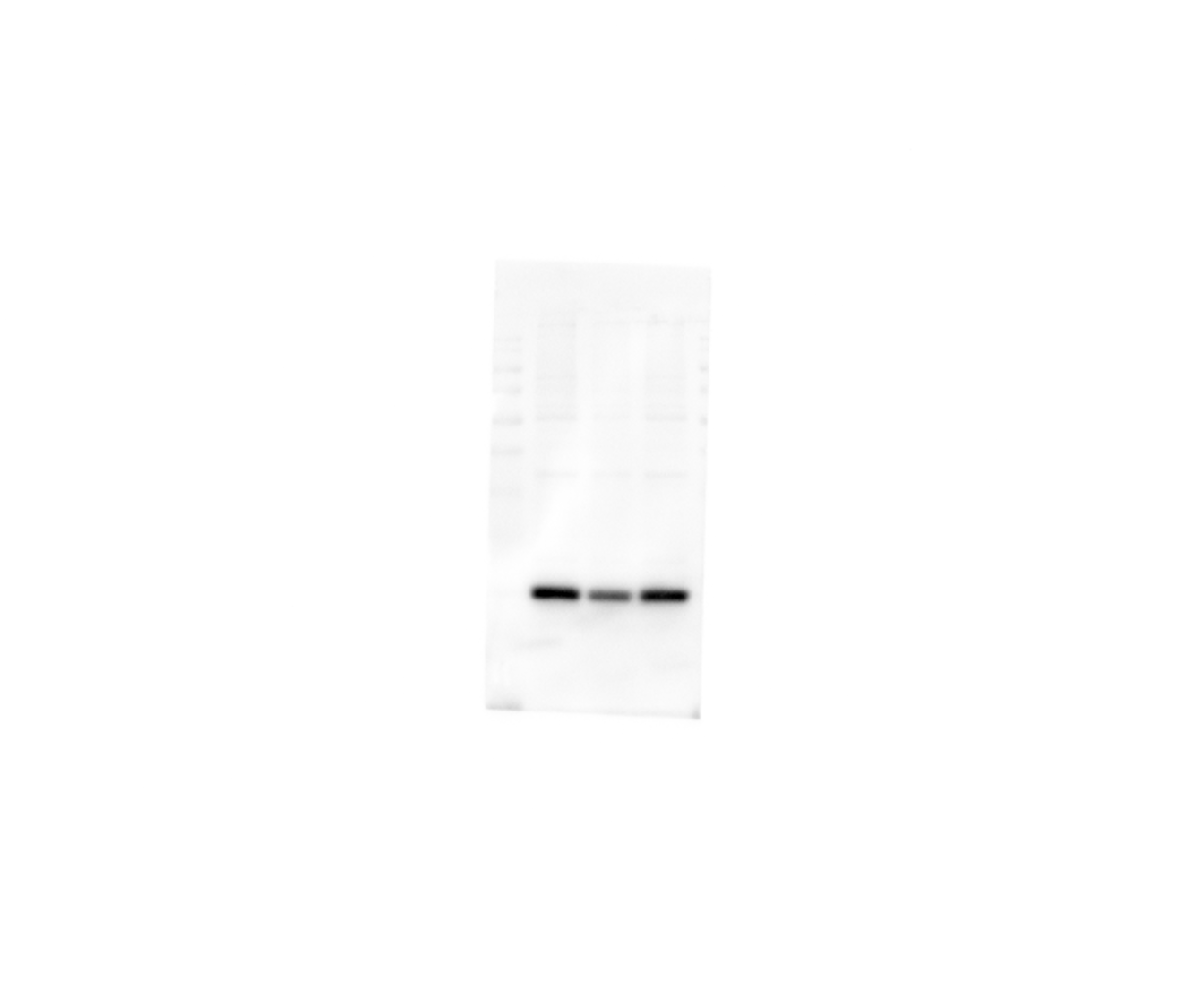

Supplement: S1 Raw data — (ZIP) [file pone.0317738.s002.zip › S1 Raw data/repeat 4/FGF1.Tif]

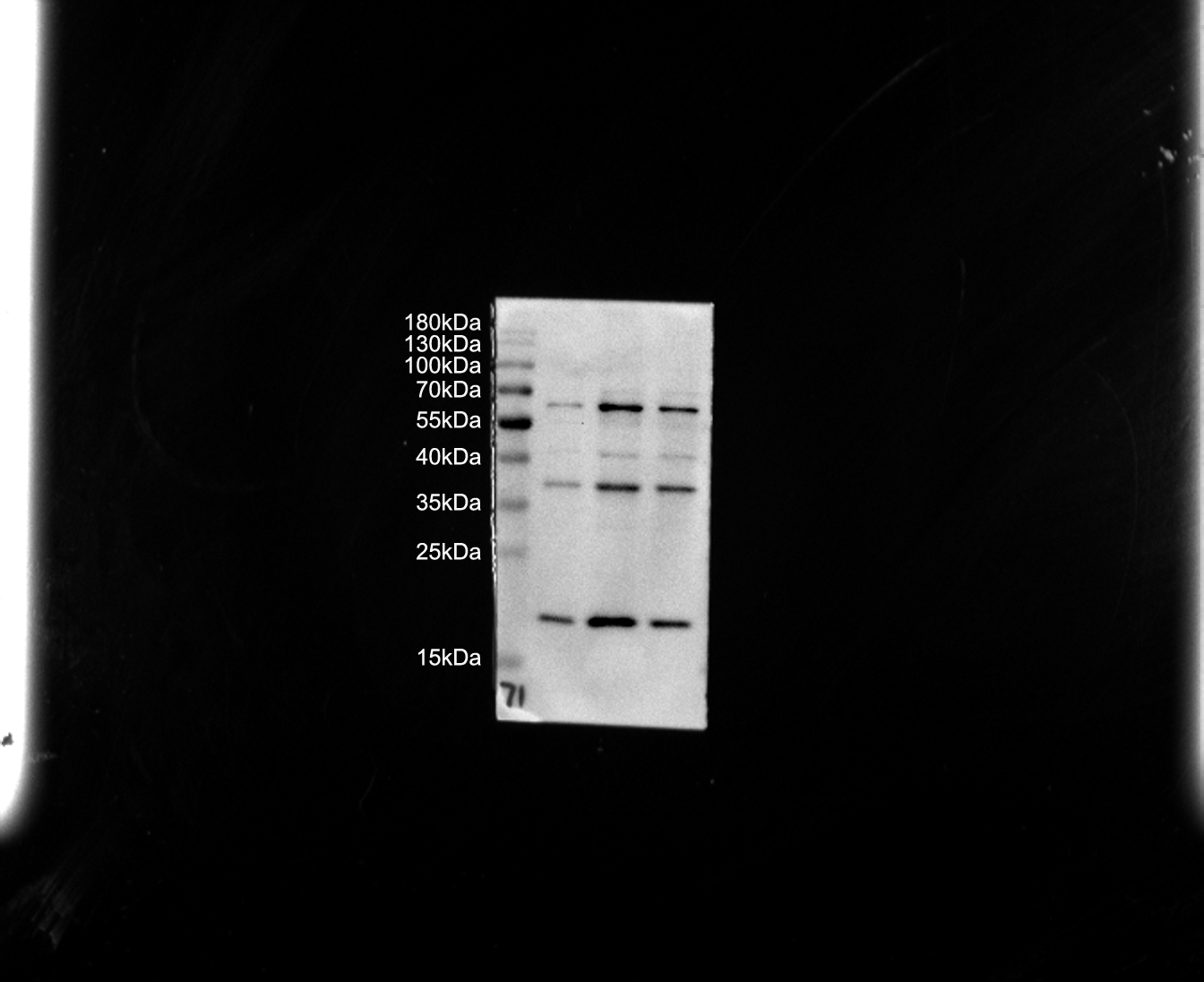

Supplement: S1 Raw data — (ZIP) [file pone.0317738.s002.zip › S1 Raw data/repeat 4/P-AKT..Tif]

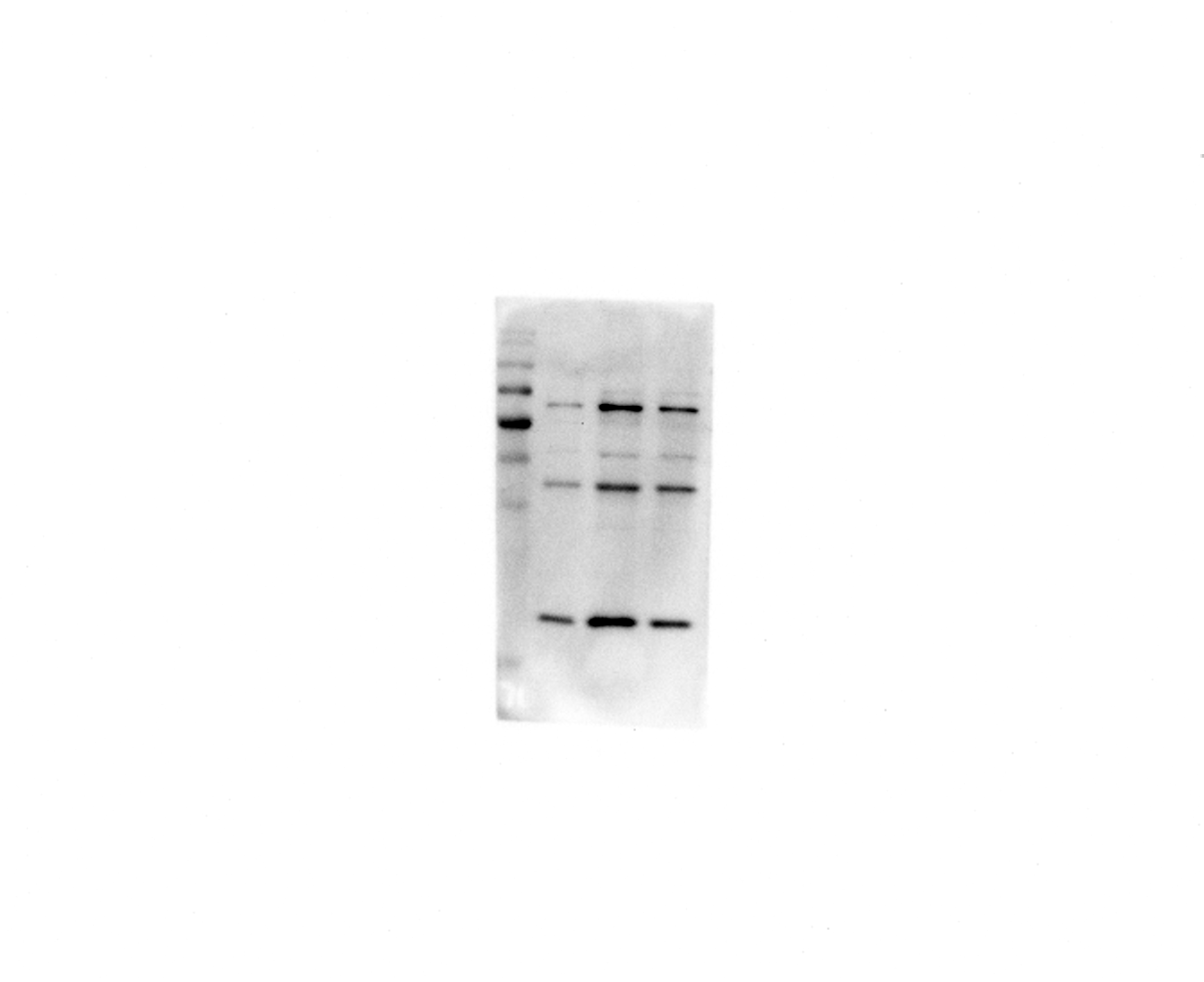

Supplement: S1 Raw data — (ZIP) [file pone.0317738.s002.zip › S1 Raw data/repeat 4/P-AKT.Tif]

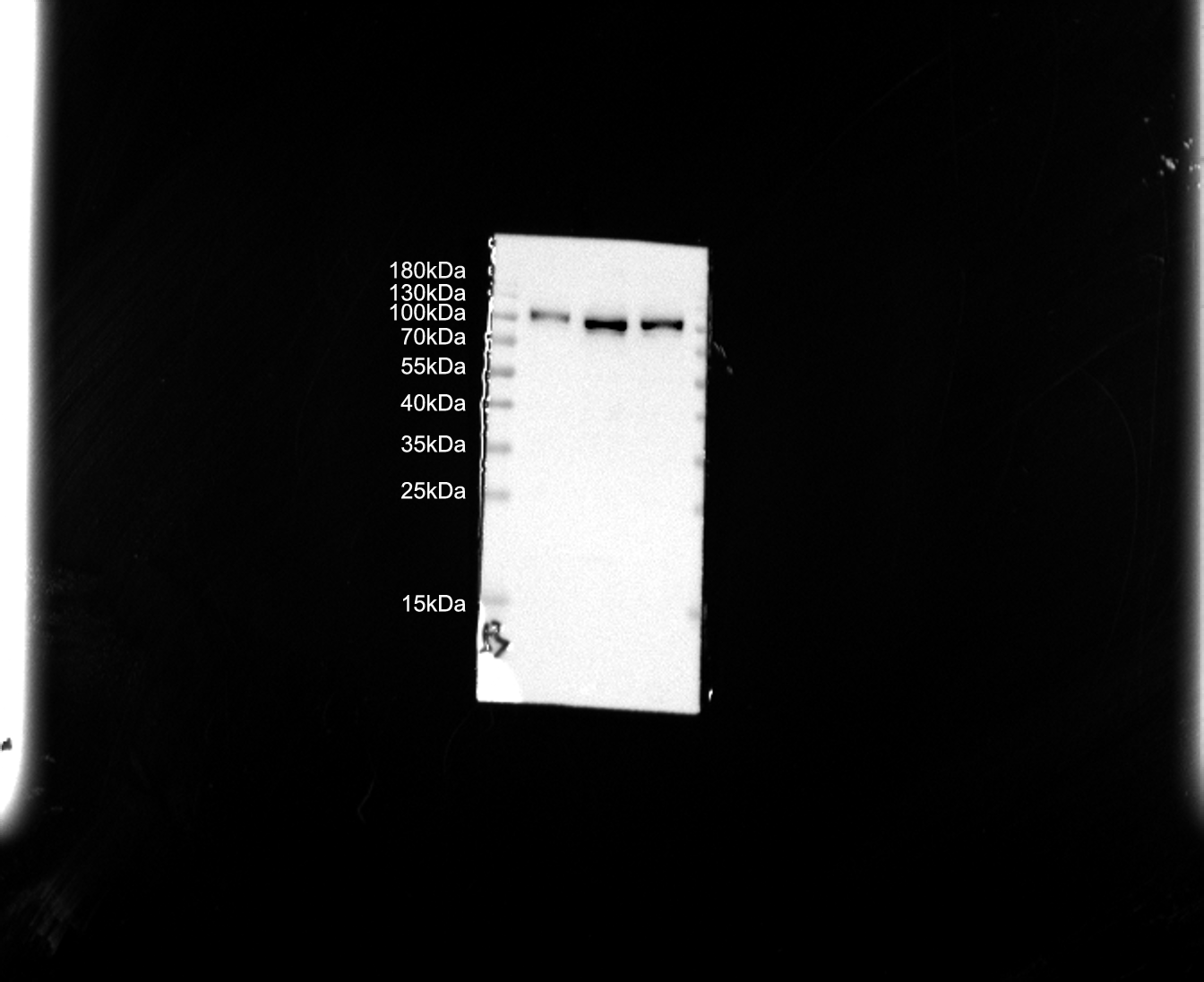

Supplement: S1 Raw data — (ZIP) [file pone.0317738.s002.zip › S1 Raw data/repeat 4/STAT3..Tif]

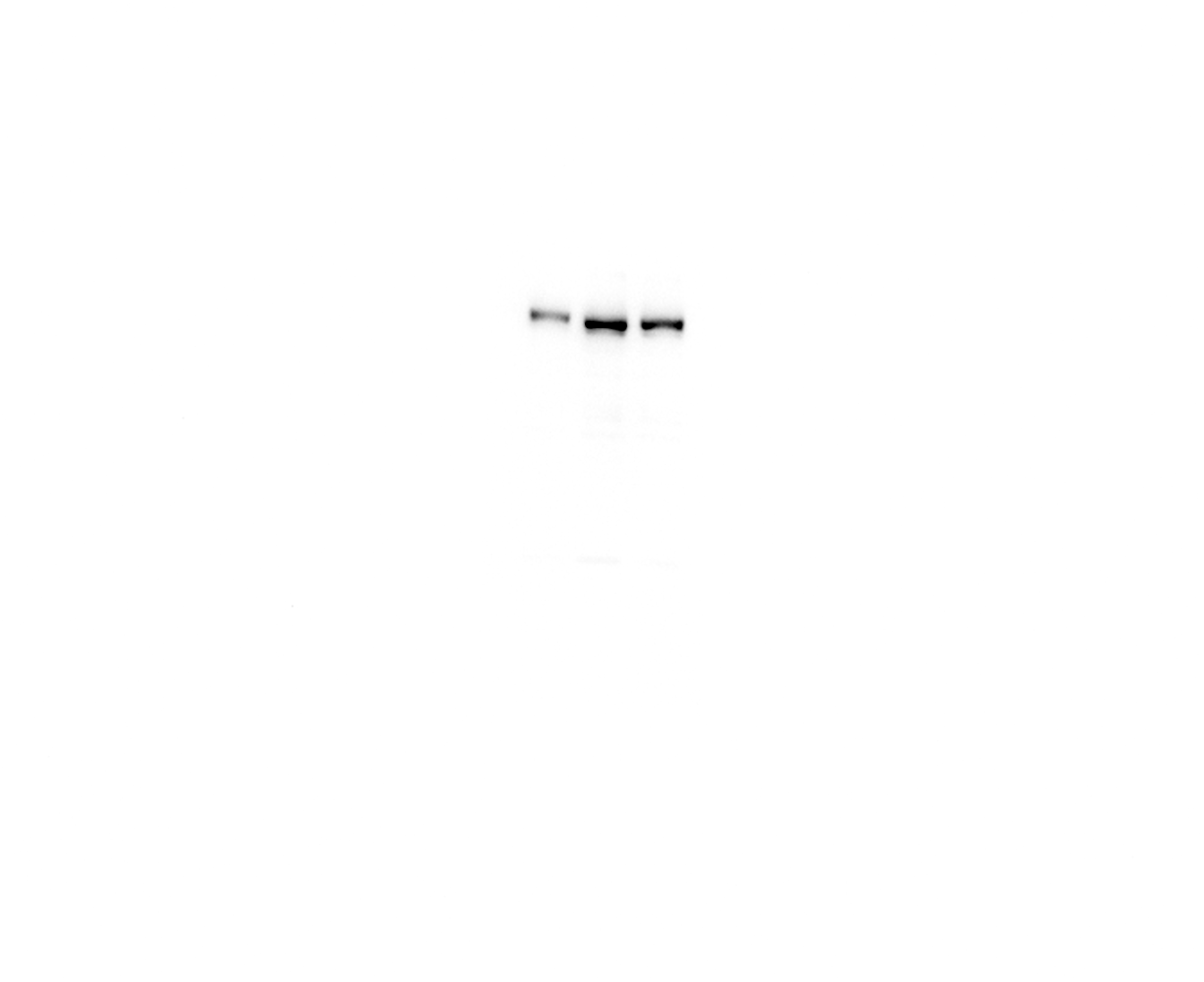

Supplement: S1 Raw data — (ZIP) [file pone.0317738.s002.zip › S1 Raw data/repeat 4/STAT3.Tif]
